# Integrative taxonomic revision of the land snail genus *Sarika* Godwin-Austen, 1907 in Thailand, with descriptions of nine new species (Eupulmonata, Ariophantidae)

**DOI:** 10.3897/zookeys.976.53859

**Published:** 2020-10-20

**Authors:** Arthit Pholyotha, Chirasak Sutcharit, Piyoros Tongkerd, Somsak Panha

**Affiliations:** 1 Biological Sciences Program, Faculty of Science, Chulalongkorn University, Bangkok 10330, Thailand Chulalongkorn University Bangkok Thailand; 2 Animal Systematics Research Unit, Department of Biology, Faculty of Science, Chulalongkorn University, Bangkok 10330, Thailand Chulalongkorn University Bangkok Thailand

**Keywords:** Diversity, DNA barcodes, Indochina, limestone, systematics

## Abstract

Members of the land snail genus *Sarika*Godwin-Austen 1907 are superficially similar and difficult to differentiate by their shell morphology so that their species limits are still unclear. In order to resolve the taxonomy of this group, a phylogenetic reconstruction of *Sarika* is presented, based on morphological and anatomical characters, as well as on partial sequences of the mitochondrial cytochrome c oxidase subunit I (COI) gene. In total, 23 species of *Sarika* are recognised in Thailand, and nine species are new to science, namely *S. caligina* Pholyotha & Panha, **sp. nov.**, *S. gratesi* Pholyotha & Panha, **sp. nov.**, *S. inferospira* Pholyotha & Panha, **sp. nov.**, *S. lactospira* Pholyotha & Panha, **sp. nov.**, *S. megalogyne* Pholyotha & Panha, **sp. nov.**, *S. melanospira* Pholyotha & Panha, **sp. nov.**, *S. pellosa* Pholyotha & Panha, **sp. nov.**, *S. solemi* Pholyotha & Panha, **sp. nov.**, and *S. subheptagyra* Pholyotha & Panha, **sp. nov.** Results from genital examination and COI analyses confirm the monophyly of *Sarika* and its species. The intra- and inter-specific genetic distances of *Sarika* were 0–3.7% and 4.6–12.0%, respectively. Colour images of the living adults, shell, and genitalia along with SEM images of the spermatophore and radula are given. In addition, an identification key and a geographical distribution map of *Sarika* species are provided.

## Introduction

Thailand contains a high diversity of land snails and high levels of species endemism, which is likely due to its position in the centre of the zoogeographical regions of Indo-Burma and Sundaland, a large extensive range of several limestone and mountainous hills, various kinds of tropical forests, and a high relative humidity (Myers et al. 2000; Gupta 2005; Clements et al. 2006; Naggs et al. 2006; Ridd et al. 2011; Gardner et al. 2015). To date, many groups of land snails in Thailand have been well studied, such as micro-snails (e.g., Tongkerd et al. 2004), the colourful tree snails (e.g., Sutcharit and Panha 2006), carnivorous snails (e.g., Siriboon et al. 2014), and operculate snails (e.g., Nantarat et al. 2014). However, information on the common ground-dwelling land snail genus *Sarika*Godwin-Austen 1907 is very scanty, so that the taxonomic status of most *Sarika* species is still doubtful and needs to be resolved.

*Sarika* is a land snail genus in the family AriophantidaeGodwin-Austen 1883 and is widely distributed in mainland Southeast Asia, especially throughout Thailand (Blanford and Godwin-Austen 1908; Zilch 1959; Solem 1966; Schileyko 2002; Inkhavilay et al. 2019; Pholyotha et al. 2020a, 2020c). Systematic studies of *Sarika* date back from the mid-19^th^ to the early 20^th^ centuries (Pfeiffer 1860; Möllendorff 1894, 1902; Godwin-Austen 1907; Blanford and Godwin-Austen 1908; Tomlin 1929; Laidlaw 1933; Solem 1966). The first genital examination of *Helix resplendens*Philippi 1846 was done by Godwin-Austen 1898. Nine years later, Godwin-Austen 1907 nominated *Sarika* as a distinct genus from the genus *Macrochlamys* Gray, 1847, and designated *H. resplendens*Philippi 1846 as the type species of *Sarika*, emphasizing that *Sarika* and *Macrochlamys* have a very similar shell form (depressed shape, closely coiled whorl, polished, thin, and semi-transparent) and general soft anatomy (short to long flagellum, long spermatheca and large dart apparatus). However, the genitalia of *Sarika* possess a straight (or un-coiled) epiphallic caecum, while *Macrochlamys* has a spirally coiled epiphallic caecum (Godwin-Austen 1907; Blanford and Godwin-Austen 1908; Schileyko 2002, 2003; Pholyotha et al. 2018, 2020c; Sajan et al. 2019).

Subsequentially, many species have been placed in the genus *Sarika* (Laidlaw 1933; Solem 1966; Panha 1996; Hemmen and Hemmen 2001; Pholyotha et al. 2018, 2020a, 2020c; Inkhavilay et al. 2019). Yet, most previous studies were either species descriptions or checklist, and in Thailand and Malaysia, only a few studies so far have dealt with the genital anatomy (Godwin-Austen 1907; Laidlaw 1933; Solem 1966; Maneevong 2000; Sutcharit and Panha 2008). In Laos, Inkhavilay et al. (2019) published a checklist of the land snails, including several species of *Sarika*. A year later, the genus *Sarika* from Myanmar and Cambodia were taxonomically revised, and two new species from Myanmar and three new species from Cambodia were discovered based on genitalia characters (Pholyotha et al. 2020a, 2020c; Sutcharit et al. 2020). However, knowledge on the genus *Sarika* is still fragmentary because most of the nominal species are described from Thailand. Therefore, we examined the anatomy of specimens collected during intensive field surveys throughout Thailand, and found that the diversity of this genus remains underestimated and its complex relationships are a mystery.

This present study is the first comprehensive taxonomic treatment of the land snail genus *Sarika* based on conchological and anatomical characters and data on the mitochondrial cytochrome c oxidase subunit I (COI) gene. The COI gene has been widely used to resolve the phylogenetic relationships among closely related species in several land snails (Liew et al. 2009; Nantarat et al. 2014; Köhler and Criscione 2015; Hyman and Köhler 2018). Thus, we analysed the COI gene phylogeny of *Sarika* to test the monophyly of each species and to understand the phylogenetic relationships of *Sarika*. This work includes taxonomic updates, illustrations of type specimens (when possible), and descriptions of the living snails, shells, genitalia, radula, and spermatophore. Moreover, nine *Sarika* species are herein described as new to science. An identification key to *Sarika* is provided and the distribution ranges for species are updated.

## Materials and methods

### Specimen sampling and morphological studies

This work is based on new specimens collected throughout Thailand and voucher specimens deposited in the Chulalongkorn University Museum of Zoology (CUMZ), Bangkok. Living specimens were photographed, euthanised by two-step methods (American Veterinary Medical Association 2020), and then fixed in 95% (v/v) ethanol for morphological and DNA studies. Species identification followed the original descriptions by Godwin-Austen 1907, Blanford and Godwin-Austen (1908) and Pholyotha et al. (2020c), and were then compared to the relevant type specimens. To study anatomy, 3–10 specimens of each species were dissected and examined under a stereomicroscope. Adult shells were used to measure the shell height and shell width, and to count the number of whorls. Shells and genitalia were imaged using a digital camera and a stereomicroscope with Cell’D Imaging Software. Radulae were extracted, soaked in 10% (w/v) sodium hydroxide, cleaned with distilled water, and then imaged by scanning electron microscopy (SEM; JEOL, JSM-6610 LV).

In the material examined sections, shells refer to empty shells while specimens refer to specimens preserved in ethanol. The Thai terms “Tham” meaning cave, “Wat” meaning temple, and “Phu” and “Khao” for mountain or hill are used throughout for the locality names.

Descriptions of all new species herein are attributed to Pholyotha & Panha.

### COI analyses

Details of samples selected for COI analysis are shown in Table 1. DNA was extracted from the foot muscle using the NucleoSpin Tissue Kit (Macherey-Nagel, Germany), following the standard procedure of the manufacturer. A fragment of the mitochondrial cytochrome c oxidase subunit I (COI) gene was amplified from each specimen by PCR using the universal primer pair LCO1491 (5′-GGTCAACAAATCATAAAGATATTGG-3′) and HCO2198 (5′-TAAACTTCAGGGTGACCAAAAAATCA-3′) (Folmer et al. 1994). The reaction was performed using standard protocols (Nantarat et al. 2014) with annealing temperatures of 50 °C or 52 °C for 120 s. The amplified PCR products were checked under an UV transilluminator after gel electrophoresis, then commercially sequenced by Bioneer Co., Korea, with the same primers in both directions. Nucleotide sequences were deposited in GenBank under accession numbers: MT894062–MT894119 (see Table 1).

**Table 1.** Information of all samples used for the phylogenetic study.

Species/ specimen code	CUMZ code	Locality	GenBank accession number
**Genus *Sarika***			
*S. resplendens*			
C14	7880	Bang Krachao, Phra Pradaeng, Samut Prakan, Thailand	MT894062
C36	7871	Wat Tham Pha Phung, Wang Pong, Phetchabun, Thailand	MT894063
E17	7875	Mountain area near Ang Kep Nam Dan Chumphon, Bo Rai, Trat, Thailand	MT894064
S4–3	7815	Limestone outcrop near Khanom Golden Beach, Khanom, Nakhon Si Thammarat, Thailand	MT894065
S42	7829	Wat Ao Sadet, Khanom, Nakhon Si Thammarat, Thailand	MT894066
W9	7886	Wat Tha Khanun, Thong Pha Phum, Kanchanaburi, Thailand	MT894067
W63	7867	Wat Buri Ratchawanaram, Pak Tho, Ratchaburi, Thailand	MT894068
*S. dohrniana*			
C18	7627	Wat Tham Tham Osot, Muak Lek, Saraburi, Thailand	MT894069
C32	7611	Wat Tham Mongkol Nimit, Mueang, Lopburi, Thailand	MT894070
NE16	7631	Mountain area near Lam Phra Phloeng Dam, Pak Thong Chai, Nakhon Ratchasima, Thailand	MT894071
*S. obesior*			
W5	7680	Khao Nang Panthurat, Cha-am, Phetchaburi, Thailand	MT894072
W52	7673	Wat Tham Rong, Ban Lat, Phetchaburi, Thailand	MT894073
W54	7676	Khao Lom Muak, Mueang, Prachuap Khiri Khan, Thailand	MT894074
W73	7684	Limestone outcrop in Kui Buri, Kui Buri, Prachuap Khiri Khan, Thailand	MT894075
*S. limbata*			
S41	7652	Wat Phut Sadi Phupharam, Thung Tako, Chumphon, Thailand	MT894076
S41–2	7652	Wat Phut Sadi Phupharam, Thung Tako, Chumphon, Thailand	MT894077
S55	7653	Wat Tham Khao Lan, Sawi, Chumphon, Thailand	MT894078
S55–2	7653	Wat Tham Khao Lan, Sawi, Chumphon, Thailand	MT894079
*S. heptagyra*			
W19	7231	Tham Khao Noi Bureau of Monks, Thong Pha Phum, Kanchanaburi, Thailand	MT364980
W25	7232	Tham Dao Wadung, Sai Yok, Kanchanaburi, Thailand	MT364981
W27	7279	Kroeng Krawia, Thong Pha Phum, Kanchanaburi, Thailand	MT894080
*S. kawtaoensis*			
S3	7762	Limestone outcrop near Khanom Seafood, Khanom, Nakhon Si Thammarat, Thailand	MT894081
S13	7760	Tham Nam Phut, Mueang, Phang-nga, Thailand	MT894082
S28	7759	Wat Suwan Khuha, Mueang, Phang-nga, Thailand	MT894083
S54	7709	Khao Phlu Cave, Pathio, Chumphon, Thailand	MT894084
S118	7738	Kaeo Surakan Cave, Lan Saka, Nakhon Si Thammarat, Thailand	MT894085
*S. caligina* sp. nov.			
C12	7245	Wat Tham Si Wilai, Chaloem Phra Kiat, Saraburi, Thailand	MT894086
C12–2	7245	Wat Tham Si Wilai, Chaloem Phra Kiat, Saraburi, Thailand	MT894087
C12–3	7245	Wat Tham Si Wilai, Chaloem Phra Kiat, Saraburi, Thailand	MT894088
*S. lactospira* sp. nov.			
S43	7287	Wat Ao Sadet, Khanom, Nakhon Si Thammarat, Thailand	MT894089
S43–2	7287	Wat Ao Sadet, Khanom, Nakhon Si Thammarat, Thailand	MT894090
S44	7291	Khao Krot Bureau of Monks, Khanom, Nakhon Si Thammarat, Thailand	MT894091
S44–2	7291	Khao Krot Bureau of Monks, Khanom, Nakhon Si Thammarat, Thailand	MT894092
*S. megalogyne* sp. nov.			
S60	7524	Limestone outcrop in Saphli, Pathio, Chumphon, Thailand	MT894093
S143	7909	Limestone outcrop in Saphli, Pathio, Chumphon, Thailand	MT894094
W58	7238	Khao Ma Rong Cave, Bang Saphan, Prachuap Khiri Khan, Thailand	MT364976
*S. subheptagyra* sp. nov.			
C6	7513	Hup Pa Tat, Lan Sak, Uthai Thani, Thailand	MT894095
C9	7507	Tham Namthip Bureau of Monks, Lan Sak, Uthai Thani, Thailand	MT894096
C34	7511	Hup Pa Tat, Lan Sak, Uthai Thani, Thailand	MT894097
*S. hainesi*			
C37	7272	Ched Khot Waterfall, Kaeng Khoi, Saraburi, Thailand	MT894098
E2	7237	Pang Sida Waterfall, Watthana Nakhon, Sa Kaeo, Thailand	MT894099
E2–3	7237	Pang Sida Waterfall, Watthana Nakhon, Sa Kaeo, Thailand	MT894100
*S. bocourti*			
E6	7579	Khao Soi Dao, Soi Dao, Chanthaburi, Thailand	MT894101
E8–2	7596	Trok Nong Waterfall, Khlung, Chanthaburi, Thailand	MT894102
E19	7594	Mountain area near Wat Ban Wang Ka Prae, Pong Nam Ron, Chanthaburi, Thailand	MT894103
S7	7592	Mountain area near Khao Sok Evergreen House, Phanom, Surat Thani, Thailand	MT894104
*S. inferospira* sp. nov.			
NE6–2	7254	Wat Tham Sai Thong, Nong Kung Si, Kalasin, Thailand	MT894105
NE6–3	7254	Wat Tham Sai Thong, Nong Kung Si, Kalasin, Thailand	MT894106
NE25	7257	Wat Tham Sai Thong, Nong Kung Si, Kalasin, Thailand	MT894107
*S. melanospira* sp. nov.			
E28	7243	Wat Tham Suwan Phu Pha, Khao Chamao, Rayong, Thailand	MT894108
E28–2	7243	Wat Tham Suwan Phu Pha, Khao Chamao, Rayong, Thailand	MT894109
E28–3	7243	Wat Tham Suwan Phu Pha, Khao Chamao, Rayong, Thailand	MT894110
*S. pellosa* sp. nov.			
E14	7519	Tham Phet Pho Thong, Khlong Hat, Sa Kaeo, Thailand	MT894111
E40–2	7520	Wat Tham Khao Chakan, Khao Chakan, Sa Kaeo, Thailand	MT894112
E46	7250	Tham Saeng Thian, Khlong Hat, Sa Kaeo, Thailand	MT894113
*S. dugasti*			
N11	7547	Wat Tham Tong, Chom Thong, Chiang Mai, Thailand	MT894114
N12	7563	Wat Tham Rakhang, Si Samrong, Sukhothai, Thailand	MT894115
W41–2	7574	Chao Por Phawo Shrine, Mae Sot, Tak, Thailand	MT894116
*S. solemi* sp. nov.			
N18	7298	The limestone karsts with dry forest near Mae La Na Cave, Pang Mapha, Mae Hong Son, Thailand	MT894117
N64	7503	Kew Mae Pan, Chom Thong, Chiang Mai, Thailand	MT894118
N67–2	7911	Mountain area in Chom Thong, Chiang Mai, Thailand	MT894119
**Genus *Taphrenalla***			
*T. asamurai*			
S18	7153	Wat Tham Wararam, Phanom, Surat Thani, Thailand	MT364934
S34	7153	Wat Tham Wararam, Phanom, Surat Thani, Thailand	MT364936
*T. diadema*			
S46	7175	Wat Tham Sumano, Srinagarindra, Phatthalung, Thailand	MT364940
S62	7181	Limestone outcrops at Khlong Chaloem, Kong Ra, Phatthalung, Thailand	MT364941
**Genus *Macrochlamys***			
*M. aspides*			
MY8	7135	Lun Nya Mountain, Hpa an, Kayin, Myanmar	MT364986
*M. caverna*			
C15	7113	Khao Mon Ing Dharma Practice Place, Ban Mi, Lopburi, Thailand	MT364988
C16	7111	Wat Tham Chang Pueak, Tha Wung, Lopburi, Thailand	MT364987
*Macrochlamys* sp.			
NE10	7910	Khao Kradong, Mueang, Buriram, Thailand	MT906154
**Genus *Hemiplecta***			
*H distincta*			
H54	5267	Tad Pha Suam, Paksong, Champasak, Laos	MT654617
*H humphreysiana*			
H7	–	Singapore	MT364994
*H pluto*			
H63	–	Laos	MT364995

The COI gene sequences were edited and aligned using ClustalW, as implemented in the MEGA7 software (Kumar et al. 2016). Genetic distances between *Sarika* species and related taxa were calculated using Kimura’s two-parameter model (K2P) as implemented in MEGA7 (Kumar et al. 2016). The alignments were tested for substitution saturation using DAMBE (Xia 2013). As no saturation was detected in the sequences (Iss < Iss.c with p < 0.01), all the codon positions were used in the subsequent analysis. The phylogenetic analyses were conducted using maximum likelihood (ML) and Bayesian inference (BI) and both analyses were performed on-line through the CIPRES Science Gateway (Miller et al. 2010). The ML analyses were performed by using the program RAxML-HPC2 on XSEDE v. 8.2.12 (Stamatakis 2014) with 1000 bootstrap replicates using GTRGAMMA as the model. Prior to the BI analysis, Kakusan4 (Tanabe 2011) identified the best-fit model as follows: the general time reversible model (Tavaré 1986) with gamma distribution for the first and the second COI codon positions, and the HKY model (Hasegawa et al. 1985) for the third COI codon position. The BI analysis was performed by using the program MrBayes on XSEDE v. 3.2.7a (Ronquist et al. 2012) with two simultaneous runs. The analysis was run for 10 million generations (default heating parameter), sampled every 500 generations, starting with a random tree and burn-in set to 50%. Convergence of the two runs was achieved if the average standard deviation of split frequencies were ≤ 0.01 (Ronquist et al. 2012). Bootstrap support values (BS) of ≥ 70% and BI posterior probabilities (PP) of ≥ 0.95 were regarded as significant (Hillis and Bull 1993; Felsenstein 2004; Huelsenbeck and Rannala 2004; Mauro and Agorreta 2010; Hirano et al. 2018).

### Anatomical abbreviations

In the descriptions of the genitalia, the term ‘proximal’ refers to the region closest to the genital opening, while ‘distal’ refers to the region furthest away from the genital opening. The following abbreviations were used as defined by Godwin-Austen 1907, Blanford and Godwin-Austen (1908), Solem (1966), Sutcharit and Panha (2008), and Pholyotha et al. (2018, 2020b):

**ant-ldl** anterior left dorsal lobe;

**at** atrium;

**da** dart apparatus;

**e** epiphallus;

**ec** epiphallic caecum;

**fl** flagellum;

**fo** free oviduct;

**gd** gametolytic duct;

**gs** gametolytic sac;

**hf** head filament;

**lsl** left shell lobe;

**p** penis;

**pc** penial caecum;

**post-ldl** posterior left dorsal lobe;

**pp** penial pilaster;

**prm** penial retractor muscle;

**psv** pseudo-verge;

**pv** penial verge;

**rdl** right dorsal lobe;

**rsl** right shell lobe;

**ss** sperm sac;

**tf** tail filament;

**v** vagina;

**vd** vas deferens.

### Institutional abbreviations

**CUMZ** Chulalongkorn University, Museum of Zoology, Bangkok, Thailand

**MNHN** Muséum National ďHistoire Naturelle, Paris, France

**NHM**, **NHMUK** The Natural History Museum, London, United Kingdom

**NMW** National Museum of Wales, Cardiff, Wales

**SMF** Forschungsinstitut und Naturmuseum Senckenberg, Frankfurt am Main, Germany

**ZRC** Zoological Reference Collection of the Lee Kong Chian Natural History Museum, National University of Singapore, Singapore

## Results

### COI analyses

**Figure 1.**
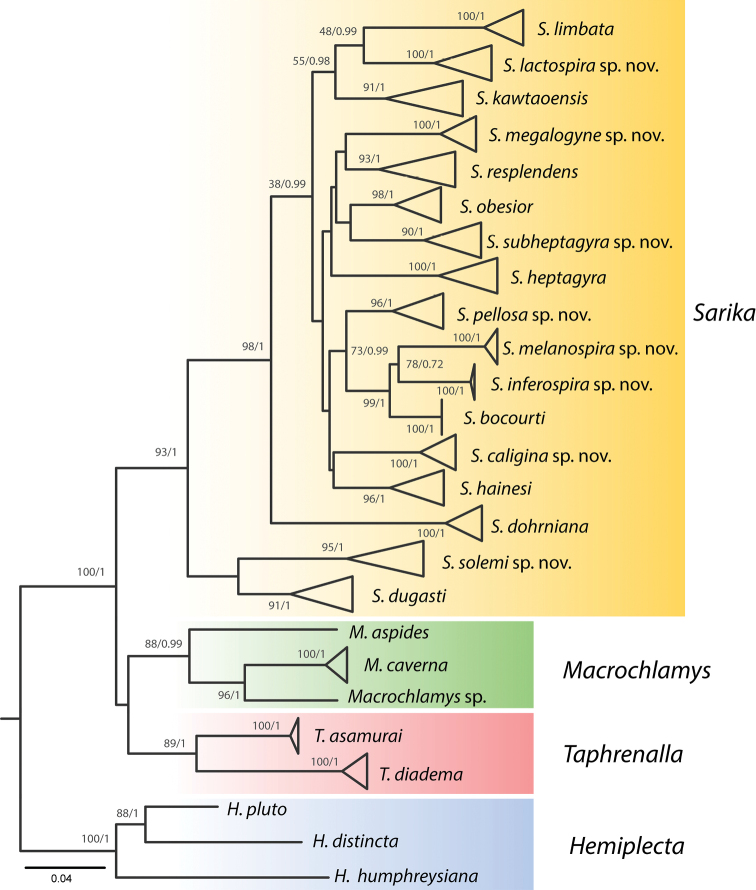
Maximum likelihood tree showing the relationships among species of *Sarika* based on the mitochondrial COI gene sequences. Numbers by the nodes are the ML bootstrap values (left) and Bayesian posterior probabilities (right); shown only for the nodes supported by ML or BI (≥ 70% and ≥ 0.95). Each clade colour refers to a genus.

The partial COI gene sequence data set included 61 specimens of *Sarika* as well as eleven sequences from *Macrochlamys*, *Taphrenalla* Pholyotha & Panha, 2020 and *Hemiplecta*Albers 1850, which were included as outgroups. The alignment of the COI gene fragments had a length of 655 base pairs. The obtained phylogenetic tree (Fig. 1) recovered 17 species of *Sarika* forming a well-supported clade, including the type species *S. resplendens* and eight new species with high support (BS = 93%, PP = 1). Even though the phylogenetic relationships among taxa were poorly resolved, the members of *Sarika* were always retrieved as a monophyletic clade. A clade of *Sarika* appears as a sister group of *Macrochlamys* + *Taphrenalla*. Together *Sarika* + *Taphrenalla* + *Macrochlamys* formed a well-supported clade (BS = 100%, PP = 1).

The mean genetic distances of the COI gene observed among *Sarika*, *Taphrenalla*, *Macrochlamys*, and *Hemiplecta* ranged from 9.8% (*Taphrenalla* and *Macrochlamys*) to 13.7% (*Sarika* and *Hemiplecta*). Intraspecies divergences within the genus *Sarika* ranged from 0% (*S. bocourti*) to 3.7% (*S. dugasti*), and interspecies divergences ranged from 4.6% (*S. bocourti* and *S. inferospira* sp. nov.) to 12.0% (*S. dugasti* and *S. limbata*), respectively (Table 2).

**Table 2.** Mean intra-specific and inter-specific genetic divergences among species of *Sarika* from the mitochondrial COI gene sequences estimated by the K2P model. Taxa in **bold** are the new species described herein.

*Sarika* spp.	1	2	3	4	5	6	7	8	9	10	11	12	13	14	15	16	17
1 *S. resplendens*	0.018																
2 *S. dohrniana*	0.100	0.017															
3 *S. obesior*	0.059	0.098	0.030														
4 *S. limbata*	0.085	0.106	0.086	0.012													
5 *S. heptagyra*	0.077	0.104	0.070	0.094	0.035												
6 *S. kawtaoensis*	0.069	0.097	0.074	0.072	0.078	0.033											
7 ***S. caligina***	0.075	0.088	0.063	0.086	0.072	0.073	0.011										
8 ***S. lactospira***	0.075	0.101	0.070	0.075	0.093	0.069	0.072	0.023									
9 ***S. megalogyne***	0.064	0.104	0.068	0.078	0.071	0.066	0.074	0.071	0.014								
10 ***S. subheptagyra***	0.065	0.087	0.063	0.090	0.079	0.074	0.082	0.081	0.069	0.024							
11 *S. hainesi*	0.066	0.091	0.064	0.074	0.074	0.070	0.057	0.073	0.066	0.073	0.024						
12 *S. bocourti*	0.069	0.104	0.069	0.092	0.079	0.071	0.071	0.081	0.076	0.075	0.070	0					
13 ***S. inferospira***	0.078	0.111	0.074	0.086	0.081	0.083	0.069	0.088	0.079	0.089	0.069	0.046	0.001				
14 ***S. melanospira***	0.080	0.103	0.080	0.097	0.094	0.082	0.081	0.086	0.074	0.082	0.076	0.051	0.053	0.005			
15 ***S. pellosa***	0.064	0.086	0.065	0.086	0.071	0.070	0.060	0.072	0.070	0.071	0.057	0.053	0.065	0.066	0.021		
16 *S. dugasti*	0.097	0.109	0.103	0.120	0.112	0.111	0.102	0.107	0.104	0.108	0.101	0.107	0.119	0.109	0.101	0.037	
17 ***S. solemi***	0.097	0.110	0.104	0.118	0.110	0.109	0.089	0.110	0.097	0.096	0.102	0.103	0.110	0.105	0.091	0.078	0.029

### Systematics

#### Family Ariophantidae Godwin-Austen, 1883

##### Genus Sarika

Taxon classificationAnimaliaStylommatophoraAriophantidae

Godwin-Austen, 1907

9DA96865-12CA-5795-A7A7-C7637D9B041D

Sarika Godwin-Austen 1907: 179. Blanford and Godwin-Austen 1908: 276. Zilch 1959: 325. Solem 1966: 36. Schileyko 2002: 1288. Sutcharit and Panha 2008: 96. Pholyotha et al. 2020c: 13, 14. Pholyotha et al. 2020a: 5.

###### Type species.

*Helix resplendens*Philippi 1846, by original designation in Godwin-Austen 1907: 179.

###### Diagnostic description.

***Shell*** thin to moderately solid, semi-translucent, pale milky to brown, depressed discoidal to globosely depressed with 5–8 convex whorls. Shell surface smooth, glossy, with very fine growth lines. Body whorl rounded, angulated to shouldered. Aperture crescentic with simple lip or rarely expanded lip. Umbilicus narrowly opened.

***Genitalia*** with penial retractor muscle attached to tip of epiphallic caecum; penis generally without penial verge, except for *S. consepta* (Benson 1860) and *S. nana* Pholyotha & Panha, 2020 (see Pholyotha et al. 2020a, 2020c), rarely present pseudo-verge; flagellum short to long; gametolytic duct long; dart apparatus large cylindrical.

***Spermatophore*** long and needle-shaped with three recognizable sections: (i) head filament rather short, (ii) cylindrical sperm sac containing sperm mass, and (iii) tail filament long thick walled tube with small hole in cross section and several spines present.

***Radular teeth*** with symmetrical tricuspid central tooth, asymmetrical tricuspid lateral teeth, and bicuspid marginal teeth.

***Species of Sarika*** with well-developed mantle edge (mantle lobe) with four lobes (one shell lobe and three dorsal lobes) or five lobes (two shell lobes and three dorsal lobes); sole tripartite, lateral foot margin, caudal foss, and caudal horn present.

**Figure 2.**
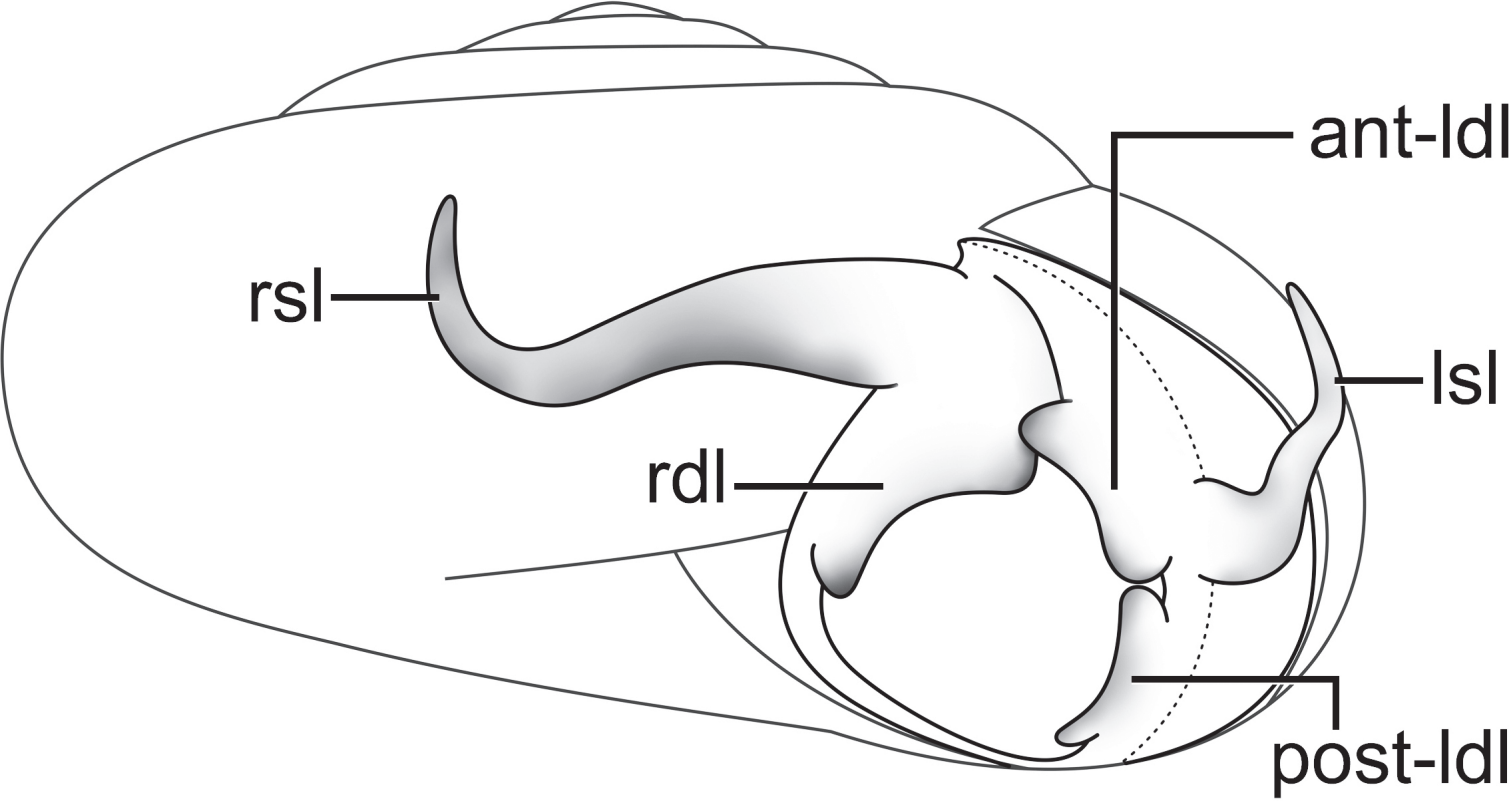
Schematic drawing of the mantle terminology of *Sarika* showing five mantle lobes (two shell lobes and three dorsal lobes).

###### Remarks.

All species of *Sarika* whose genital anatomy is known have a straight (un-coiled) epiphallic caecum and can be divided into three species groups. This informal subdivision is based on the number of mantle lobes, structure of genitalia and spermatophore (when available). It may be helpful as an alternative aid to identification.

**Figure 3.**
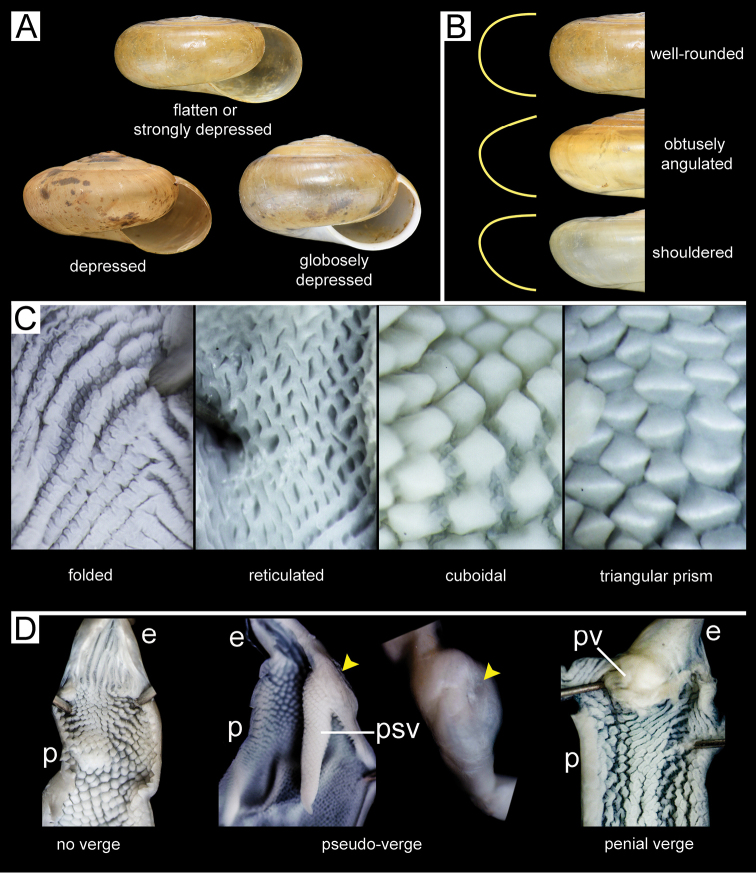
An illustrated synopsis of the shell and penis. **A** shell shape **B** whorl periphery **C** inner sculpture of the penis **D** appendage inside penis (no penial verge, pseudo-verge, and penial verge). Yellow arrowheads indicate the invagination of the penial wall.

**Figure 4.**
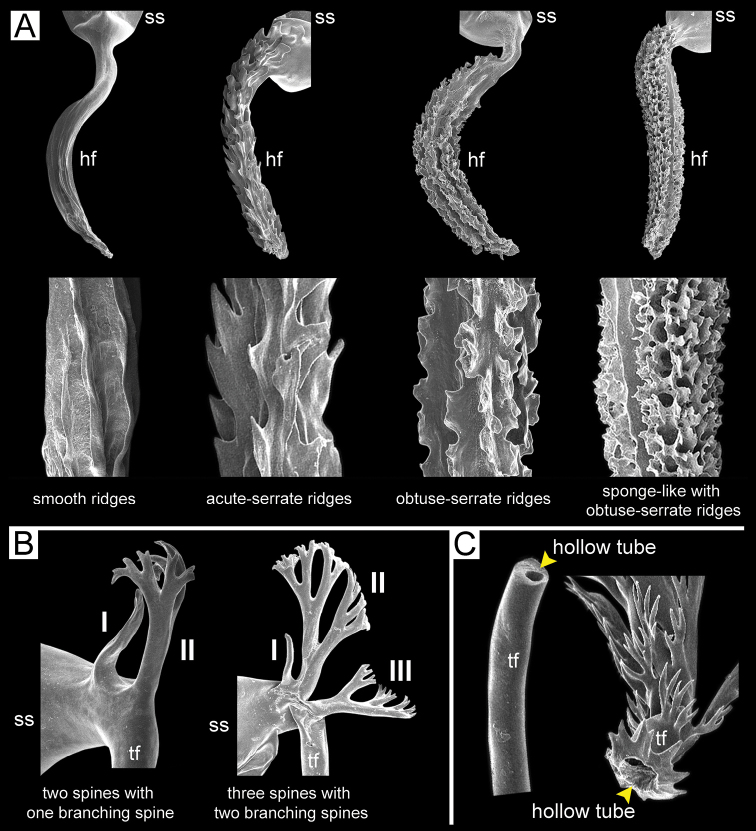
An illustrated synopsis of the spermatophore. **A** sculpture of head filament **B** branching spines on the region near the sperm sac of the tail filament **C** cross section of tail filament with a hollow tube.

**Group I: *Sarika resplendens* group.** Has five mantle lobes (with left shell lobe; Figs 2, 9A), penis without penial verge and pseudo-verge (Fig. 3D) and spermatophore usually with three spines on the connection between sperm sac and tail filament (Fig. 4B). This group comprises 10 species: *S. resplendens*, *S. dohrniana*, *S. obesior*, *S. limbata*, *S. heptagyra*, *S. kawtaoensis*, *S. caligina* sp. nov., *S. lactospira* sp. nov., *S. megalogyne* sp. nov., and *S. subheptagyra* sp. nov.

**Group II: *Sarika hainesi* group.** Has four mantle lobes (left shell lobe wanting; Fig. 33A), penis without penial verge and pseudo-verge, and spermatophore usually with two spines on connection between sperm sac and tail filament (Fig. 4B). This group comprises five species: *S. hainesi*, *S. bocourti*, *S. inferospira* sp. nov., *S. melanospira* sp. nov., and *S. pellosa* sp. nov.

**Group III: *Sarika dugasti* group.** Has five mantle lobes as in group I, penis with pseudo-verge (Fig. 3D), which probably originates from an invagination of the penial wall to become a large papilla (penial verge like). Unfortunately, no information about the spermatophore. This group comprises two species: *S. dugasti* and *S. solemi* sp. nov.

### Key to species of the genus *Sarika*Godwin-Austen 1907 in Thailand

This identification key is mainly based on the characters of genitalia and spermatophores, and is based on some taxonomic informative characters of shells.

**Table:** 

1	Apertural lip straight and simple or very slightly thickened in old specimens	**2**
–	Apertural lip at periphery with invagination of triangular lip (beak-like)	***S. gratesi* sp. nov.**
2	Penis with pseudo-verge (Fig. 3D)	**3**
–	Penis without pseudo-verge and penial verge (Fig. 3D)	**4**
3	Shell globosely depressed and well-rounded body whorl	***S. dugasti***
–	Shell depressed and angular body whorl	***S. solemi* sp. nov.**
4	Mantle edge with two shell lobes (left and right shell lobes; Figs 2, 9A)	**5**
–	Mantle edge with only right shell lobe (left shell lobe absent; Fig. 33A)	**14**
5	Inner wall of penis with reticulated pilasters (Fig. 3C)	**6**
–	Inner wall of penis with other types of pilasters (Fig. 3C)	**7**
6	Inner wall of distal part of penis has irregularly oblique folds (Fig. 22B)	***S. kawtaoensis***
–	Inner wall of distal part of penis has small cuboidal (Fig. 18B)	***S. limbata***
7	Inner wall of penis with triangular prism pilasters (Fig. 3C)	**8**
–	Inner wall of penis with cuboidal pilasters (Fig. 3C)	**12**
8	Body whorl slightly rounded to shouldered (Fig. 3B)	**9**
–	Body whorl very rounded (Fig. 3B)	**10**
9	Shell with whitish subsutural band (Figs 10D, 25A, B) and lower spire; flagellum short ca. half of epiphallus (Fig. 26A)	***S. lactospira* sp. nov.**
–	Shell monochrome brownish (without subsutural band) and higher spire; flagellum long ca. same length as penis and epiphallus (Fig. 26C)	***S. megalogyne* sp. nov.**
10	Head filament of spermatophore with obtuse-serrate longitudinal ridges (Fig. 16B)	***S. obesior***
–	Head filament of spermatophore with smooth longitudinal ridges (Fig. 4A)	**11**
11	Shell with higher spire; shorter vagina and free oviduct (Fig. 22C); spine II and spine III on tail filament of spermatophore start branching near the tip (Fig. 24C)	***S. caligina* sp. nov.**
–	Shell with lower spire; longer vagina and free oviduct (Fig. 28A); spine II and spine III on tail filament of spermatophore start branching near the base (Fig. 29C)	***S. subheptagyra* sp. nov.**
12	Tail filament of spermatophore near sperm sac with two spines (Fig. 4B)	***S. dohrniana***
–	Tail filament of spermatophore near sperm sac with three spines (Fig. 4B)	**13**
13	Penial retractor muscle very large and thickened (Fig. 12A, C)	***S. resplendens***
–	Penial retractor muscle very thin (Fig. 18C)	***S. heptagyra***
14	Body whorl obtuse angular (Fig. 3B)	**15**
–	Body whorl well rounded or shouldered (Fig. 3B)	**16**
15	Head filament of spermatophore with acute-serrate longitudinal ridges (Fig. 37B)	***S. bocourti***
–	Head filament of spermatophore with irregular-serrate longitudinal ridges and numerous porous (Fig. 36B)	***S. hainesi***
16	Body whorl strongly shouldered (Fig. 3B)	***S. inferospira* sp. nov.**
–	Body whorl well rounded or weak shouldered (Fig. 3B)	**17**
17	Snail has conspicuous dark spiral band at body whorl below suture (Fig. 33E); flagellum much smaller than epiphallus (Fig. 39C)	***S. melanospira* sp. nov.**
–	Snail monochrome colour (without spiral band at body whorl; Fig. 33F); flagellum slightly smaller than epiphallus (Fig. 41A)	***S. pellosa* sp. nov.**

#### Group I: *Sarika resplendens* group: species with left shell lobe and without penial verge and pseudo-verge

##### Sarika resplendens

Taxon classificationAnimaliaStylommatophoraAriophantidae

(Philippi, 1846)

0CB14588-C30B-56B8-B369-D1B2F9F30367

**Figure 5.**
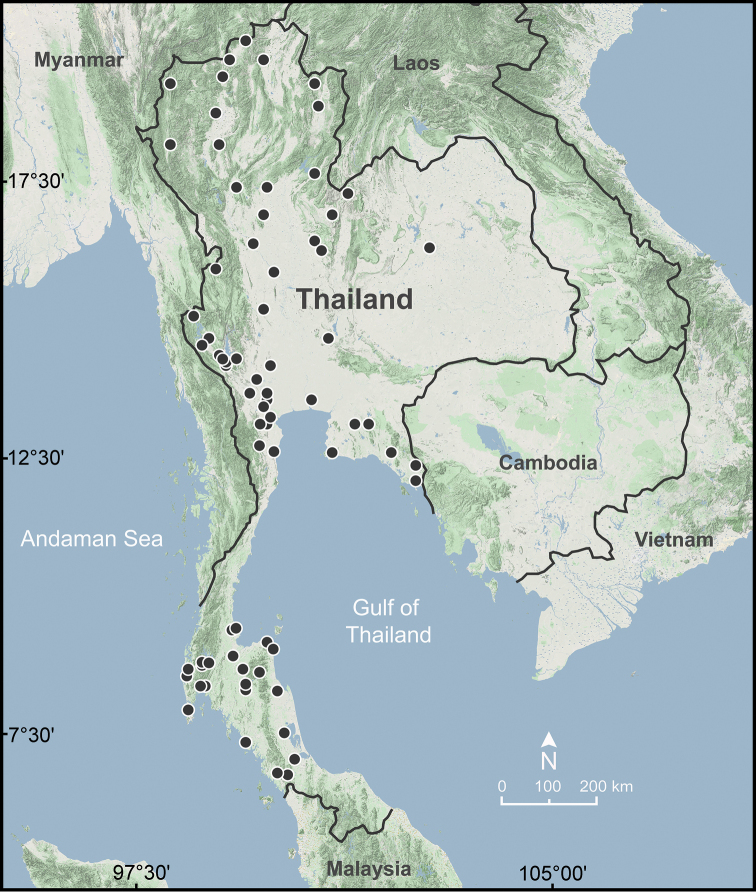
Geographic distribution of *Sarika resplendens* based on the specimens examined herein.

Figs 1 , 5 , 9A–C , 11A, B , 12 , 13 , 30A

Helix resplendens Philippi 1846: 192. Type locality: “Prope Mergui Indiae Orientalis” [Mergui Islands, Myeik District, Tanintharyi Region, Myanmar]. Pfeiffer 1848: 56. Pfeiffer 1849: 227, 228, pl. 110, figs 7–9. Pfeiffer 1868: 100. Hanley and Theobald 1872: 24, pl. 51, fig. 4. Hanley and Theobald 1876: 59, pl. 149, figs 2, 3.
Macrochlamys resplendens : Godwin-Austen 1883: 109, 110, pl. 26, figs 1–3. Godwin-Austen 1898: 49, 50. Collinge 1903: 209. Panha 1996: 34.
Nanina (Macrochlamys) resplendens : Tryon 1886: 91, pl. 30, figs 73–76. Fischer and Dautzenberg 1904: 395.
Ariophanta (Macrochlamys) resplendens : Fischer 1891: 21.
Sarika resplendens : Godwin-Austen 1907: 179–181, pl. 111, figs 3, 3a, pl. 116, figs 2, 2a, 2b. Blanford and Godwin-Austen 1908: 277, 278, fig. 84. Tomlin 1932: 316. Maassen 2001: 113. Hemmen and Hemmen 2001: 45. Sutcharit and Panha 2008: 96. Schileyko 2011: 34. Abu-Bakar et al. 2014. Inkhavilay et al. 2019: 82, figs 39a, 57a. Pholyotha et al. 2020c: 14, 15, figs 9a, b. Pholyotha et al. 2020a: 5, 6, fig. 2a.

###### Type material.

The type specimens of this species could not be located in the sizable part of Philippi’s collection in Museum für Naturkunde (Berlin), SMF, and NHM (Dance 1986; Coan and Kabat 2017). Coan and Kabat (2017) also stated that other parts of Philippi’s collection were presumably lost during World War II.

###### Other material examined.

**Myanmar.** Mergui NHMUK ex. Theobald collection: 1898.5.18.157 (13 specimens preserved in ethanol; Fig. 11A). Mergui, Tenasserim: NHMUK 1903.7.1.111 (specimen figured in Godwin-Austen 1883: pl. 26, Fig. 1). **Cambodia.** Cambodia: NHMUK 1903.7.1.112 (specimen figured in Godwin-Austen 1883: pl. 26, Fig. 2). **Thailand.** Siam: NHMUK 1903.7.1.113 (specimen figured in Godwin-Austen 1883: pl. 26, Fig. 3). **Thailand-Northeastern.** Area in Khok Ngam, Dan Sai, Loei, 17°21'12.5"N, 101°14'36.0"E: CUMZ 7873. Area in Sila, Mueang, Khon Kaen, 16°27'36.4"N, 102°49'09.4"E: CUMZ 7899. **Thailand-Eastern.** Bang Krachao, Phra Pradaeng, Samut Prakan, 13°41'16.7"N, 100°33'26.1"E: CUMZ 7880. Phothisat Kuan Im Shrine, Makham, Chanthaburi, 12°43'38.9"N, 102°08'06.8"E: CUMZ 7848. Wat Khao Sam Pong, Makham, Chanthaburi, 12°44'20.3"N, 102°06'57.1"E: CUMZ 7881. Khao Chi Chan, Sattahip, Chon Buri, 12°45'53.2"N, 100°57'24.3"E: CUMZ 7844. Wat Tham Khao Cha Ang On, Bo Thong, Chon Buri, 13°12'32.5"N, 101°39'05.7"E: CUMZ 7862. Mountain area near Ang Kep Nam Dan Chumphon, Bo Rai, Trat, 12°27'20.2"N, 102°39'59.3"E: CUMZ 7875. Saphan Hin Waterfall, Mueang, Trat, 12°06'07.8"N, 102°42'39.3"E: CUMZ 7887. **Thailand-Western.** Area near Srinakarin Dam, Si Sawat, Kanchanaburi, 14°23'55.3"N, 99°07'27.3"E: CUMZ 7843. Wat Tham Khao Cha Ang, Dan Makham Tia, Kanchanaburi, 13°48'05.0"N, 99°26'33.9"E: CUMZ 7850. Area in Tha Kha-nun, Thong Pha Phum, Kanchanaburi, 14°44'11.6"N, 98°38'20.7"E: CUMZ 7851. Wat Tha Khanun, Thong Pha Phum, Kanchanaburi, 14°44'30.6"N, 98°38'13.9"E: CUMZ 7864, 7886. Area near Wat Pak Lam Pilok, Thong Pha Phum, Kanchanaburi, 14°37'46.1"N, 98°34'30.3"E: CUMZ 7872. Area near Puprai Tarnnarm Resort, Thong Pha Phum, Kanchanaburi, 14°44'16.3"N, 98°38'37.3"E: CUMZ 7889. Wat Tham Mangkornthong, Mueang, Kanchanaburi, 13°59'10.0"N, 99°30'57.0"E: CUMZ 7852. Area in Ban Pu Toei, Sai Yok, Kanchanaburi, 14°19'36.4"N, 98°58'57.4"E: CUMZ 7863. Lawa Cave, Sai Yok, Kanchanaburi, 14°17'58.7"N, 98°58'54.8"E: CUMZ 7870. Area near Khao Wang Khamen, Sai Yok, Kanchanaburi, 14°22'33.1"N, 98°53'50.1"E: CUMZ 7884. Area near Hellfire Pass, Sai Yok, Kanchanaburi, 14°21'26.2"N, 98°57'02.6"E: CUMZ 7888. Wat Tham Faet, Tha Muang, Kanchanaburi, 13°57'49.3"N, 99°35'01.1"E: CUMZ 7865. Ban Song Karia, Sangkhla Buri, Kanchanaburi, 15°14'46.6"N, 98°25'32.9"E: CUMZ 7235. Wat Tham Sarika, Photharam, Ratchaburi, 13°38'45.8"N, 99°44'11.3"E: CUMZ 7846. Khao Changum, Photharam, Ratchaburi, 13°43'38.2"N, 99°44'34.3"E: CUMZ 7234. Wat Khao Phra, Photharam, Ratchaburi, 13°44'29.8"N, 99°44'44.1"E: CUMZ 7866. Wat Tham Nam, Photharam, Ratchaburi, 13°41'53.7"N, 99°45'24.0"E: CUMZ 7882. Wat Buri Ratchawanaram, Pak Tho, Ratchaburi, 13°22'45.0"N, 99°47'07.5"E: CUMZ 7867. Wat Khao Ban Dai, Nong Ya Plong, Phetchaburi, 13°14'08.5"N, 99°41'19.6"E: CUMZ 7847. Wat Puang Malai, Khao Yoi, Phetchaburi, 13°18'45.4"N, 99°47'01.7"E: CUMZ 7868, 7883. Khao Nok Wua Priest’s camp site, Cha-am, Phetchaburi, 12°44'46.9"N, 99°54'12.0"E: CUMZ 7891. Area near Ob Pha resort, Kaeng Krachan, Phetchaburi, 12°53'56.4"N, 99°38'53.8"E: CUMZ 7892. **Thailand-Central.** Wat Suwankuha (Ariyasatthi Cave), Phatthana Nikhom, Lopburi, 14°47'58.3"N, 100°53'10.0"E: CUMZ 7896. Wat Khao Bang Kraek, Nong Chang, Uthai Thani, 15°18'09.2"N, 99°41'03.7"E: CUMZ 7856. Wat Khao Tham Sua, U Thong, Suphan Buri, 14°22'19.1"N, 99°52'14.4"E: CUMZ 7859. Area near Kamphaengphet Historical Park, Mueang, Kamphaeng Phet, 16°30'27.6"N, 99°31'02.1"E: CUMZ 7860. Wat Thep Sathaporn, Banphot Phisai, Nakhon Sawan, 15°54'48.2"N, 99°53'02.6"E: CUMZ 7855. Limestone outcrop in Umphang, Umphang, Tak, 16°02'42.0"N, 98°48'59.4"E: CUMZ 7898. **Thailand-Northern.** Tham Luang Pha Wiang, Ban Hong, Lamphun, 18°13'19.1"N, 98°51'28.4"E: CUMZ 7861. Wat Tham Pha Ngam, Mae Phrik, Lampang, 17°28'49.2"N, 99°10'05.3"E: CUMZ 7857. Area near Pingkhong Resort, Chiang Dao, Chiang Mai, 19°28'02.6"N, 98°59'07.7"E: CUMZ 7876. Area near Khun Mai Baan Suan Resort, Mae Ai, Chiang Mai, 20°03'45.8"N, 99°21'33.0"E: CUMZ 7890. Wat Tham Pha Phueng, Chai Prakan, Chiang Mai, 19°44'18.7"N, 99°05'17.2"E: CUMZ 7845. Area in Hang Dong, Chiang Mai, 18°41'17.8"N, 98°54'10.8"E: CUMZ 7895. Si Satchanalai Historical Park, Si Satchanalai, Sukhothai, 17°25'34.8"N, 99°47'20.5"E: CUMZ 7879. Wat Khao Phra Noi, Mueang, Sukhothai, 17°01'07.9"N, 99°40'17.7"E: CUMZ 7894. Limestone outcrop near Golden Pai Resort, Mueang, Mae Hong Son, 19°21'32.0"N, 97°57'40.4"E: CUMZ 7858. Area in Mae Sariang Highway Division, Mae Sariang Mae Hong Son, 18°12'39.4"N, 97°56'14.1"E: CUMZ 7878. Area in Na Rai Luang, Song Khwae, Nan, 19°19'29.6"N, 100°41'14.1"E: CUMZ 7869. Tham Pha Sing, Song Khwae, Nan, 18°53'01.7"N, 100°45'03.1"E: CUMZ 7877. Wat Amarin Khuha (Wat Khao Tham Muang), Noen Maprang, Phitsanulok, 16°30'03.2"N, 100°41'16.4"E: CUMZ 7853. Tham Tao, Noen Maprang, Phitsanulok, 16°30'31.4"N, 100°39'44.4"E: CUMZ 7874, 7893. Area in Nakhon Thai, Phitsanulok, 17°08'56.0"N, 100°51'44.3"E: CUMZ 7897, Limestone outcrop near Mae Nam Pat, Nam Pat, Uttaradit, 17°44'26.3"N, 100°40'45.4"E: CUMZ 7854. Wat Tham Pha Phung, Wang Pong, Phetchabun, 19°24'09.5"N, 98°55'08.3"E: CUMZ 7871. **Thailand-Southern.**Wat Tham Sila Tiap, Tha Chana, Surat Thani, 9°30'58.9"N, 99°11'30.1"E: CUMZ 7832. Wat Tham Yai, Tha Chana, Surat Thani, 9°32'20.4"N, 99°11'26.1"E: CUMZ 7841. Wat Nakhawat, Phunphin, Surat Thani, 9°04'33.2"N, 99°09'54.0"E: CUMZ 7834. Wat Tham Wararam, Phanom, Surat Thani, 8°53'03.3"N, 98°40'02.5"E: CUMZ 7822. Limestone outcrop near Khao Sok Nature Resort, Phanom, Surat Thani 8°54'22.6"N, 98°31'45.1"E: CUMZ 7826. Wat Khiri Rat Phatthana, Wiang Sa, Surat Thani 8°31'38.6"N, 99°22'57.4"E: CUMZ 7833. Wat Na San, Na San, Surat Thani, 8°48'30.1"N, 99°22'10.4"E: CUMZ 7825. Area near Khanom Golden Beach, Khanom, Nakhon Si Thammarat, 9°10'58.6"N, 99°52'26.0"E: CUMZ 7815 (Fig. 11B), 7819. Wat Ao Sadet, Khanom, Nakhon Si Thammarat, 9°17'24.0"N, 99°47'18.2"E: CUMZ 7829. Area in Pak Phun, Mueang, Nakhon Si Thammarat, 8°27'44.1"N, 99°58'16.3"E: CUMZ 7840. Lot cave, Nopphitam, Nakhon Si Thammarat, 8°44'11.7"N, 99°38'07.3"E: CUMZ 7824. Wat Tham Thong Panara, Tham Phannara, Nakhon Si Thammarat, 8°25'19.8"N, 99°22'46.4"E: CUMZ 7835. Wat Tham Kanlayanamit, Tham Phannara, Nakhon Si Thammarat, 8°30'48.2"N, 99°22'50.7"E: CUMZ 7836. Hills near Hat Bang Sak, Takua Pa, Phang-nga, 8°46'31.0"N, 98°15'47.0"E: CUMZ 7817, 7818, 7830. Mountain area near Ban Pak Khlong, Kapong, Phang-nga, 8°50'21.2"N, 98°27'41.5"E: CUMZ 7821. Phung Chang Cave, Mueang, Phang-nga, 8°26'33.1"N, 98°30'55.0"E: CUMZ 7827. Mountain area near Khao Lak Resort, Takua Pa, Phang-nga, 8°38'23.8"N, 98°15'12.9"E: CUMZ 7831. Area near Mae Nam Phang-nga, Mueang, Phang-nga, 8°32'43.7"N, 98°28'21.2"E: CUMZ 7837. Tao Thong Waterfall, Thap Put, Phang-nga, 8°29'07.6"N, 98°35'08.5"E: CUMZ 7842. Area near Hat Chao Mai, Sikao, Trang, 7°24'48.9"N, 99°20'45.1"E: CUMZ 7839. Wat Kumphin Banpot, Khuan Kalong, Satun, 6°52'30.8"N, 100°01'02.4: CUMZ 7823. Limestone outcrop near Du Son, Khuan Don, Satun, 6°47'55.5"N, 100°06'43.4"E: CUMZ 7816. Wat Khuha Sawan, Mueang, Phatthalung, 7°37'14.1"N, 100°04'51.8"E: CUMZ 7838. Limestone outcrop in Sakhu, Thalang, Phuket, 8°05'25.1"N, 98°17'54.7"E: CUMZ 7828. Khao Jung Lone Cave, Rattaphum, Songkhla, 7°11'25.8"N, 100°16'59.9"E: CUMZ 7820.

###### Diagnosis.

Shell large, depressed and well-rounded body whorl. Animal with dark grey body and five mantle lobes. Genitalia with straight epiphallic caecum, large penial retractor muscle and small cuboidal penial pilasters. Spermatophore: head filament with irregularly obtuse-serrate longitudinal ridges; tail filament near sperm sac with three spines and terminal part more than ca. one-third of its length with series of several branching spines.

###### Description.

***Shell*.** Shell comparatively depressed, large size (shell width up to 23.4 mm, shell height up to 11.5 mm), and rather thin. Shell surface smooth and glossy; shell colour pale brown. Whorls 5½–6½, increasing regularly; body whorl large and well rounded. Spire slightly to moderately elevated; suture impressed. Aperture crescent-shaped and obliquely opened. Peristome simple. Columellar margin simple and little reflected near umbilicus. Umbilicus narrowly opened (Fig. 11A, B).

***Genital organs*.** Atrium short. Penis cylindrical with thin penial sheath covering proximal penis. Inner sculpture of penis fully covered with cuboidal penial pilasters of variable sizes; proximal area near atrium with very fine longitudinal pilasters then transformed to small pilasters; middle of chamber pilasters much larger than others; distal pilasters reduced to small pilasters. Epiphallus cylindrical, slightly narrower than penis and approximately as long as penis. Epiphallic caecum short, straight, same diameter as proximal epiphallus and located near middle of epiphallus. Penial retractor muscle large, thickened and attached at tip of epiphallic caecum. Flagellum long and slender tube, approximately half of epiphallus length. Vas deferens thin tube connecting distal epiphallus and free oviduct (Fig. 12).

Vagina cylindrical and short approximately one-third of penis length. Dart apparatus large, long cylindrical, and located on atrium at vagina and penis junction. Gametolytic sac bulbous (Fig. 12C with spermatophore); gametolytic duct long and cylindrical. Free oviduct cylindrical, longer than vagina and proximal end encircled with thick tissue (Fig. 12A, C).

Spermatophore long and needle-shaped. Sperm sac enlarged and elongate-oval. Head filament gourd shape with irregularly obtuse-serrate longitudinal ridges. Tail filament very long tube; region near sperm sac with three spines. Spine I located on same base with spine II, simple, short and little curved. Spine II large and long, branching into many spinules near the tip. Spine III shorter than spine II and with complicated branching into small and many spinules. Region furthest away smooth and without spine; terminal part (more than ca. one-third of its length) with series of short to long branching spines that arranged in a row or encircled tail filament tip (Fig. 13).

***Radula*.** Teeth arranged in a wide U-shape with half row formula: 1–(13–14)–70. Central tooth symmetrical tricuspid; mesocone large and triangular shape; ectocones very small. Lateral teeth asymmetrical tricuspid; mesocone pointed cusp, endocone and ectocone very small. Marginal teeth starting at approximately row number 13 or 14 with elongate bicuspid; endocone lanceolate shape; ectocone very small. Outermost teeth very short and smaller than inner teeth (Fig. 30A).

***External features*.** Animal with reticulated skin, dark grey body and dorsally with darker colour than below and foot sole. Caudal foss present; caudal horn raised and rather large. Mantle edge well developed, same colour as body, and with two shell lobes and three dorsal lobes. Shell lobes elongate; right shell lobe larger and longer than left shell lobe. Dorsal lobes large and broad; anterior left dorsal lobe and posterior left dorsal lobe (post-ldl) smaller than right dorsal lobe (Fig. 9A–C).

###### Distribution.

*Sarika resplendens* occurs throughout Thailand (Fig. 5) and mainland Southeast Asia (Schileyko 2011; Inkhavilay et al. 2019). This species can be found throughout the entire year in various humid areas in both natural and highly disturbed habitats. It is very easy to find and is the most common *Sarika* species in human-modified habitats such as plant farms or shaded gardens.

###### COI analysis.

The ML and BI analyses revealed that the individuals of *S. resplendens* (n = 7) form a monophyletic group with high support (Fig. 1; BS = 93%, PP = 1). The mean intraspecific genetic distance of *S. resplendens* was 1.8% (Table 2).

###### Remarks.

The type specimen of this species could not be located, only the specimens recognised by Godwin-Austen (1898, 1907) were examined. *Sarika resplendens* was examined in both shell and genitalia by Godwin Austen (1898, 1907), whose specimens were collected from Mergui [topotype] and sent by Theobald. In this study, we examined specimens from Mergui (Theobald collection) deposited in the NHM collections. Genitalia of these historical specimens and the new specimens from Thailand were identical in having a large penial retractor muscle and cuboidal shape of penial pilasters sculpture. In addition, the terminal part of the spermatophore tail filament had a cluster of branching spines. This character was not described or mentioned in Godwin Austen (1898, 1907).

##### Sarika dohrniana

Taxon classificationAnimaliaStylommatophoraAriophantidae

(Pfeiffer, 1860)

B4D94F08-7887-50F3-8FCD-B082820358BB

**Figure 6.**
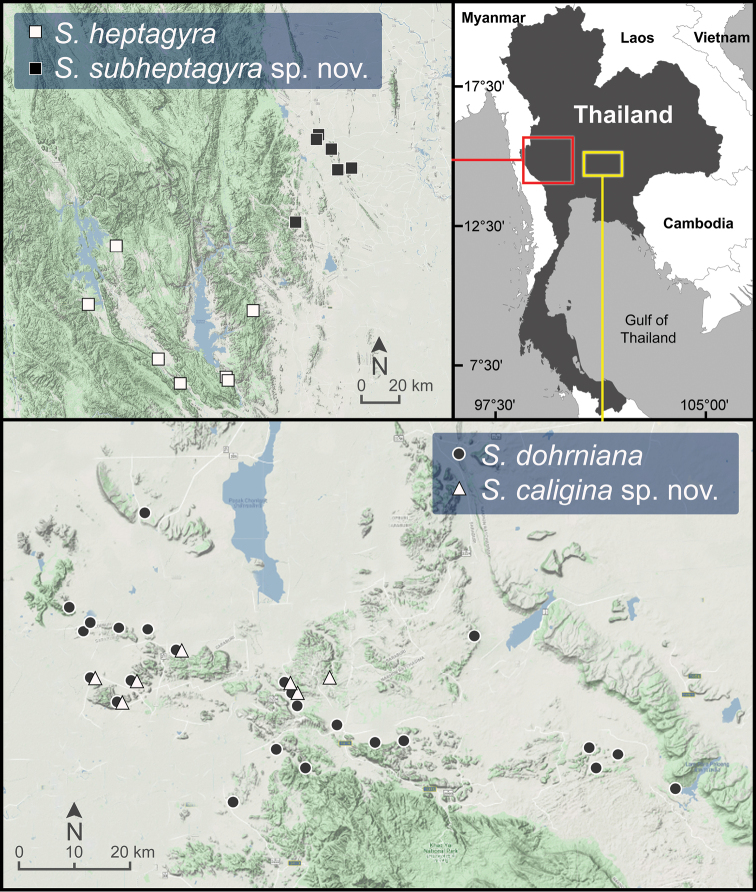
Geographic distribution of *Sarika caligina* sp. nov., *S. dohrniana*, *S. heptagyra*, and *S. subheptagyra* sp. nov. based on the specimens examined herein.

Figs 1 , 6 , 9D , 11C, D , 14A, B , 15 , 30B

Helix (Nanina) dohrniana Pfeiffer, 1860: 136. Type locality: “Siam” [Thailand]. Pfeiffer 1868: 97.
Nanina (Hemiplecta) dohrniana : Martens 1867: 72.
Nanina (Xestina) dohrniana : Tryon 1886: 83, pl. 16, figs 23, 24.
Nanina (Xesta) dohrniana : Fischer and Dautzenberg 1904: 394.
Ariophanta (Hemiplecta) dohrniana : Fischer 1891: 22.
Sarika dohrniana : Godwin-Austen 1907: 181, 182; Sutcharit and Panha 2008: 96.

###### Type material.

***Syntypes***NHMUK ex. Cuming collection: 20160046 (three shells; Fig. 11C) from Siam [Thailand].

###### Other material examined.

**Thailand.** Siam: NHMUK 1903.7.1.464 (one shell). **Thailand-Northeastern.** Dong Phya Fai, Siam: NHMUK 1903.7.1.1702 ex. Flower collection (two shells). Wat Thep Phithak Punnaram, Pak Chong, Nakhon Ratchasima, 14°36'57.3"N, 101°15'34.3"E: CUMZ 7616. Wat Tham Thian Chai Chonprathan, Pak Chong, Nakhon Ratchasima, 14°37'07.5"N, 101°18'15.5"E: CUMZ 7621. Wat Tham Praput, Pak Chong, Nakhon Ratchasima, 14°35'37.6"N, 101°40'16.8"E: CUMZ 7619. Wat Tham Pin Kaeo, Pak Chong, Nakhon Ratchasima, 14°36'26.4"N, 101°37'11.4"E: CUMZ 7622. Wat Tham Sap Muet, Pak Chong, Nakhon Ratchasima, 14°47'14.0"N, 101°25'49.8"E: CUMZ 7624. Wat Tham Santitham, Pak Chong, Nakhon Ratchasima, 14°34'29.7"N, 101°37'36.5"E: CUMZ 7625. Muak Lek Waterfall, Pak Chong, Nakhon Ratchasima, 14°38'36.1"N, 101°12'17.4"E: CUMZ 7632. Mountain area near Lam Phra Phloeng Dam, Pak Thong Chai, Nakhon Ratchasima, 14°32'33.0"N, 101°45'37.0"E: CUMZ 7631. **Thailand-Central.** Wat Tham Mongkut, Phra Phutthabat, Saraburi, 14°40'40.9"N, 100°50'33.6"E: CUMZ 7620. Tham Rakhang-Tham Kin Non, Phra Phutthabat, Saraburi, 14°42'57.1"N, 100°47'49.3"E: CUMZ 7626. Saeng Tham Cave, Muak Lek, Saraburi: CUMZ 7613. Wat Tham Tham Osot, Muak Lek, Saraburi, 14°42'35.7"N, 101°07'01.5"E: CUMZ 7627. Wat Tham Rattana Buppha, Muak Lek, Saraburi, 14°41'35.3"N, 101°07'50.5"E: CUMZ 7629. Wat Tham Si Wilai, Chaloem Phra Kiat, Saraburi, 14°42'44.4"N, 100°51'58.5"E: CUMZ 7630, 7633. Wat Tham Phra Phothisat, Kaeng Khoi, Saraburi, 14°34'33.2"N, 101°08'42.6"E: CUMZ 7614, 7618. Wat Tham Khao Kaeo, Kaeng Khoi, Saraburi, 14°36'16.9"N, 101°05'36.6"E: CUMZ 7628. Mountain area in Thap Kwang, Kaeng Khoi, Saraburi, 14°31'32.7"N, 101°01'37.4"E: CUMZ 7635. Wat Tham Mongkol Nimit, Mueang, Lopburi, 14°49'34.1"N, 100°45'28.4"E: CUMZ 7611 (Fig. 11D). Wat Pa Tham Sua, Mueang, Lopburi, 14°48'48.6"N, 100°47'03.9"E: CUMZ 7612. Wat Tham Phrathat, Mueang, Lopburi, 14°48'23.6"N, 100°49'29.7"E: CUMZ 7615. Wat Tham Muang, Mueang, Lopburi, 14°48'07.1"N, 100°46'40.3"E: CUMZ 7617. Wat Tham Phrom Sawat, Phatthana Nikhom, Lopburi, 14°45'32.0"N, 100°56'22.4"E: CUMZ 7623. Wat Pa Tham Ariyasatsi, Phatthana Nikhom, Lopburi, 14°47'58.3"N, 100°53'10.0"E: CUMZ 7636. Mountain area in Chai Badan, Chai Badan, Lopburi, 14°59'21.2"N, 100°52'34.3"E: CUMZ 7634.

###### Diagnosis.

Shell large to very large, depressed to conoid-depressed and rounded to slightly obtusely angulated body whorl. Animal with pale grey body and five mantle lobes. Genitalia with straight epiphallic caecum and small cuboidal penial pilasters. Spermatophore: head filament with irregularly smooth longitudinal ridges; tail filament near sperm sac with two spines and terminal part more than ca. one-eighth of its length with series of several branching spines.

###### Description.

***Shell*.** Shell depressed to conoid-depressed, large to very large size (shell width up to 33.2 mm, shell height up to 18.9 mm) and rather thin to slightly solid. Shell surface smooth, rather coarse above periphery; shell colour yellowish brown to dark brown. Whorls 6–6½, increasing regularly; body whorl large and rounded to slightly obtusely angulated. Spire very much elevated; suture impressed. Aperture crescent-shaped and obliquely opened. Peristome simple. Columellar margin simple and slightly reflected near umbilicus. Umbilicus narrowly opened (Fig. 11C, D).

***Genital organs*.** Atrium short. Penis cylindrical, elongate and with thin penial sheath covering proximal penis. Inner sculpture of penis proximally more than ca. half of penial chamber with very fine longitudinal penial pilasters, and then transformed to small cuboidal pilasters. Epiphallus cylindrical, as long as penis and slightly narrower than penis. Epiphallic caecum short, straight, approximately same diameter as epiphallus, and located near middle of epiphallus. Penial retractor muscle thin and attached at tip of epiphallic caecum. Flagellum slender and long, approximately as long as epiphallus. Vas deferens thin tube connecting distal epiphallus and free oviduct (Fig. 14A, B).

Vagina cylindrical and approximately half of penis length. Dart apparatus enlarged, long cylindrical, and located on atrium at vagina and penis junction. Gametolytic sac bulbous; gametolytic duct long and cylindrical. Free oviduct short, approximately half of vagina length, and proximal end encircled with thick tissue (Fig. 14A).

Spermatophore long and needle-shaped. Sperm sac enlarged and elongate-oval. Head filament gourd shape with irregularly smooth longitudinal ridges. Tail filament very long tube; region near sperm sac with two spines. Spine I simple and little curved. Spine II long and branching into many spinules near the tip. Most of region furthest away smooth and without spine; terminal part (more than ca. one-eighth of its length) with series of short to long branching spines arranged in opposite rows (Fig. 15).

***Radula*.** Teeth with half row formula: 1–(19–20)–80. Central tooth symmetrical tricuspid; lateral teeth asymmetrical tricuspid; marginal teeth elongate bicuspid. Marginal teeth starting at ca. row number 19 or 20 (Fig. 30B).

***External features*.** Animal with reticulated skin and pale grey body, dark creamy mixing with grey foot sole and dark creamy to pale grey caudal horn. Mantle edge well developed and same colour as body (Fig. 9D).

###### Distribution.

*Sarika dohrniana* occurs in the karstic habitats and forested mountains in northeastern and central Thailand (Fig. 6).

###### COI analysis.

The ML and BI analyses of *S. dohrniana* revealed that three individuals formed a monophyletic group with very strong support (Fig. 1; BS = 100%, PP = 1), sister to the rest of most species of *Sarika*, except for *S. solemi* sp. nov. + *S. dugasti*. The mean intraspecific genetic distance of *S. dohrniana* was 1.7% (Table 2).

###### Remarks.

The remarkable characters of *S. dohrniana* are its large size, conoid-depressed shell, the coarser shell surface compared to other *Sarika* species, and the head filament of the spermatophore with its irregularly smooth longitudinal ridges and the two spines on the tail filament near the sperm sac.

##### Sarika obesior

Taxon classificationAnimaliaStylommatophoraAriophantidae

(Martens, 1867)

8C90A0D9-5A2A-5543-B265-CA8E5F4CEC62

Figs 1 , 8 , 9E , 11E, F , 14C, D , 16 , 30C

**Figure 7.**
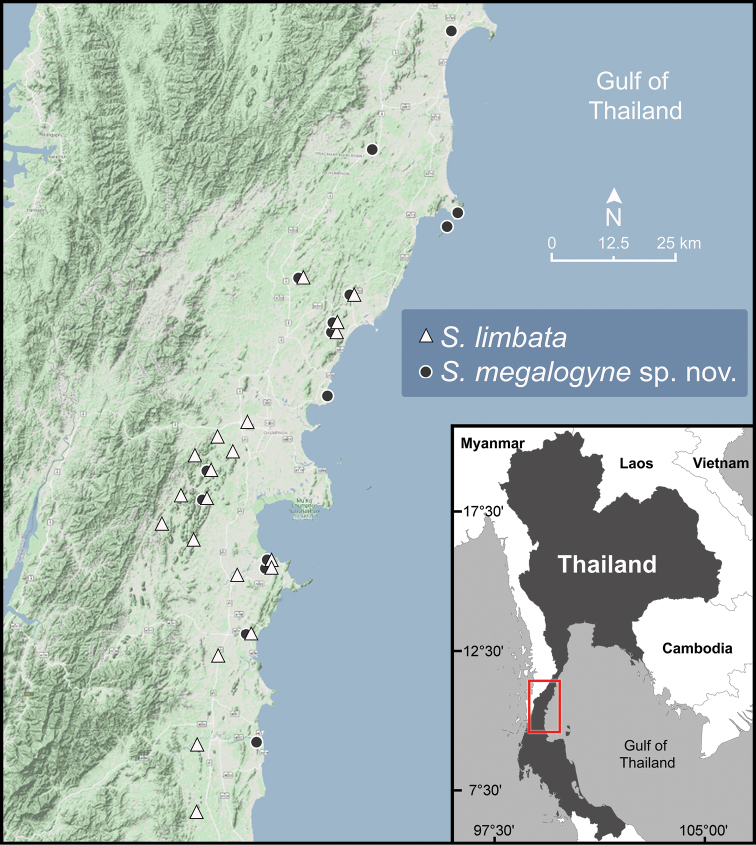
Geographic distribution of *Sarika limbata* and *S. megalogyne* sp. nov. based on the specimens examined herein.

Nanina (Orobia) resplendens var. obesior Martens 1867: 72, pl. 12, fig. 6. Type locality: “Um Petshaburi nicht selten; auch an der Ostseite des Golfs bei Bang-Pra beobachtet” [Petchaburi Province and Bang Phra, Si Racha District, Chonburi Province, Thailand].
Nanina (Macrochlamys) resplendens obesior : Tryon 1886: 91, pl. 30, fig. 76.
Sarika obesior : Solem 1966: 36–38, fig. 5b, c; Hemmen and Hemmen 2001: 45; Sutcharit and Panha 2008: 96; Pholyotha et al. 2020c: 18, 19, figs 2f, g, 7e, f, 10a, b, 13d–f.

###### Type material.

The type specimens could not be located and were probably missing from the Museum für Naturkunde (Berlin) collection (T. von Rintelen and C. Zorn, pers. comm., December 2018).

###### Other material examined.

**Myanmar.** Forest on Kala Island, Myeik District, Tanintharyi Division, 12°25'25.4"N, 98°29'50.6"E: CUMZ 7140, 7141. **Thailand-Western.** Limestone outcrop in Kaeng Krachan, Kaeng Krachan, Phetchaburi, 12°55'01.0"N, 99°37'48.0"E: CUMZ 7686. Wat Tham Rong, Ban Lat, Phetchaburi, 13°01'31.3"N, 99°55'07.9"E: CUMZ 7673 (Fig. 11F), 7693. Wat Khao Krachiu, Tha Yang, Phetchaburi, 12°57'42.9"N, 99°54'48.8"E: CUMZ 7670. Khao Tamo, Tha Yang, Phetchaburi, 12°47'49.0"N, 99°44'40.0"E: CUMZ 7682. Wat Khiriwong, Khao Yoi, Phetchaburi, 13°20'02.2"N, 99°45'19.9"E: CUMZ 7668. Wat Puang Malai, Khao Yoi, Phetchaburi, 13°18'40.4"N, 99°47'09.2"E: CUMZ 7675 (Fig. 11E). Khao Nang Panthurat, Cha-am, Phetchaburi, 12°50'22.2"N, 99°57'10.0"E: CUMZ 7680, 7681, 7694, 7695. Khao Na Kwang Cave Cha-am, Phetchaburi, 12°51'31.3"N, 99°56'29.3"E: CUMZ 7683, 7690. Wat Tham Jaeng, Cha-am, Phetchaburi, 12°49'39.3"N, 99°56'24.5"E: CUMZ 7667. Khao Ma Rong Cave, Bang Saphan, Prachuap Khiri Khan, 11°12'09.3"N, 99°29'39.8"E: CUMZ 7664, 7669, 7702. Wat Tham Siriwong, Bang Saphan, Prachuap Khiri Khan, 11°23'27.6"N, 99°34'55.3"E: CUMZ 7665, 7697, 7704. Wat Tham Khao Wong, Bang Saphan, Prachuap Khiri Khan, 11°17'28.0"N, 99°29'45.4"E: CUMZ 7666. Ko Thalu, Bang Saphan Noi, Prachuap Khiri Khan, 11°04'34.4"N, 99°33'37.4"E: CUMZ 7691. Khao Ta Mong Lai, Mueang, Prachuap Khiri Khan, 11°50'00.3"N, 99°49'47.2"E: CUMZ 7674. Khao Lom Muak, Mueang, Prachuap Khiri Khan, 11°47'08.5"N, 99°48'57.9"E: CUMZ 7676. Wat Ao Noi, Mueang, Prachuap Khiri Khan, 11°51'44.0"N, 99°49'20.0"E: CUMZ 7701, 7703. Tham Khao Rak Mai, Thap Sakae, Prachuap Khiri Khan, 11°25'42.1"N, 99°36'18.5"E: CUMZ 7677. Khao Kalok, Sam Roi Yot, Prachuap Khiri Khan, 12°20'13.0"N, 99°59'59.0"E: CUMZ 7678, 7698. Phraya Nakhon Cave, Sam Roi Yot, Prachuap Khiri Khan, 12°11'45.3"N, 100°00'41.8"E: CUMZ 7685. Limestone outcrop near Hat Sam Phraya, Kui Buri, Prachuap Khiri Khan, 12°09'04.0"N, 99°58'58.0"E: CUMZ 7705. Limestone outcrop in Kui Buri, Kui Buri, Prachuap Khiri Khan, 12°08'06.1"N, 99°38'41.2"E: CUMZ 7684, 7692, 7699. Wat Nong Phlap, Hua Hin, Prachuap Khiri Khan, 12°35'29.9"N, 99°43'45.8"E: CUMZ 7233. Pa La-U Waterfall, Hua Hin, Prachuap Khiri Khan, 12°32'17.2"N, 99°27'48.7"E: CUMZ 7700. Lublae Cave, Hua Hin, Prachuap Khiri Khan, 12°36'15.1"N, 99°43'20.9"E: CUMZ 7679. **Thailand-Southern.** Wat Bonphot Phisai, Lang Suan, Chumphon, 9°56'12.5"N, 99°08'44.2"E: CUMZ 7671. Limestone outcrop in Khlong Sok, Phanom, Surat Thani, 8°50'53.0"N, 98°44'35.3"E: CUMZ 7688. Limestone outcrop near Anurak Community Lodge, Phanom, Surat Thani, 8°53'16.3"N, 98°40'45.2"E: CUMZ 7689. Wat Tham Wararam, Phanom, Surat Thani: CUMZ 7696. Khao Kloi, Don Sak, Surat Thani, 8°52'43.9"N, 98°39'26.5"E: CUMZ 7672.

###### Diagnosis.

Shell medium to large, depressed and well rounded body whorl. Animal with greyish to slightly dark grey body and five mantle lobes. Genitalia with straight epiphallic caecum and triangular prism penial pilasters. Spermatophore: head filament with irregularly obtuse-serrate longitudinal ridges; tail filament near sperm sac with three spines and terminal part more than ca. one-third of its length with series of several branching spines.

###### Description.

The unique shell characters of *S. obesior* are depressed, medium to large size (shell width up to 22.1 mm; shell height up to 11.3 mm), pale brown, well-rounded body whorl, spire elevated, and impressed suture (Fig. 11E, F).

The unique genitalia characters are straight epiphallic caecum; inner wall of penis with very fine longitudinal penial pilasters near atrium, changing to large rhomboid pilasters with acute angle on top (triangular prism shape) (Fig. 14C, D).

Spermatophore long and needle-shaped. Sperm sac (ss) enlarged and elliptical. Head filament gourd shape with irregularly obtuse-serrate longitudinal ridges. Tail filament very long tube; region near sperm sac with three spines. Spine I located near base of spine II, simple and short. Spine II broken. Spine III large with complicated branching into small and many spinules. Region furthest away smooth and without spine; terminal part (more than ca. one-third of its length) with series of short to long branching spines arranged in a row or encircling the tail filament tip (Fig. 16).

Radula with half row formula: 1–(13–14)–60. The morphology of central tooth, lateral, and marginal teeth are similar to that described in Pholyotha et al. (2020c: figs 13d–f). Marginal teeth starting at approximately row number 13 or 14 (Fig. 30C).

Living snail with monochrome greyish to slightly dark grey body. Mantle edge well developed and same colour same body (Fig. 9E).

###### Distribution.

**Figure 8.**
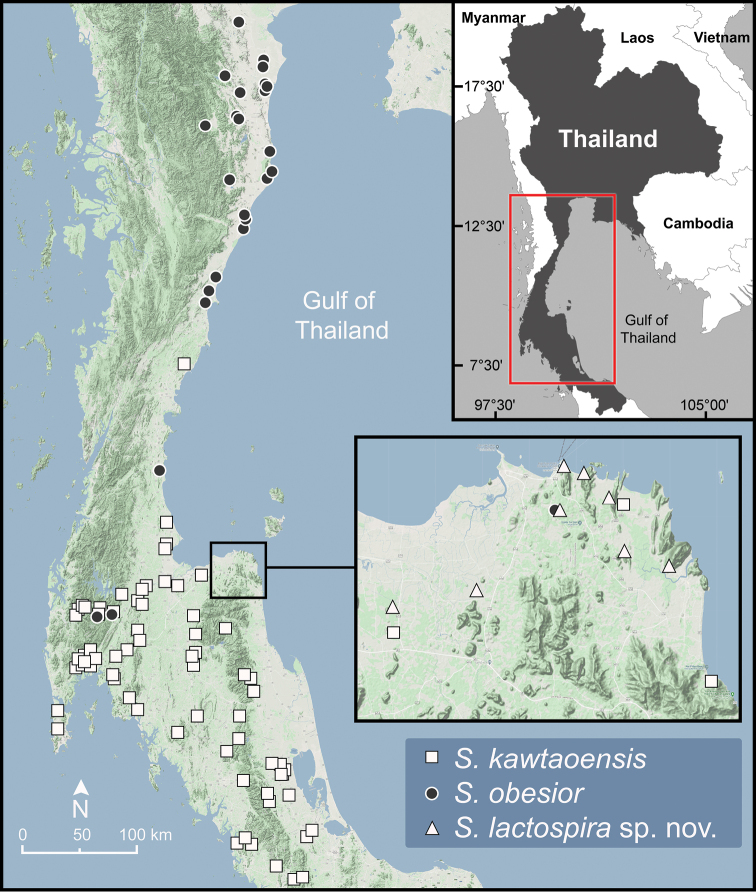
Geographic distribution of *Sarika kawtaoensis*, *S. lactospira* sp. nov., and *S. obesior* based on the specimens examined herein.

*Sarika obesior* occurs in western and southern Thailand (Fig. 8) and Tanintharyi region in Myanmar (Pholyotha et al. 2020c).

###### COI analysis.

The ML and BI analyses showed that the individuals of *S. obesior* (n = 4) formed a monophyletic group with high support (Fig. 1; BS = 98%, PP = 1). The mean intraspecific genetic distance of *S. obesior* was 3.0% (Table 2).

###### Remarks.

This species has recently been re-described and illustrated based on the samples collected from Myeik, Myanmar by Pholyotha et al. (2020c). The topotypic specimens from Petchaburi, Thailand agree well with the previous descriptions. Solem (1966) noted that *S. obesior* differs from *S. hainesi* in having a rounded body whorl and attributed several specimens from northern and eastern Thailand to *S. obesior*. Yet, without the genital anatomy their identification could not be confirmed. Therefore, the specimens from northern and eastern Thailand as reported by Solem (1966) are still doubtful, and to date, the distribution of *S. obesior* is probably restricted to western and southern Thailand and south-eastern Myanmar.

##### Sarika limbata

Taxon classificationAnimaliaStylommatophoraAriophantidae

(Möllendorff, 1894)

1E5BDBFC-95A0-5D1D-8B2D-2BEBE547F5F5

**Figure 9.**
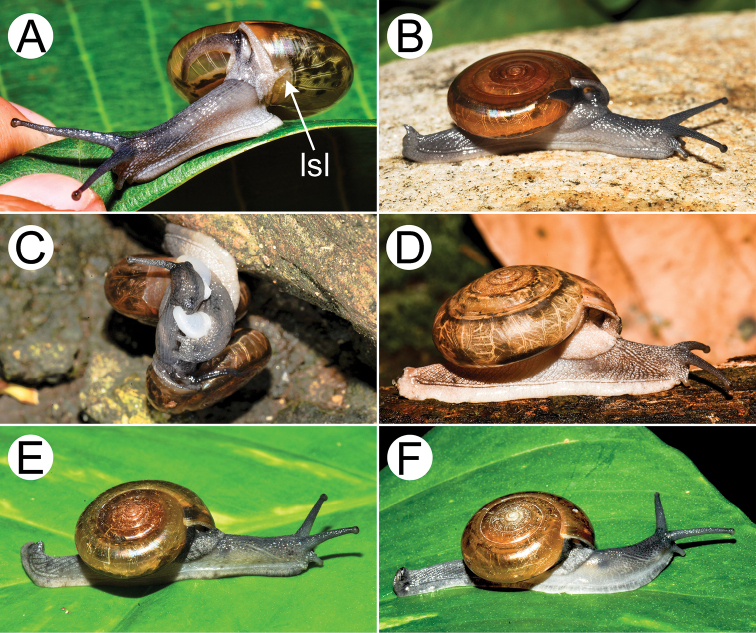
Living snails of group I: *Sarika resplendens* group. **A***Sarika resplendens* specimen CUMZ 7843 showing left shell lobe lobes (lsl) **B***S. resplendens* specimen CUMZ 7827 **C** mating pairs of *S. resplendens*CUMZ 7876 **D***S. dohrniana* specimen CUMZ 7611 **E***S. obesior* specimen CUMZ 7673 **F***S. limbata* specimen CUMZ 7652. All not to scale.

Figs 1 , 7 , 9F , 17A–C , 18A, B , 19 , 30D

Macrochlamys limbata Möllendorff 1894: 148, pl. 16, figs 6, 7. Type locality: “Samui Islands, Gulf of Siam” [Samui Islands, Ko Samui District, Surat Thani Province, Thailand]; Panha 1996: 34; Hemmen and Hemmen 2001: 44.
Nanina (Macrochlamys) limbata : Fischer and Dautzenberg 1904: 395.
Sarika limbata : Tomlin 1929: 16.

###### Type material.

***Syntypes***SMF 227100 (Fig. 17A) from Insel Samui, Gulf der Siam [Samui Island, Surat Thani, Thailand], SMF 90854/2 (two shells), 90855/4 (four shells), 227101/3 (three shells; Fig. 17B), 227102/1 (one shell).

###### Other material examined.

**Thailand-Southern.** Insel Samui, Gulf der Siam [Samui Island, Gulf of Thailand], 9°28'02.9"N, 99°58'43.8"E: SMF 298596/2. Tham Chang Phuek Bureau of Monks, Mueang, Chumphon, 10°26'47.0"N, 99°02'06.0"E: CUMZ 7637, 7647, 7654. Tham Krating Thong Bureau of Monks, Mueang, Chumphon, 10°27'20.6"N, 99°06'20.6"E: CUMZ 7638. Tham Sing, Mueang, Chumphon, 10°25'45.7"N, 99°02'52.7"E: CUMZ 7640. Wat Tham Sanook, Mueang, Chumphon, 10°28'52.3"N, 99°04'31.3"E: CUMZ 7644, 7646. Wat Uthaitham, Mueang, Chumphon, 10°30'24.1"N, 99°07'50.4"E: CUMZ 7658. Pla Cave, Thung Tako, Chumphon, 10°07'58.3"N, 99°08'08.1"E: CUMZ 7639. Wat Phut Sadi Phupharam, Thung Tako, Chumphon, 10°05'37.0"N, 99°04'39.3"E: CUMZ 7652 (Fig. 17C). Tham Pisadan Bureau of Monks, Tha Sae, Chumphon, 10°45'36.7"N, 99°13'45.8"E: CUMZ 7655, 7657. Nang Thong Cave, Pathio, Chumphon, 10°40'14.3"N, 99°17'35.9"E: CUMZ 7641. Wat Tham Khao Plu, Pathio, Chumphon, 10°43'49.6"N, 99°19'19.7"E: CUMZ 7660. Wat Tham Khao Bang Siap, Pathio, Chumphon, 10°40'06.7"N, 99°17'37.9"E: CUMZ 7661. Wat Rat Burana, Lang Suan, Chumphon, 9°56'20.6"N, 99°02'25.7"E: CUMZ 7645. Tham Khao Kriap, Lang Suan, Chumphon, 9°49'03.0"N, 99°02'18.0"E: CUMZ 7649, 7659, 7662, 7663. Tham Khao Lak Bureau of Monks, Sawi, Chumphon, 10°19'39.7"N, 98°58'39.3"E: CUMZ 7642. Tham Nam Lod Thepnimit Bureau of Monks, Sawi, Chumphon, 10°22'36.9"N, 99°00'42.2"E: CUMZ 7643. Cholkhiri Bureau of Monks, Sawi, Chumphon, 10°22'20.1"N, 99°03'29.7"E: CUMZ 7648. Wat Nam Cha, Sawi, Chumphon, 10°17'50.9"N, 99°01'57.4"E: CUMZ 7650. Limestone outcrop in Sawi, Sawi, Chumphon, 10°15'05.0"N, 99°10'25.6"E: CUMZ 7651. Wat Tham Khao Lan, Sawi, Chumphon, 10°15'47.7"N, 99°10'18.1"E: CUMZ 7653. Wat Tham Khwan Mueang, Sawi, Chumphon, 10°14'18.2"N, 99°06'43.3"E: CUMZ 7656.

###### Diagnosis.

Shell large, depressed and well-rounded body whorl. Animal with pale to dark grey body and five mantle lobes. Genitalia with straight epiphallic caecum and inner penial sculpture with small reticulated pilasters in proximal part and small cuboidal pilasters in distal end. Spermatophore: head filament with irregularly plate-like sculpture; tail filament near sperm sac with three spines and terminal part more than ca. one-third of its length with series of several branching spines.

###### Description.

***Shell*.** Shell depressed, large size (shell width up to 27.0 mm, shell height up to 13.7 mm) and rather thin. Shell surface smooth, shiny; shell colour pale yellowish brown to pale brown. Whorls 6–6½, increasing regularly; body whorl large and well-rounded. Spire elevated; suture impressed. Aperture crescent-shaped and obliquely opened. Peristome simple. Columellar margin simple and slightly reflected near umbilicus. Umbilicus narrowly opened (Fig. 17A–C).

***Genital organs*.** Atrium short. Penis cylindrical with thin penial sheath covering proximal penis. Inner sculpture of penis proximally more than ca. one-fifth of penial chamber covered with very fine longitudinal penial pilasters, changing to small and thin reticulated pilasters around two-fifth of chamber, and transformed to small cuboidal pilasters at distal end near epiphallus. Epiphallus cylindrical, approximately two times total penis length, and smaller diameter than penis. Epiphallic caecum short, straight, similar diameter as proximal epiphallus, and located near middle of epiphallus. Penial retractor muscle thin and attached at tip of epiphallic caecum. Flagellum very slender, and approximately same length as penis. Vas deferens thin tube connecting distal epiphallus and free oviduct (Fig. 18A, B).

Vagina cylindrical and ca. two-thirds penis length. Dart apparatus large, long, cylindrical, and located on atrium of vagina and penis junction. Gametolytic sac enlarged and bulbous; gametolytic duct long and cylindrical. Free oviduct cylindrical, approximately same length with penis, and proximal end encircled with thick tissue (Fig. 18A).

Spermatophore long and needle-shaped. Sperm sac enlarged and elongate-oval. Head filament gourd shape; region close to sperm sac with irregularly plate-like sculpture then transformed to irregularly acute-serrate longitudinal ridges. Tail filament very long tube; region near sperm sac with three spines. Spine I very reduced to small knob. Spine II was broken but slightly large at base. Spine III (partially broken) with branching into small spines and spinules. Region furthest away smooth and without spine; terminal part (more than ca. one-third of its length) with series of short to long and complicated branching spines, arranged in a row or opposite rows near tail filament tip (Fig. 19).

***Radula*.** Teeth with half row formula: 1–(16–17)–69. Central tooth symmetrical tricuspid; lateral teeth asymmetrical tricuspid; marginal teeth elongate bicuspid. Marginal teeth starting at approximately row number 16 or 17 (Fig. 30D).

***External features*.** Animal with reticulated skin and pale to dark grey body, pale grey foot sole, and dark grey caudal horn. Mantle edge well developed and same colour as body (Fig. 9F).

###### Distribution.

*Sarika limbata* occurs eastwards of the Tenasserim Range to the Phuket Range and is common in Chumphon Province (Fig. 7).

###### COI analysis.

The ML and BI analyses revealed that the samples of *S. limbata* (n = 4) formed a monophyletic group with very strong support (Fig. 1; BS = 100%, PP = 1), and sister to *S. lactospira* sp. nov. with only BI support (Fig. 1; PP = 0.99). The mean intraspecific genetic distance of *S. limbata* was 1.2% (Table 2).

*Sarika limbata*, *S. kawtaoensis*, and *S. lactospira* sp. nov. are phylogenetically closely related, yet only with BI support (Fig. 1). The average interspecific sequence divergences were 7.2% (*S. limbata* and *S. kawtaoensis*), 7.5% (*S. limbata* and *S. lactospira* sp. nov.), and 6.9% (*S. kawtaoensis* and *S. lactospira* sp. nov.) (Table 2).

###### Remarks.

The shell morphology of *S. limbata* is similar to that of *S. kawtaoensis*. The main distinguishing characters of *S. limbata* are the reticulated and cuboidal penial pilasters, and the plate-like and acute-serrate longitudinal ridges on the head filament of the spermatophore. In contrast, *S. kawtaoensis* has reticulated and irregular large folded penial pilasters and acute-serrate longitudinal ridges on the head filament of the spermatophore.

##### Sarika heptagyra

Taxon classificationAnimaliaStylommatophoraAriophantidae

(Möllendorff, 1902)

8B432B41-C9A8-555F-A643-9D4811579F39

**Figure 10.**
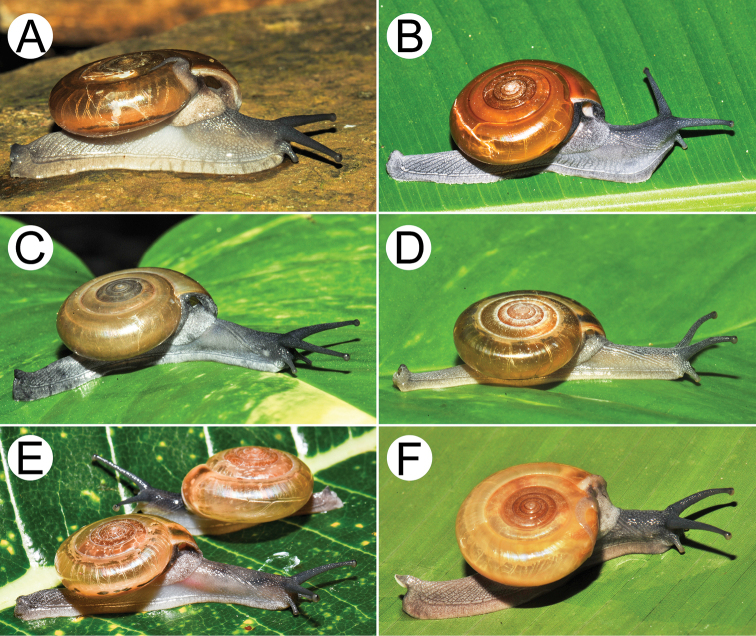
Living snails of group I: *Sarika resplendens* group. **A***Sarika heptagyra* specimen CUMZ 7279 **B***S. kawtaoensis* specimen CUMZ 7738 **C***S. caligina* sp. nov. paratype CUMZ 7245 **D***S. lactospira* sp. nov. paratype CUMZ 7287 **E***S. megalogyne* sp. nov. paratype CUMZ 7522 and **F***S. subheptagyra* sp. nov. paratype CUMZ 7507. All not to scale.

Figs 1 , 6 , 10A , 17D–F , 18C, D , 20 , 30E

Macrochlamys heptagyra Möllendorff 1902: 155. Type locality: “Kanburi” [Kanchanaburi Province, Thailand].
Nanina (Macrochlamys) heptagyra : Fischer and Dautzenberg 1904: 395.

###### Type material.

***Syntypes***SMF 227096 (Fig. 17D), SMF 227097/3 (three shells; Fig. 17E) from Siam: Kanburi [Kanchanaburi, Thailand].

###### Other material examined.

**Thailand-Western.** Wat Dao Wadung, Sai Yok, Kanchanaburi, 14°28'23.3"N, 98°50'04.7"E: CUMZ 7232, 7280, 7285. Limestone outcrop in Sai Yok, Sai Yok, Kanchanaburi, 14°22'46.0"N, 98°55'50.0"E: CUMZ 7282. Limestone outcrop in Khao Chot, Si Sawat, Kanchanaburi, 14°39'41.7"N, 99°17'09.6"E: CUMZ 7281. Erawan waterfall, Si Sawat, Kanchanaburi, 14°22'07.1"N, 99°08'38.3"E: CUMZ 7283. Limestone outcrop in Tha Kradan, Si Sawat, Kanchanaburi, 14°22'31.8"N, 99°08'38.3"E: CUMZ 7284. Kroeng Krawia, Thong Pha Phum, Kanchanaburi, 14°56'24.7"N, 98°39'47.8"E: CUMZ 7279. Wat Uthum Phon Wanaram (Tham Khao Noi), Thong Pha Phum, Kanchanaburi, 14°41'52.1"N, 98°31'32.7"E: CUMZ 7231 (Fig. 17F).

###### Diagnosis.

Shell large, strongly depressed and well-rounded to slightly shouldered body whorl. Animal with pale grey body and five mantle lobes. Genitalia with straight epiphallic caecum and cuboidal penial pilasters. Tail filament of spermatophore near sperm sac with three spines and terminal part of tail filament more than ca. one-fourth of its length with series of several branching spines.

**Figure 11.**
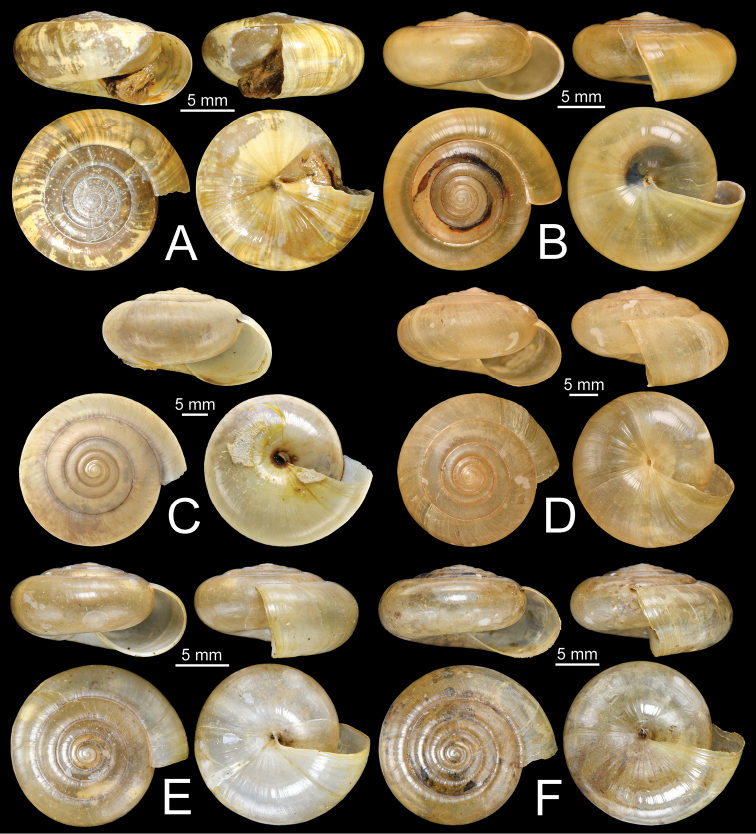
Shells of group I: *Sarika resplendens* group. **A, B***Sarika resplendens***A** specimen NHMUK 1898.5.18.157 and **B** specimen CUMZ 7815 **C, D***S. dohrniana***C** syntype NHMUK 20160046 and **D** specimen CUMZ 7611. **E, F***S. obesior***E** specimen CUMZ 7675 and **F** specimen CUMZ 7673.

###### Description.

***Shell*.** Shell strongly depressed, large size (shell width up to 27.9 mm, shell height up to 13.1 mm) and rather thin. Shell surface smooth and glossy; shell colour pale yellowish brown to very pale brown. Whorls 6–7, increasing regularly; body whorl large, rounded to slightly shouldered. Spire slightly elevated; suture rather impressed. Aperture crescent-shaped and obliquely opened. Peristome simple. Columellar margin simple and slightly reflected near umbilicus. Umbilicus narrowly opened (Fig. 17D–F).

**Figure 12.**
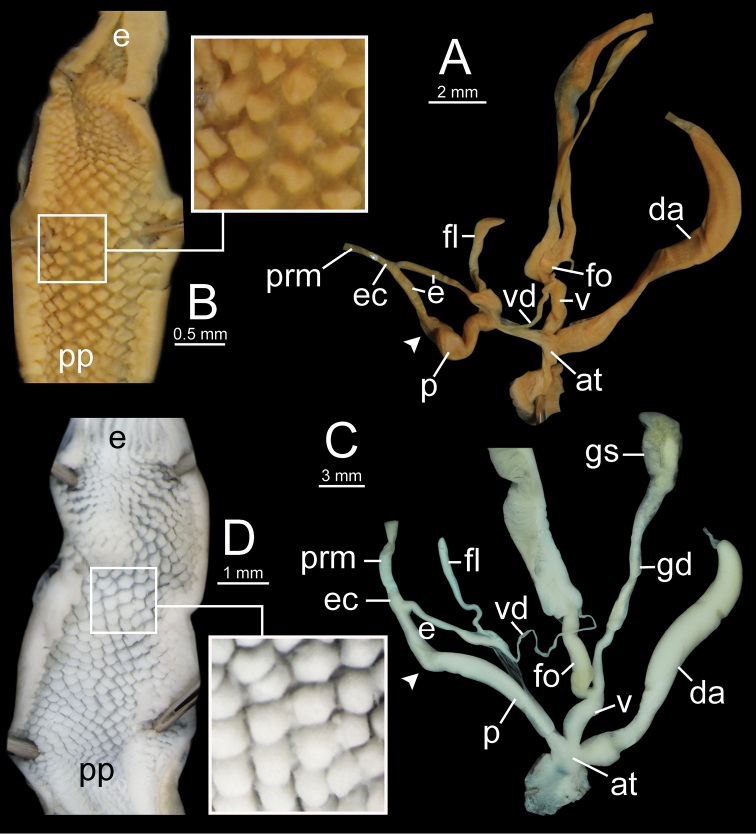
Genitalia of *Sarika resplendens*. **A, B** specimen NHMUK 1898.5.18.157 **A** general view of genital system and **B** internal structure of penis **C, D** specimen CUMZ 7851 **C** general view of the genital system and **D** internal structure of the penis. White arrowhead indicate the end of the penis.

***Genital organs*.** Atrium short. Penis cylindrical with thin penial sheath covering proximal penis. Inner sculpture of penis proximally more than ca. one-third of penial chamber with fine longitudinal penial pilaster to nearly smooth surface, and then modified from small to large cuboidal pilasters arranged in oblique rows. Epiphallus cylindrical, approximately as long as penis but narrower than penis. Epiphallic caecum short, straight, same diameter as epiphallus, located near middle of epiphallus. Penial retractor muscle thin and attached at tip of epiphallic caecum. Flagellum slender, approximately as long as penis. Vas deferens thin tube connecting distal epiphallus and free oviduct (Fig. 18C, D).

Vagina cylindrical, ca. one-third of penis length. Dart apparatus large, long cylindrical, and located on atrium at vagina and penis junction. Gametolytic sac enlarged and bulbous; gametolytic duct long cylindrical. Free oviduct cylindrical, nearly two times of vagina length, and proximal end encircled with thick tissue (Fig. 18C).

**Figure 13.**
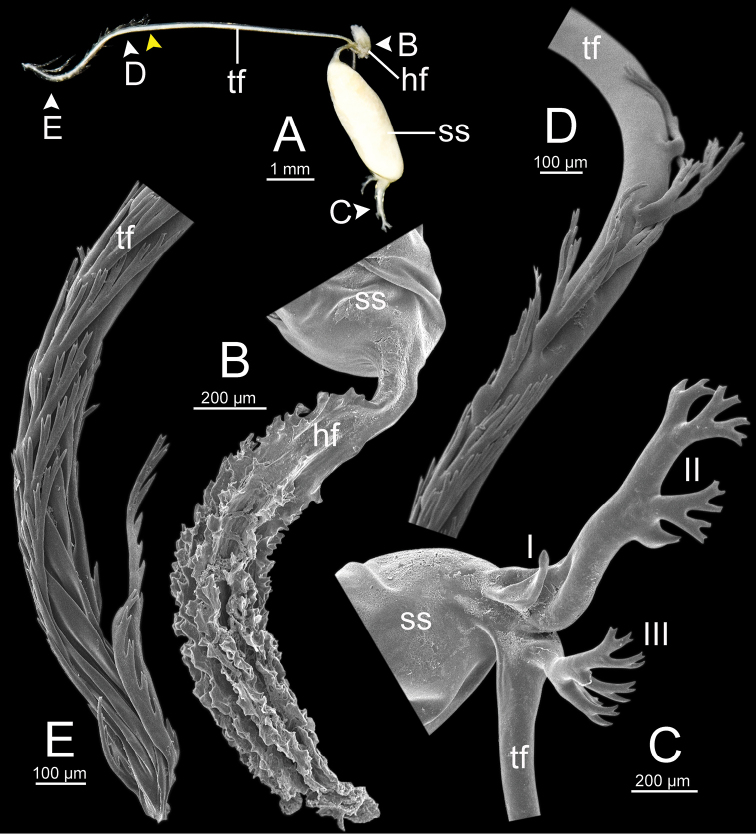
Spermatophore of *Sarika resplendens* specimen CUMZ 7876 **A** general view of the spermatophore **B** head filament with the position a bit twisted **C–E** tail filament showing **C** three spines located close to the sperm sac and **D** region with and without branching spines, and **E** branching spines on the tip region. Yellow arrowhead indicates the end of spines on the tip of the tail filament.

Spermatophore long and needle-shaped. Sperm sac enlarged and elongate-oval. Head filament was missing (incomplete spermatophore). Tail filament very long tube; region near sperm sac with three spines. Spine I simple, curved, and short. Spine II large and long, and branching part was missing. Spine III short and smaller than spine II, and branching part was missing. Region furthest away smooth and without spine; terminal part (more than ca. one-fourth of its length) with a series of short to long branching spines arranged in a row or encircled tail filament tip (Fig. 20).

**Figure 14.**
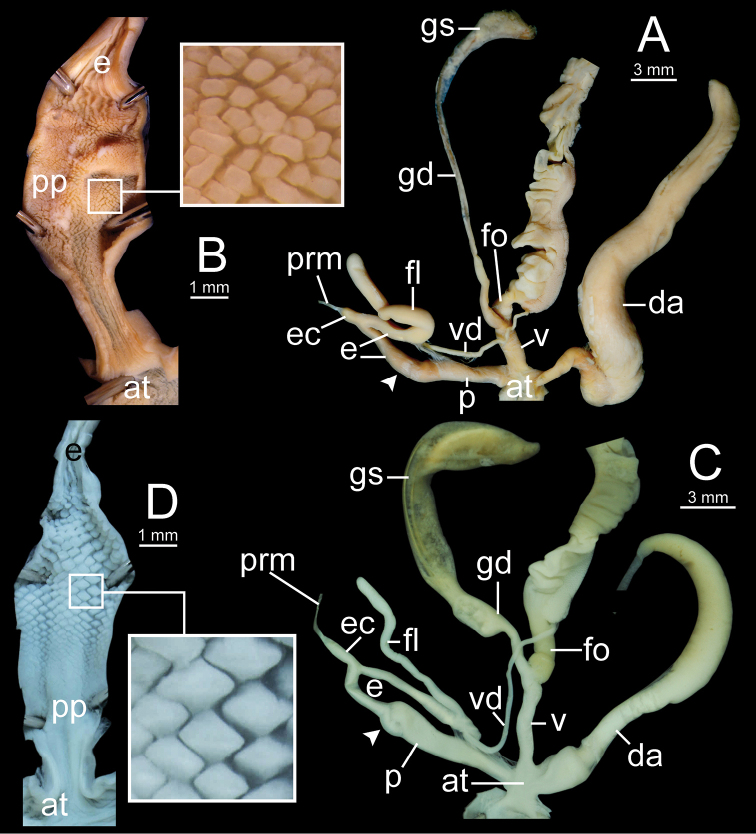
Genitalia. **A, B***Sarika dohrniana* specimen CUMZ 7633 **A** general view of the genital system and **B** internal structure of the penis **C, D***Sarika obesior* specimen CUMZ 7673 **C** general view of the genital system and **D** internal structure of penis. White arrowheads indicate the ends of the penes.

***Radula*.** Teeth with half row formula: 1–(11–12)–63. Central tooth symmetrical tricuspid; lateral teeth asymmetrical tricuspid; marginal teeth elongate bicuspid. Marginal teeth starting at approximately row number 11 or 12 (Fig. 30E).

***External features*.** Animal with reticulated skin and pale grey body, dark creamy mixing with grey foot sole and slightly dark grey caudal horn. Mantle edge well developed and same colour as body (Fig. 10A).

###### Distribution.

This species is known from the limestone outcrops in Kanchanaburi Province (Fig. 6).

**Figure 15.**
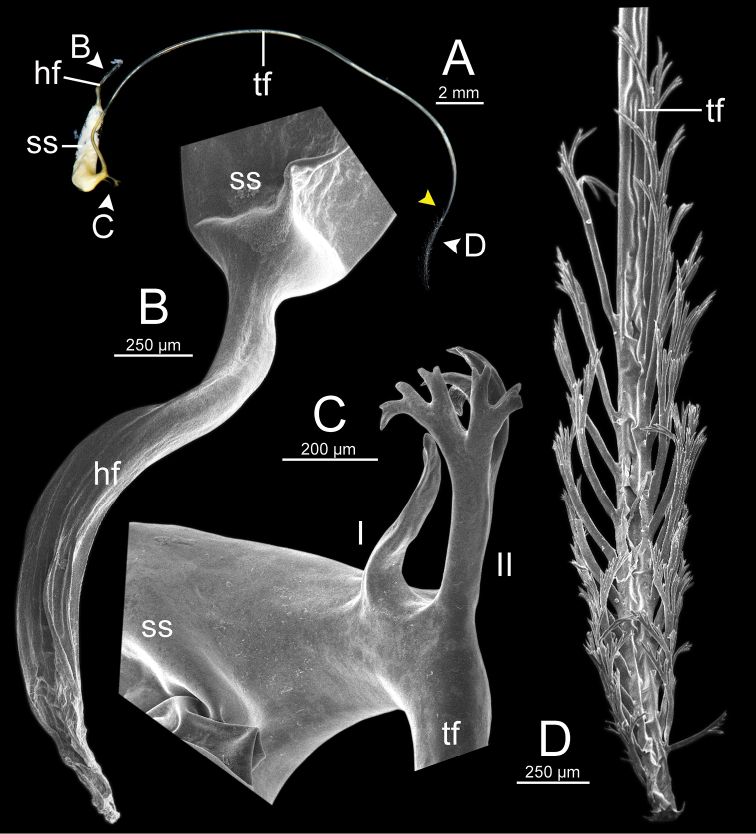
Spermatophore of *Sarika dohrniana* specimen CUMZ 7633 **A** general view of spermatophore **B** head filament **C, D** tail filament showing **C** two spines located close to the sperm sac and **D** tip region of tail filament. Yellow arrowhead indicates the end of the spines from the tip of the tail filament.

###### COI analysis.

The ML and BI analyses showed that the specimens of *S. heptagyra* (n = 3) formed a monophyletic group with very strong support (Fig. 1; BS = 100%, PP = 1). The mean intraspecific genetic distance of *S. heptagyra* was 3.5% (Table 2).

###### Remarks.

*Sarika heptagyra* is similar to *S. resplendens*. According to the phylogenetic tree, the relationship between *S. heptagyra* and *S. resplendens* is not clearly resolved (Fig. 1). The average interspecific sequence divergences between them were rather high at 7.7% which is in the recognised species range (4.6–12.0%) of interspecies sequence divergence of *Sarika* (see Table 2). Therefore, we have recognised *S. heptagyra* and *S. resplendens* as distinct biological species. The distinguishing character of *S. heptagyra* is its thin and long penial retractor muscle, while *S. resplendens* has very large and thickened penial retractor muscle (Table 2).

**Figure 16.**
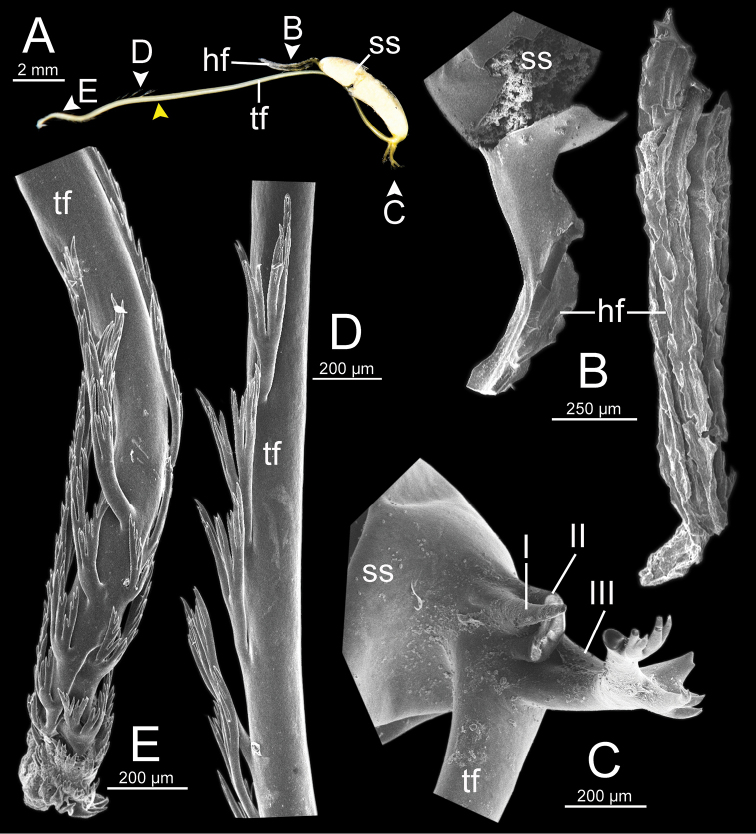
Spermatophore of *Sarika obesior* specimen CUMZ 7678 **A** general view of the spermatophore **B** head filament **C–E** tail filament **C** three spines located close to the sperm sac **D** region with and without branching spines, and **E** branching spines on the tip region. Yellow arrowhead indicates the end of the spines from the tip.

*Sarika heptagyra* seems to be indigenous in limestone habitats in western Thailand.

**Figure 17.**
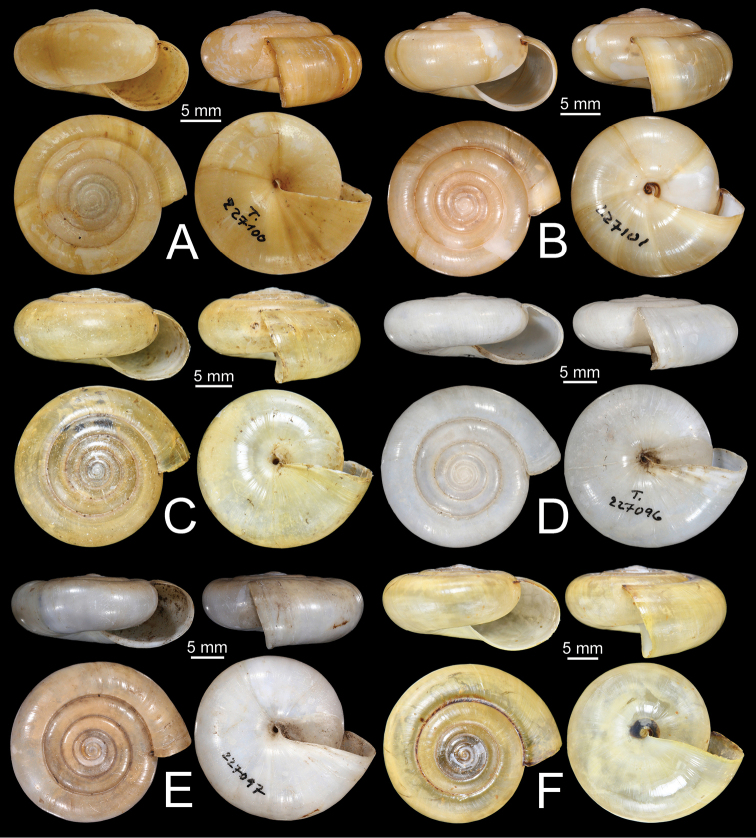
Shells of group I: *Sarika resplendens* group. **A–C***Sarika limbata***A** syntype SMF 227100, **B** syntype SMF 227101, and **C** specimen CUMZ 7652 **D–F***S. heptagyra***D** syntype SMF 227096 **E** syntype SMF 227097, and **F** specimen CUMZ 7231.

##### Sarika kawtaoensis

Taxon classificationAnimaliaStylommatophoraAriophantidae

Tomlin, 1929

9E5E687B-F36A-5BF5-A29D-C712F8F2D109

Figs 1 , 8 , 10B , 21A–D , 22A, B , 23 , 31A

Sarika kawtaoensis Tomlin, 1929: 15. Type locality: “Kaw Tao” [Ko Tao, Ko Pha-ngan District, Surat Thani Province, Thailand]. Hemmen and Hemmen 2001: 45.

###### Type material.

***Syntype***NMW 1955.158.01170 (two shells; Fig. 21A) from Kaw Tao [Tao Island, Ko Pangan, Surat Thani].

**Figure 18.**
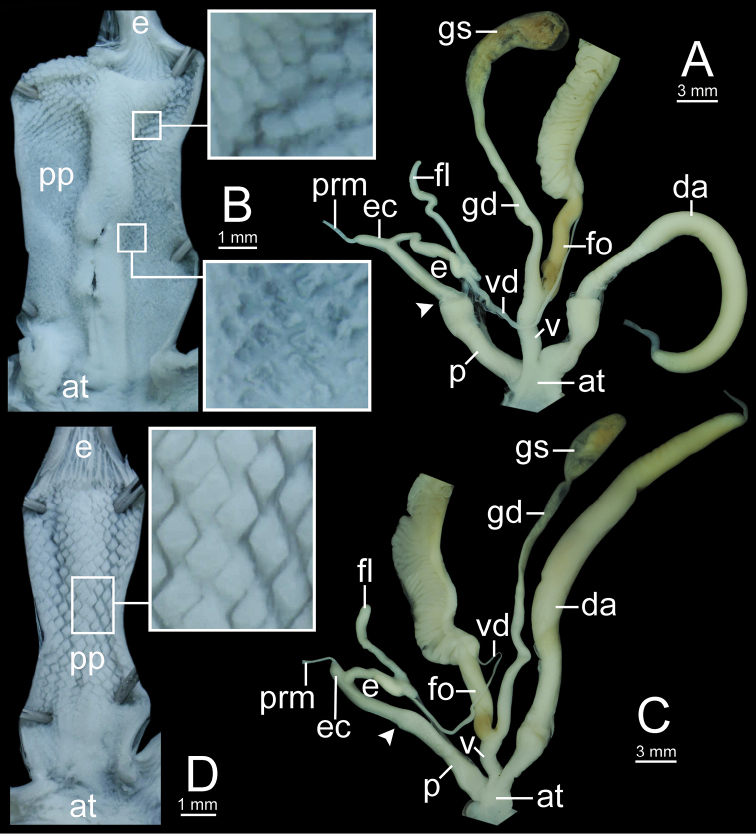
Genitalia. **A, B***Sarika limbata* specimen CUMZ 7652 **A** general view of the genital system and **B** internal structure of penis **C, D***S. heptagyra* specimen CUMZ 7279 **C** general view of the genital system and **D** internal structure of penis. White arrowheads indicate the ends of the penes.

###### Other material examined.

**Thailand-Southern.** Khao Phlu Cave, Pathio, Chumphon, 10°43'49.1"N, 99°19'13.9"E: CUMZ 7709. Ancient hot springs, Chaiya, Surat Thani, 9°21'51.8"N, 99°11'21.0"E: CUMZ 7706 (Fig. 21B). Wat Tham Sila Tiap, Tha Chana, Surat Thani, 9°30'58.8"N, 99°11'31.8"E: CUMZ 7717, 7750. Wat Wichit Ditatharam, Tha Chana, Surat Thani, 9°33'34.6"N, 99°10'18.3"E: CUMZ 7746. Wat Tham Yai, Tha Chana, Surat Thani, 9°32'21.7"N, 99°11'29.4"E: CUMZ 7752, 7783, 7806. Wat Rattanaram, Tha Chana, Surat Thani, 9°22'42.8"N, 99°11'25.4"E: CUMZ 7777. Tham Wang Badan Bureau of Monks, Khiri Rat Nikhom, Surat Thani, 8°56'13.0"N, 98°57'24.7"E: CUMZ 7707, 7751. Wat Sathit Khirirom, Khiri Rat Nikhom, Surat Thani, 9°01'48.4"N, 98°59'12.1"E: CUMZ 7236 (Fig. 21D), 7808. Limestone outcrop in Khiri Rat Nikhom, Khiri Rat Nikhom, Surat Thani, 9°00'19.2"N, 98°57'58.8"E: CUMZ 7798. Tham Bo Nam Thip Bureau of Monks, Kanchanadit, Surat Thani, 9°09'55.3"N, 99°35'20.5"E: CUMZ 7712. Wat Tham Khuha, Kanchanadit, Surat Thani, 9°09'17.3"N, 99°28'17.2"E: CUMZ 7802. Wat Khao Phra, Phrasaeng, Surat Thani, 8°37'32.4"N, 98°56'49.8"E: CUMZ 7713. Tham Nam Lod, Phrasaeng, Surat Thani, 8°40'40.4"N, 98°56'39.5"E: CUMZ 7714. Wat Santi Sirom, Phunphin, Surat Thani, 9°03'32.0"N, 99°15'05.4"E: CUMZ 7715. Wat Nakhawat, Phunphin, Surat Thani, 9°04'33.2"N, 99°09'54.0"E: CUMZ 7747. Limestone outcrop in Khlong Sok, Phanom, Surat Thani, 8°53'39.3"N, 98°33'10.7"E: CUMZ 7716, 7757. Limestone outcrop in Khlong Sok, Phanom, Surat Thani, 8°50'51.0"N, 98°44'32.8"E: CUMZ 7755. Wat Tham Wararam, Phanom, Surat Thani, 8°52'56.4"N, 98°39'49.6"E: CUMZ 7739, 7740. Limestone outcrop near Anurak Community Lodge, Phanom, Surat Thani, 8°53'20.3"N, 98°40'47.9"E: CUMZ 7741, 7742. Khao Sok, Phanom, Surat Thani, 8°54'55.6"N, 98°31'42.2"E: CUMZ 7743. Mae Yai Waterfall, Phanom, Surat Thani, 8°52'59.9"N, 98°29'58.5"E: CUMZ 7744, 7769. Limestone outcrop near Khao Sok Nature Resort, Phanom, Surat Thani, 8°54'22.6"N, 98°31'45.1"E: CUMZ 7766. Limestone outcrop near Khao Sok Evergreen House, Phanom, Surat Thani, 8°54'38.1"N, 98°31'47.2"E: CUMZ 7767. Limestone outcrop in Saphan Tao, Phanom, Surat Thani, 8°52'27.1"N, 98°38'46.5"E: CUMZ 7800. Limestone outcrop near Ratchaprapha Dam, Ban Ta Khun, Surat Thani, 8°58'20.9"N, 98°48'20.5"E: CUMZ 7756, 7809. Limestone outcrop near Khao Wong, Ban Ta Khun, Surat Thani, 8°56'12.4"N, 98°55'48.7"E: CUMZ 7794. Wat Khiri Rat Phatthana, Wiang Sa, Surat Thani, 8°31'38.6"N, 99°22'57.4"E: CUMZ 7745. Wat Na San, Na San, Surat Thani, 8°48'30.1"N, 99°22'10.4"E: CUMZ 7749, 7792, 7807. Tham Khao Khok Maharat Priest’s camp site, Na San, Surat Thani, 8°41'34.4"N, 99°22'45.8"E: CUMZ 7778. Limestone outcrop in Thong Nian, Khanom, Nakhon Si Thammarat, 9°17'15.7"N, 99°48'04.8"E: CUMZ 7710. Limestone outcrop near Khanom Seafood, Khanom, Nakhon Si Thammarat, 9°07'27.6"N, 99°52'59.3"E: CUMZ 7754, 7762. Lot cave, Nopphitam, Nakhon Si Thammarat, 8°44'10.0"N, 99°38'06.5"E: CUMZ 7735. Tham Talod, Thung Song, Nakhon Si Thammarat, 8°09'32.0"N, 99°40'41.8"E: CUMZ 7736. Wat Khuha Santayaram (Wat Tham Khao Daeng), Ron Phibun, Nakhon Si Thammarat, 8°14'38.3"N, 99°52'01.0"E: CUMZ 7737, 7799. Kaeo Surakan Cave, Lan Saka, Nakhon Si Thammarat, 8°21'40.4"N, 99°47'07.0"E: CUMZ 7738, 7765. Tham Nam Wang Sri Thammasokrat, Lan Saka, Nakhon Si Thammarat, 8°19'55.0"N, 99°49'59.8"E: CUMZ 7786. Wat Tham Kanlayanamit, Tham Phannara, Nakhon Si Thammarat, 8°30'48.2"N, 99°22'50.7"E: CUMZ 7748. Wat Tham Thong Panara, Tham Phannara, Nakhon Si Thammarat, 8°25'19.8"N, 99°22'46.4"E: CUMZ 7775. Phung Chang Cave, Mueang, Phang-nga, 8°26'33.1"N, 98°30'55.0"E: CUMZ 7719, 7758. Pha Phueng Cave, Mueang, Phang-nga, 8°28'31.8"N, 98°32'20.4"E: CUMZ 7722. Wat Suwan Khuha, Mueang, Phang-nga, 8°25'42.5"N, 98°28'18.1"E: CUMZ 7759, 7812. Tham Nam Phut, Mueang, Phang-nga, 8°27'45.0"N, 98°31'21.6"E: CUMZ 7760, 7784, 7796, 7799. Bang Toei Cave, Mueang, Phang-nga, 8°25'58.6"N, 98°34'01.8"E: CUMZ 7779. Wat Khiriwong, Thap Put, Phang-nga, 8°31'55.6"N, 98°34'37.0"E: CUMZ 7720, 7795, 7804. Tao Thong Waterfall, Thap Put, Phang-nga, 8°29'07.6"N, 98°35'08.5"E: CUMZ 7793. Mountain area near Ban Pak Khlong, Kapong, Phang-nga, 8°50'21.2"N, 98°27'41.5"E: CUMZ 7721. Tham Nalakiring Bureau of Monks, Plai Phraya, Krabi, 8°33'29.5"N, 98°51'44.0"E: CUMZ 7711. Wat Khao Hua Sing, Plai Phraya, Krabi, 8°30'41.4"N, 98°45'39.4"E: CUMZ 7725. Wat Tham Bun Raksa Phupharam, Lam Thap, Krabi, 8°02'13.9"N, 99°23'47.8"E: CUMZ 7773. Limestone outcrop near Than Bok Khorani, Ao Luek, Krabi, 8°23'19.3"N, 98°44'03.5"E: CUMZ 7718, 7723. Sa Yuan Thong Cave, Ao Luek, Krabi, 8°21'47.2"N, 98°44'44.3"E: CUMZ 7789. Limestone outcrop near Emerald Pool, Khlong Thom, Krabi, 7°55'30.3"N, 99°16'05.3"E: CUMZ 7813. Wat Tham Seua, Mueang, Krabi, 8°07'27.1"N, 98°55'26.1"E: CUMZ 7724, 7776, 7791, 7797. Limestone outcrop near Ban Thab Prik School, Mueang, Krabi, 8°10'50.2"N, 98°52'50.4"E: CUMZ 7781. Wat Tham Phraphut, Ratsada, Trang, 7°52'21.5"N, 99°43'40.9"E: CUMZ 7727. Limestone outcrop in Huai Yot, Huai Yot, Trang, 7°44'13.0"N, 99°39'23.0"E: CUMZ 7780. Khao Pu Chao Bureau of Monks, Na Yong, Trang, 7°33'31.2"N, 99°46'40.3"E: CUMZ 7801. Khanti Phon Cave, Thung Wa, Satun, 7°05'07.5"N, 99°47'53.4"E: CUMZ 7726, 7790. Wat Thung Khamin, Thung Wa, Satun, 7°02'57.5"N, 99°50'39.3"E: CUMZ 7728. Khao Thanan, Thung Wa, Satun, 7°03'37.0"N, 99°41'29.3"E: CUMZ 7788. Wat Kumphin Banpot, Khuan Kalong, Satun, 6°52'30.8"N, 100°01'02.4"E: CUMZ 7729. Ton Din Cave, Khuan Don, Satun, 6°43'35.5"N, 100°09'46.5"E: CUMZ 7753 (Fig. 21C), 7770, 7811. Khao Ok Tha Lu, Mueang, Phatthalung, 7°37'30.0"N, 100°05'30.0"E: CUMZ 7730. Wat Khuha Sawan, Mueang, Phatthalung, 7°37'14.1"N, 100°04'51.8"E: CUMZ 7733. Phra Non Cave, Mueang, Phatthalung, 7°40'53.0"N, 100°03'43.6"E: CUMZ 7734. Malai Cave, Mueang, Phatthalung, 7°38'07.9"N, 100°05'05.2"E: CUMZ 7805. Wang Thong Cave, Khuan Khanun, Phatthalung, 7°40'46.2"N, 100°00'49.9"E: CUMZ 7732. Tham Un Ya Ma Nee, Kong Ra, Phatthalung, 7°23'51.3"N, 99°58'36.1"E: CUMZ 7731. Khao Phaya Hong Cave, Kong Ra, Phatthalung, 7°27'48.7"N, 99°57'52.7"E: CUMZ 7814. Wat Tham Khao Chaison, Khao Chaison, Phatthalung, 7°27'00.7"N, 100°07'52.4"E: CUMZ 7785, 7774, 7787. Kathu Waterfall, Kathu, Phuket, 7°56'04.0"N, 98°19'22.4"E: CUMZ 7761. Limestone outcrop in Sakhu, Thalang, Phuket, 8°05'25.1"N, 98°17'54.7"E: CUMZ 7803. Khao Jung Lone Cave, Rattaphum, Songkhla, 7°11'25.8"N, 100°16'59.9"E: CUMZ 7771. Wat Charoen Phupha, Rattaphum, Songkhla, 7°08'51.1"N, 100°15'36.7"E: CUMZ 7810. Nang Phraya Laed Kaw Bureau of Monks, Sadao, Songkhla, 6°44'29.2"N, 100°15'28.6"E: CUMZ 7772.

**Figure 19.**
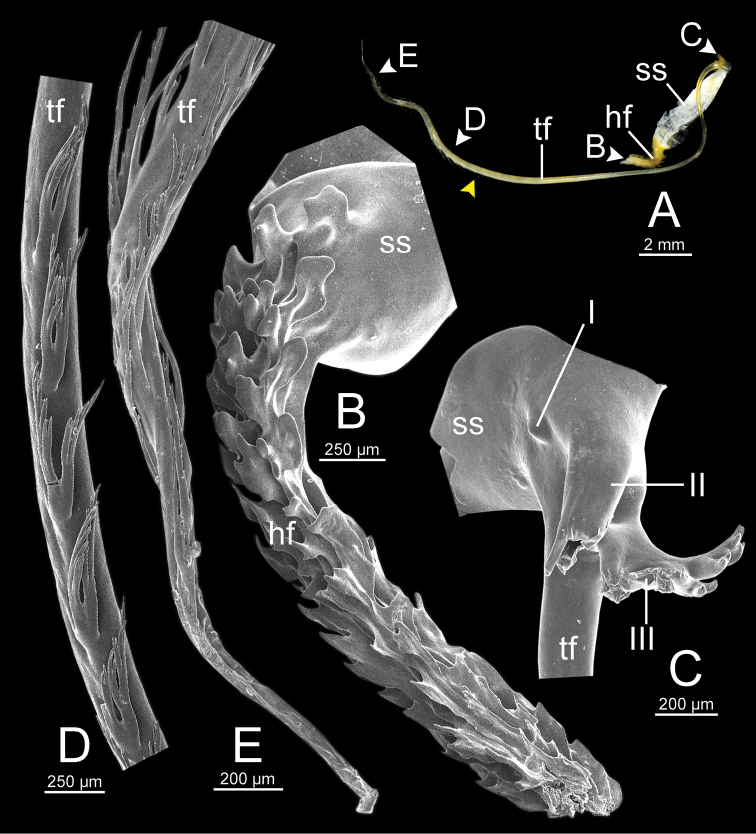
Spermatophore of *Sarika limbata* specimen CUMZ 7652 **A** general view of spermatophore, **B** head filament **C–E** tail filament showing **C** three spines located close to the sperm sac **D** region with and without branching spines, and **E** branching spines on the tip region. Yellow arrowheads indicate the ends of spines from the tip.

###### Diagnosis.

Shell large, depressed to globosely depressed and well-rounded body whorl. Animal with pale to dark grey body with five mantle lobes. Genitalia with a short straight epiphallic caecum. Inner penial sculpture with reticulated pilasters in proximal part and irregular surface folds arranged in oblique row at distal end. Spermatophore with irregularly acute-serrate longitudinal ridges on the head filament, tail filament with three spines, more than ca. half of its length with series of branching spines.

**Figure 20.**
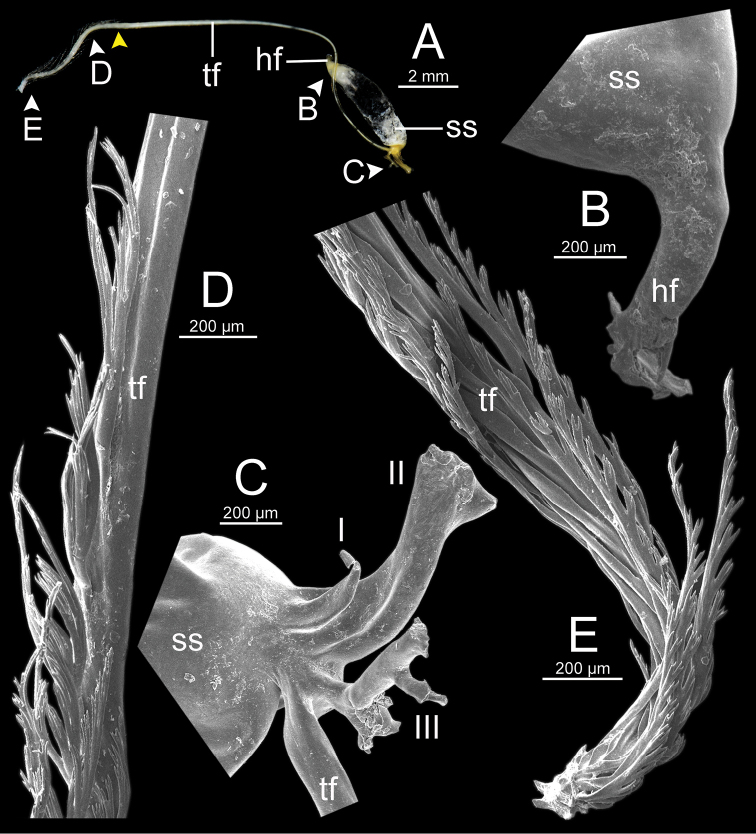
Spermatophore of *Sarika heptagyra* specimen CUMZ 7232 **A** general view of spermatophore, **B** head filament, and **C–E** tail filament showing **C** three spines located close to the sperm sac **D** region with and without branching spines, and **E** branching spines on the tip region. Yellow arrowhead indicates the end of the spines from the tip.

###### Description.

**Figure 21.**
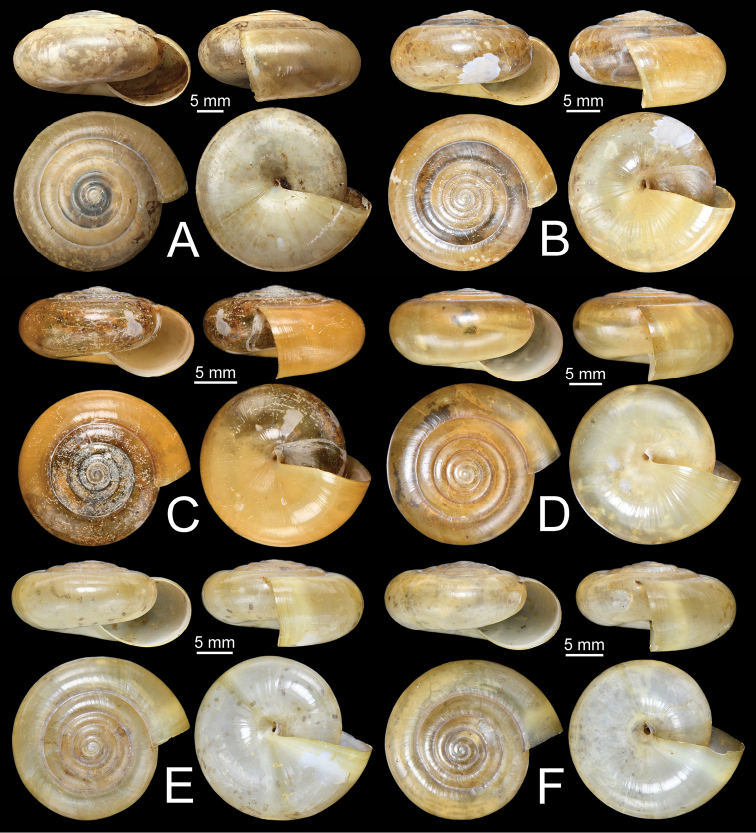
Shells of Group I: *Sarika resplendens* group. **A–D***Sarika kawtaoensis***A** syntype NMW 1955.158.01170 **B** specimen CUMZ 7706 **C** specimen CUMZ 7753 and **D** specimen CUMZ 7236 **E, F***S. caligina* sp. nov. **E** holotype CUMZ 7259, and **F** paratype CUMZ 7245.

***Shell*.** Shell depressed to globosely depressed, large size (shell width up to 26.6 mm, shell height up to 15.2 mm), and rather thin. Shell surface smooth, polished; shell colour pale warm brown to medium brown. Whorls 6–7, increasing regularly; body whorl large and well rounded. Spire moderately to very much elevated; suture impressed. Aperture crescent-shaped and obliquely opened. Peristome simple. Columellar margin simple and slightly reflected near umbilicus. Umbilicus narrowly opened (Fig. 21A–D).

**Figure 22.**
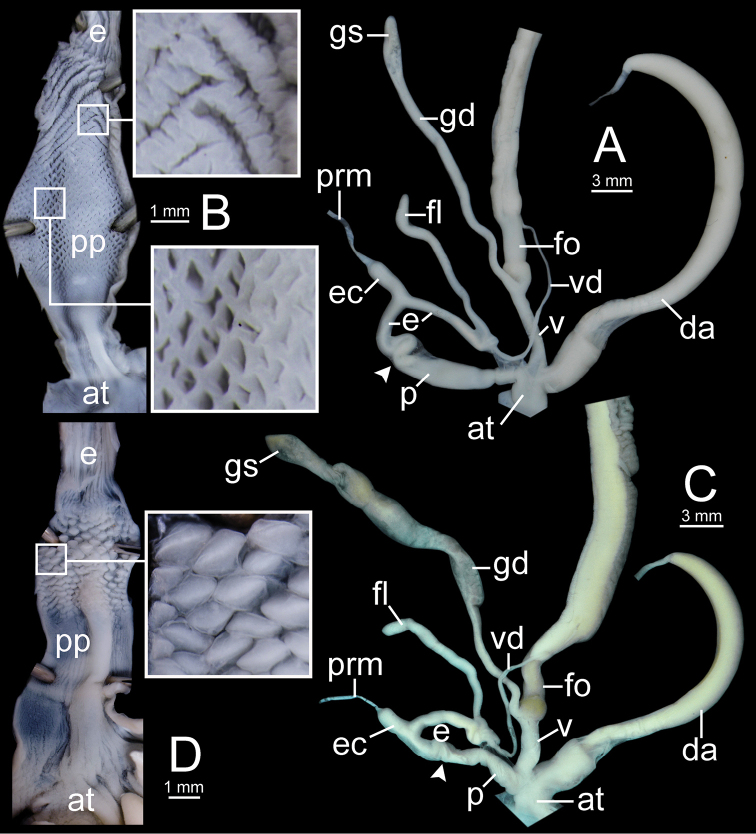
Genitalia. **A, B***Sarika kawtaoensis* specimen CUMZ 7762 **A** general view of the genital system and **B** internal structure of the penis **C, D***S. caligina* sp. nov. paratype CUMZ 7245 **C** general view of the genital system and **D** internal structure of the penis. White arrowheads indicate the ends of the penes.

***Genital organs*.** Atrium short. Penis cylindrical with thin penial sheath covering proximal penis. Inner sculpture of penis divided into three parts; proximally approximately one-third of penial chamber with very finely longitudinal penial pilasters to nearly smooth surface; middle approximately one-third of chamber covered with reticulated pilasters; distally approximately one-third pilaster transformed to irregular surface folds arranged in oblique row. Epiphallus cylindrical, approximately as long as penis and narrower penis. Epiphallic caecum short, straight, diameter slightly larger than epiphallus, and located near middle of epiphallus. Penial retractor muscle thin and attached at tip of epiphallic caecum. Flagellum long slender, approximately as long as epiphallus. Vas deferens thin tube connecting distal epiphallus and free oviduct (Fig. 22A, B).

Vagina cylindrical tube, approximately two-third of penis length. Dart apparatus large, long, cylindrical, and located on atrium at vagina and penis junction. Gametolytic sac bulbous; gametolytic duct long and cylindrical. Free oviduct cylindrical, almost as long as vagina and proximal end encircled with thick tissue (Fig. 22A).

**Figure 23.**
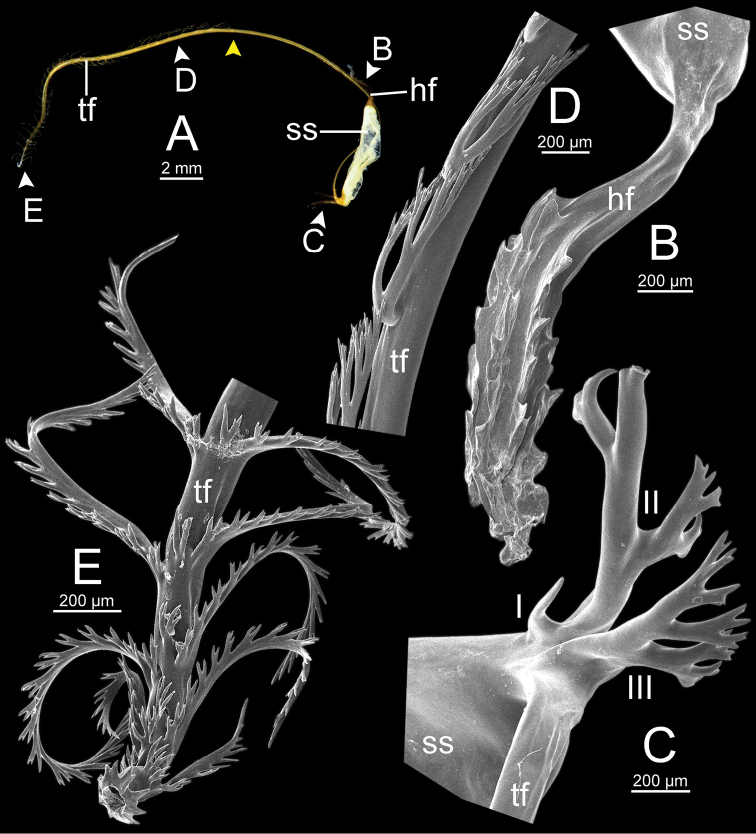
Spermatophore of *Sarika kawtaoensis* specimen CUMZ 7236 **A** general view of spermatophore **B** head filament **C–E** tail filament showing **C** three spines located close to the sperm sac **D** region with and without branching spines and **E** branching spines on the tip region. Yellow arrowhead indicates the end of the spines from the tip.

Spermatophore long and needle-shaped. Sperm sac enlarged and elongate-oval. Head filament gourd shape with irregularly acute-serrate longitudinal ridges. Tail filament very long tube; region near sperm sac with three spines. Spine I simple, little curved, and short. Spine II large and long, branching into many spinules near the tip. Spine II almost the same size as spine II, with complicated branching into small spinules. Region furthest away smooth and without spine; terminal part (more than ca. half of its length) with series of long branching spines arranged in a row, and then transformed very long serrate-like spines arranged in opposite rows near the tail filament tip (Fig. 23).

***Radula*.** Teeth with half row formula: 1–(13–14)–54. Central tooth symmetrical tricuspid; lateral teeth asymmetrical tricuspid; marginal teeth elongate bicuspid. Marginal teeth starting at ca. row number 13 or 14 (Fig. 31A).

***External features*.** Animal with reticulated skin and pale to dark grey body, pale grey foot sole, and dark grey caudal horn. Mantle edge well developed and same colour as body (Fig. 10B).

###### Distribution.

*Sarika kawtaoensis* is widely distributed throughout southern Thailand and occurs in both natural and populated community areas (Fig. 8).

###### COI analysis.

The ML and BI analyses of *S. kawtaoensis* revealed that the five individuals formed a monophyletic group with strong support (Fig. 1; BS = 91%, PP = 1), sister to *S. limbata* + *S. lactospira* sp. nov. yet only with BI support (Fig. 1; PP = 0.98). The mean intraspecific genetic distance of *S. kawtaoensis* was 3.3% (Table 2).

###### Remarks.

*Sarika kawtaoensis* is a variable species in terms of shell shape ranging from nearly flattened (Fig. 21D) to a low-conical spire (Fig. 21A). The reproductive organs in these two shell morphs are identical. In addition, the DNA phylogeny also revealed that these shell variations grouped together with strong support within the clade of *S. kawtaoensis* (Fig. 1).

##### Sarika caligina

Taxon classificationAnimaliaStylommatophoraAriophantidae

Pholyotha & Panha sp. nov.

2A2382D8-2B87-5031-A417-C9B7DDF109A7

http://zoobank.org/3ADF3132-BB2F-40B6-B050-8469075BBD7B

**Figure 24.**
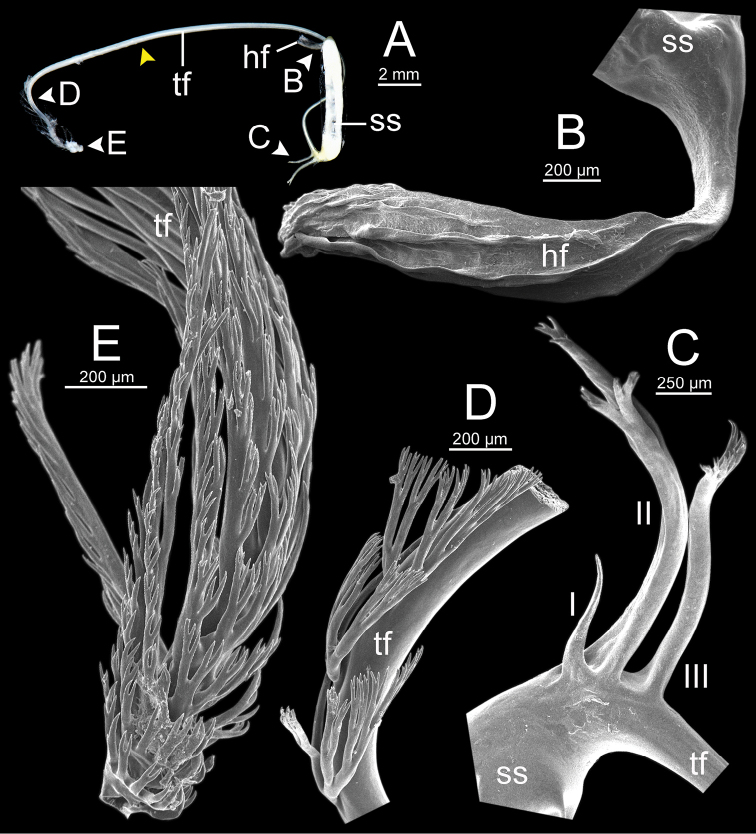
Spermatophore of *Sarika caligina* sp. nov. paratype CUMZ 7245 **A** general view of spermatophore **B** head filament **C–E** tail filament showing **C** three spines located close to the sperm sac **D** region with and without branching spines, and **E** branching spines on the tip region. Yellow arrowhead indicates the end of the spines from the tip.

Figs 1 , 6 , 10C , 21E, F , 22C, D , 24 , 31B

###### Type material.

***Holotype***CUMZ 7259 (Fig. 21E, width 23.6 mm, height 11.5 mm). ***Paratypes***CUMZ 7245 (17 shells and nine specimens preserved in ethanol; Fig. 21F, width 23.4 mm, height 11.9 mm) CUMZ 7246 (five shells and 15 specimens preserved in ethanol), CUMZ 7266 (eight shells), NHMUK 20200281 (two shells), SMF (two shells), ZRC.MOL.017026 (two shells).

###### Other material examined.

**Thailand-Central.** Wat Tham Mongkut, Phra Phutthabat, Saraburi, 14°40'40.6"N, 100°50'32.3"E: CUMZ 7260. Tham Rakhang-Tham Kin Non, Phra Phutthabat, Saraburi, 14°42'57.2"N, 100°47'50.3"E: CUMZ 7261. Wat Tham Osot, Muak Lek, Saraburi, 14°42'35.8"N, 101°07'02.5"E: CUMZ 7263. Wat Tham Rattana Buppha, Muak Lek, Saraburi, 14°41'35.3"N, 101°07'51.6"E: CUMZ 7264. Muak Lek Waterfall, Muak Lek, Saraburi, 14°43'17.3"N, 101°11'08.6"E: CUMZ 7265. Wat Tham Phrom Sawat, Phatthana Nikhom, Lopburi, 14°45'32.0"N, 100°56'22.4"E: CUMZ 7262.

###### Type locality.

Limestone outcrop with small shrubs at Wat Tham Si Wilai, Chaloem Phra Kiat, Saraburi, Thailand, 14°42’43.9"N, 100°52’01.3"E.

###### Diagnosis.

Shell large, depressed, and pale brown with well-rounded body whorl. Animal with blackish body and five mantle lobes. Genitalia with large, straight epiphallic caecum and triangular prism penial pilasters. Spermatophore: head filament with irregularly smooth longitudinal ridges; tail filament near sperm sac with three spines and terminal part of tail filament more than ca. half of its length with series of several branching spines.

###### Description.

***Shell*.** Shell depressed, large size (shell width up to 25.7 mm, shell height up to 12.3 mm), and thin. Surface smooth and polished; shell colour pale brown. Whorls 6–6½, increasing regularly; body whorl large and well rounded. Spire moderately elevated; suture impressed. Aperture crescent-shaped and obliquely opened. Peristome simple. Columellar margin simple and slightly reflected near umbilicus. Umbilicus narrowly opened (Fig. 21E, F).

***Genital organs*.** Atrium short. Penis cylindrical with thin penial sheath covering proximal penis. Inner sculpture of penis proximally more than ca. two-third of penial chamber with very finely longitudinal penial pilasters to nearly smooth surface, and then gradually modified from small to large rhomboid pilasters with acute angle on top (triangular prism). Epiphallus cylindrical, slightly longer than penis. Epiphallic caecum large, straight and located proximally far from middle of epiphallus. Penial retractor muscle thin and attached at tip of epiphallic caecum. Flagellum long, slender and slightly longer than epiphallus. Vas deferens thin tube connecting distal epiphallus and free oviduct (Fig. 22C, D).

Vagina cylindrical and approximately half of penis length. Dart apparatus large, long cylindrical, and located on atrium at vagina and penis junction. Gametolytic sac enlarged and bulbous; gametolytic duct enlarged and cylindrical (spermatophore inside). Free oviduct cylindrical, slightly shorter than total vagina length and proximal end encircled with thick tissue (Fig. 22C).

Spermatophore long and needle-shaped. Sperm sac enlarged and elongate-oval. Head filament gourd shape with irregularly smooth longitudinal ridges. Tail filament very long tube; region near sperm sac with three spines. Spine I simple, long, and slightly curved. Spine II large and long, branching into short spinules near the tip. Spine III shorter than spine II, branching into small and short spinules at the tip. Region furthest away smooth and without spine; terminal part (more than ca. half of its length) with series of short to long complicated branching spines arranged in a row or encircled the tail filament tip (Fig. 24).

***Radula*.** Teeth with half row formula: 1–(22–23)–66. Central tooth symmetrical tricuspid; lateral teeth asymmetrical tricuspid; marginal teeth elongate bicuspid. Marginal teeth starting at approximately row number 22 or 23 (Fig. 31B).

***External features*.** Animal with reticulated skin and very dark grey body. Foot sole and caudal foss present; caudal horn raised. Five mantle lobes well developed, same colour as body (Fig. 10C).

###### Etymology.

The specific name *caligina* is from the Latin *caliginis* meaning mist, darkness and refers to the blackish colour of body, which characterises this species.

###### Distribution.

*Sarika caligina* sp. nov. occurs in limestone habitats in central Thailand (Fig. 6). However, its habitats are threatened because many small karsts in this area have active quarries for the cement industry.

###### COI analysis.

The ML and BI analyses revealed that the specimens of *S. caligina* sp. nov. (n = 3) formed a monophyletic group with very strong support (Fig. 1; BS = 100%, PP = 1). The mean intraspecific genetic distance of *S. caligina* sp. nov. was 1.1% (Table 2).

###### Remarks.

This new species has a shell morphology that resembles *S. resplendens*, *S. heptagyra*, *S. limbata* and *S. kawtaoensis*. The distinguishing characters of this new species are its triangular prism-shaped penial pilasters, while *S. resplendens* and *S. heptagyra* have cuboidal penial pilasters, and *S. limbata* and *S. kawtaoensis* have reticulated penial pilasters. Moreover, *S. caligina* sp. nov. has irregularly smooth ridges on the head filament of the spermatophore, while *S. resplendens* has obtuse-serrate ridges, *S. limbata* has plate-like and acute-serrate ridges, and *S. kawtaoensis* has acute-serrate ridges. Unfortunately, the head filament of *S. heptagyra* was not available for comparison.

Although *S. caligina* sp. nov. and *S. obesior* have a similar penial sculpture, the two species can be distinguished by their spermatophores. *Sarika caligina* sp. nov. has irregularly smooth ridges on the head filament and approximately half of the tail filament contains branching spines, whereas *S. obesior* has obtuse-serrate ridges on the head filament and approximately one-third of the tail filament contains branching spines.

##### Sarika lactospira

Taxon classificationAnimaliaStylommatophoraAriophantidae

Pholyotha & Panha sp. nov.

EEBEE371-7E9D-5794-8A4B-B26CAD6F2781

http://zoobank.org/FAD64134-9A20-4C07-8DA2-43A8E8A37EED

**Figure 25.**
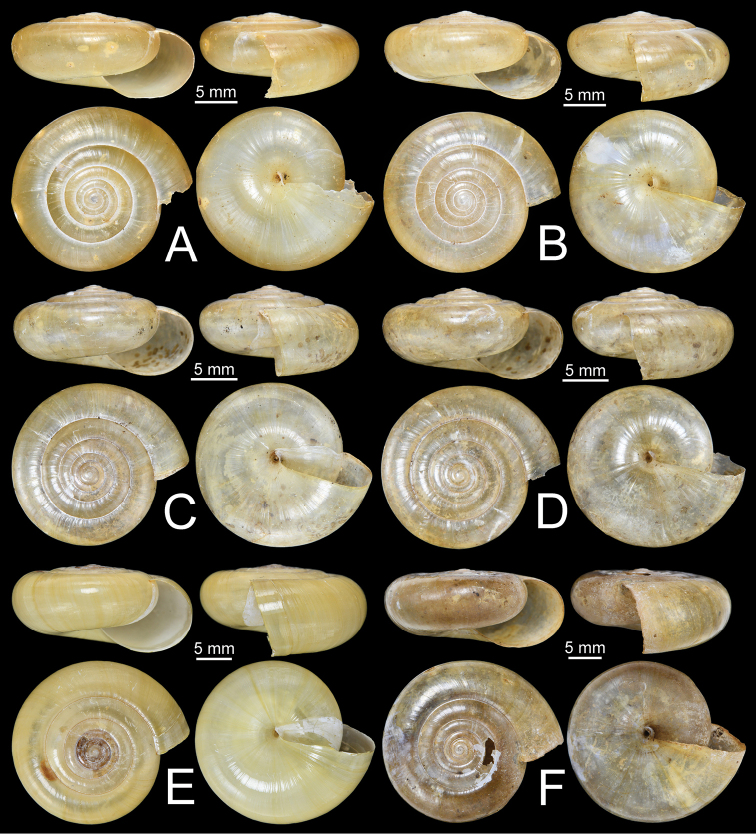
Shells of group I: *Sarika resplendens* group. **A, B***Sarika lactospira* sp. nov. **A** holotype CUMZ 7286 and **B** paratype CUMZ 7287 **C, D***S. megalogyne* sp. nov. **C** holotype CUMZ 7521 and **D** paratype CUMZ 7522 **E, F***S. subheptagyra* sp. nov. **E** holotype CUMZ 7506 and **F** paratype CUMZ 7507.

**Figure 26.**
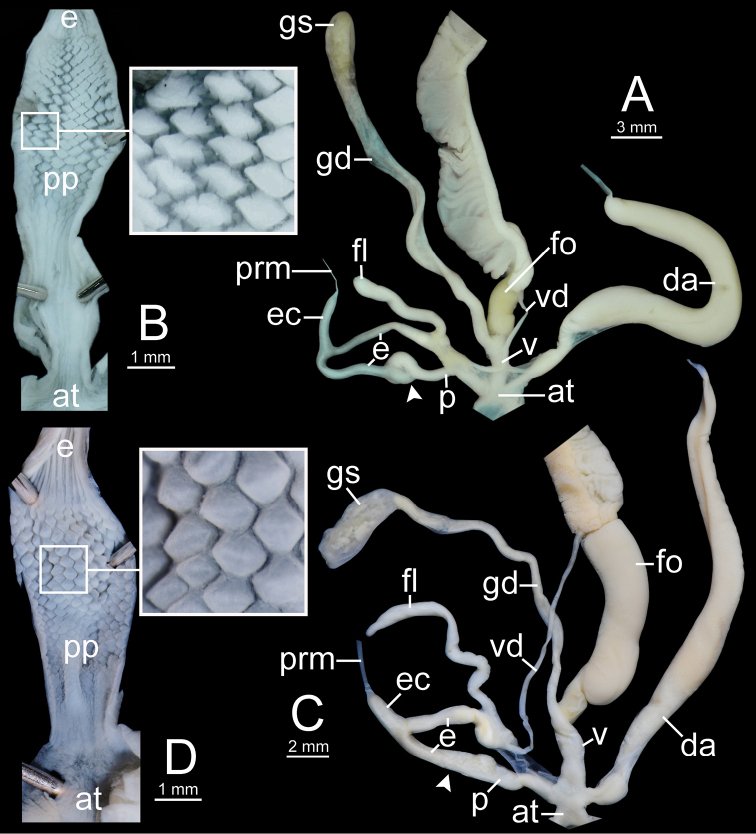
Genitalia. **A, B***Sarika lactospira* sp. nov. specimen CUMZ 7291 **A** general view of genital system **B** internal structure of penis **C, D***S. megalogyne* sp. nov. paratype CUMZ 7522 **C** general view of genital system **D** internal structure of penis. White arrowheads indicate the ends of the penes.

Figs 1 , 8 , 10D , 25A, B , 26A, B , 31C

###### Type material.

***Holotype***CUMZ 7286 (Fig. 25A, width 21.2 mm, height 10.0 mm). ***Paratypes***CUMZ 7287 (28 shells and three specimens preserved in ethanol; Fig. 25B, width 20.5 mm, height 10.2 mm), NHMUK 20200282 (two shells), SMF (two shells), ZRC.MOL.017027 (two shells).

###### Other material examined.

**Thailand-Southern.** Limestone outcrop in Don Sak, Don Sak, Surat Thani, 9°19'21.9"N, 99°44'38.2"E: CUMZ 7288, 7293. Khao Kloi Monastery, Don Sak, Surat Thani, 9°16'47.1"N, 99°44'11.7"E: CUMZ 7290. Limestone outcrop in Nang Kam Beach, Don Sak, Surat Thani, 9°18'54.0"N, 99°45'39.4"E: CUMZ 7294. Khiri Wong Cave, Don Sak, Surat Thani, 9°12'15.9"N, 99°39'45.0"E: CUMZ 7295. Wat Pra Puttabhat Sri Suratthani, Kanchanadit, Surat Thani, 9°11'17.2"N, 99°34'50.6"E: CUMZ 7292. Khao Phanom Wang Bureau of Monks, Kanchanadit, Surat Thani, 9°05'30.9"N, 99°36'19.0"E: CUMZ 7296. Limestone outcrop in Thong Thian, Khanom, Nakhon Si Thammarat, 9°13'27.2"N, 99°50'37.4"E: CUMZ 7289. Khao Krot Bureau of Monks, Khanom, Nakhon Si Thammarat, 9°14'22.5"N, 99°48'04.9"E: CUMZ 7291.

###### Type locality.

Wat Ao Sadet, Khanom, Nakhon Si Thammarat, Thailand, 9°17'20.9"N, 99°47'13.8"E.

###### Diagnosis.

Shell large, depressed, pale yellowish brown with slightly shouldered body whorl and pale milky subsutural band. Animal with grey body and five mantle lobes. Genitalia with a straight epiphallic caecum and triangular prism pilasters on inner penial sculpture.

###### Description.

***Shell*.** Shell depressed, large size (shell width up to 23.6 mm, shell height up to 11.8 mm) and thin. Surface smooth and shiny; shell colour pale yellowish brown. Whorls 6–6½, increasing regularly; body whorl large and slightly shouldered. Spire moderately elevated; suture impressed and with narrow pale milky to whitish subsutural band. Aperture crescent-shaped and obliquely opened. Peristome simple. Columellar margin simple and slightly reflected near umbilicus. Umbilicus narrowly opened (Fig. 25A, B).

***Genital organs*.** Atrium short. Penis cylindrical with thin penial sheath covering proximal penis. Inner sculpture of penis proximally more than ca. half of penial chamber with small longitudinal penial pilasters, and then gradually transformed from small to large rhomboid pilasters with acute angle on top (triangular prism). Epiphallus cylindrical and slightly shorter than twice the penis length. Epiphallic caecum long, straight, similar diameter with epiphallus and located proximally near middle of epiphallus. Penial retractor muscle thin and attached at tip of epiphallic caecum. Flagellum slender, approximately half length of epiphallus. Vas deferens thin tube connecting distal epiphallus and free oviduct (Fig. 26A, B).

Vagina cylindrical and approximately half of penis length. Dart apparatus large, long cylindrical, and located on atrium at vagina and penis junction. Gametolytic sac enlarged and bulbous; gametolytic duct long and enlarged (damaged spermatophore inside). Free oviduct large cylindrical, approximately as long as vagina length, and proximal end encircled with thick tissue (Fig. 26A).

***Radula*.** Teeth with half row formula: 1–(17–18)–64. Central tooth symmetrical tricuspid; lateral teeth asymmetrical tricuspid; marginal teeth elongate bicuspid. Marginal teeth starting at approximately row number 17 or18 (Fig. 31C).

***External features*.** Animal with reticulated skin and body darker grey above and paler grey near foot sole. Caudal foss and caudal horn present. Five mantle lobes well developed and pale grey colour (Fig. 10D).

###### Etymology.

The specific name *lactospira* is derived from the Latin words *lacteus* meaning milky and *spira* meaning coil. It refers to the pale milky colour below suture.

###### Distribution.

*Sarika lactospira* sp. nov. is restricted to limestone habitats in Surat Thani and Nakhon Si Thammarat provinces (Fig. 8).

###### COI analysis.

The ML and BI analyses revealed that the four individuals of *S. lactospira* sp. nov. formed a monophyletic group with very strong support (Fig. 1; BS = 100%, PP = 1). The mean intraspecific genetic distance of *S. lactospira* sp. nov. was 2.3% (Table 2).

###### Remarks.

*Sarika lactospira* sp. nov. differs from all other species in the *Sarika resplendens* group by having a shouldered body whorl and usually with a narrow whitish subsutural band. In comparison the shell of *S. lactospira* sp. nov. has shouldered body whorl, while, *S. resplendens*, *S. obesior*, *S. limbata*, *S. kawtaoensis*, *S. caligina* sp. nov., and *S. subheptagyra* sp. nov. have a well-rounded body whorl, and *S. dohrniana* has a rounded to slightly obtusely angulated body whorl.

Compared among the shouldered body whorl species, the distinguishing character of *S. lactospira* sp. nov. is the triangular penial pilasters, while *S. heptagyra* has cuboidal penial pilasters.

Although *S. lactospira* sp. nov. and *S. megalogyne* sp. nov. have a similar shell and penial sculpture, the free oviduct and flagellum of *S. lactospira* sp. nov. are much shorter than those of *S. megalogyne* sp. nov. In addition, the COI sequence divergences between both species were rather high (7.1%). Unfortunately, the spermatophore of *S. lactospira* sp. nov. was not available for comparison.

##### Sarika megalogyne

Taxon classificationAnimaliaStylommatophoraAriophantidae

Pholyotha & Panha sp. nov.

71C59446-2549-5E26-9BE9-93FDE5165523

http://zoobank.org/EB3A8C40-4AFB-4A24-91A0-EFFA2DC4FCF5

**Figure 27.**
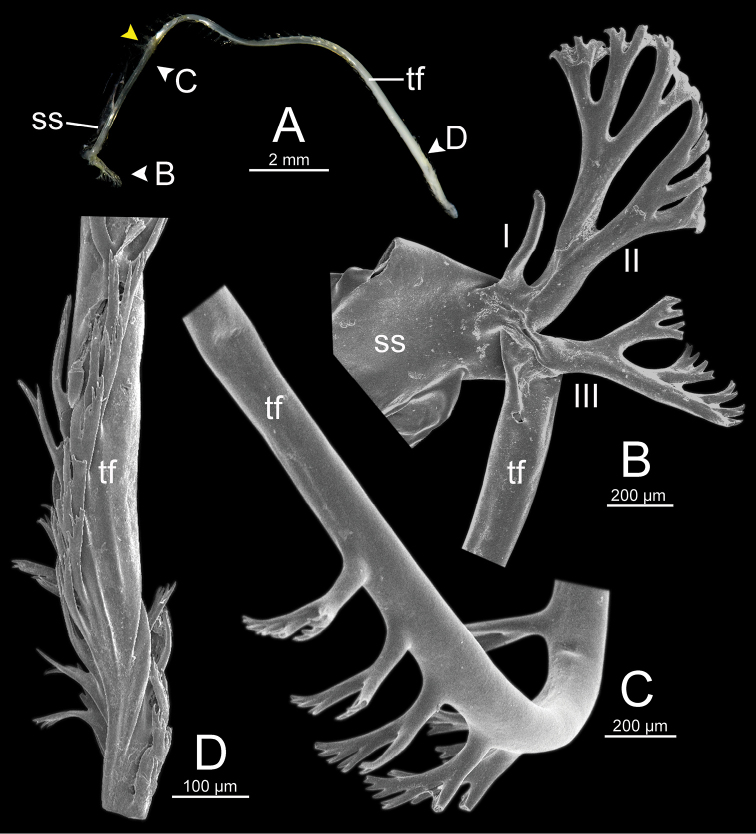
Spermatophore of *Sarika megalogyne* sp. nov. paratype CUMZ 7522 **A** general view of the spermatophore and **B–D** tail filament showing **B** three spines located close to the sperm sac **C** region with and without branching spines, and **D** branching spines on the tip region. Yellow arrowheads indicate the end of the spines from the tip.

Figs 1 , 7 , 10E , 25C, D , 26C, D , 27 , 31D

###### Type material.

***Holotype***CUMZ 7521 (Fig. 25C, width 19.5 mm, height 9.8 mm). ***Paratypes***CUMZ 7522 (23 shells and 34 specimens preserved in ethanol; Fig. 25D, width 19.5 mm, height 10.0 mm), CUMZ 7238 (10 specimens preserved in ethanol), CUMZ 7529 (12 shells and 78 specimens preserved in ethanol), CUMZ 7530 (24 shells), CUMZ 7536 (16 specimens preserved in ethanol), CUMZ 7537 (three specimens preserved in ethanol), NHMUK 20200283 (two shells), SMF (two shells), ZRC.MOL.017028 (two shells).

###### Other material examined.

**Thailand-Western.** Khao Pho Cave, Bang Saphan Noi, Prachuap Khiri Khan, 10°59'25.2"N, 99°21'32.8"E: CUMZ 7540. **Thailand-Southern.** Pla Cave, Thung Tako, Chumphon, 10°07'58.4"N, 99°08'14.5"E: CUMZ 7523. Tham Sing, Mueang, Chumphon, 10°25'45.9"N, 99°03'11.3"E: CUMZ 7527. Wat Bonphot Phisai, Lang Suan, Chumphon, 9°56'23.8"N, 99°08'51.4"E: CUMZ 7528. Tham Pisadan Bureau of Monks, Tha Sae, Chumphon, 10°45'36.7"N, 99°13'45.8"E: CUMZ 7538. Limestone outcrop in Saphli, Pathio, Chumphon, 10°33'12.0"N, 99°16'34.5"E: CUMZ 7524, 7532. Malagor Cave, Pathio, Chumphon, 10°52'42.9"N, 99°30'30.3"E: CUMZ 7525. Nang Thong Cave, Pathio, Chumphon, 10°40'14.3"N, 99°17'35.9"E: CUMZ 7526. Wat Tham Khao Plu, Pathio, Chumphon, 10°43'49.6"N, 99°19'19.7"E: CUMZ 7539. Wat Tham Khao Bang Siap, Pathio, Chumphon, 10°40'06.7"N, 99°17'37.9"E: CUMZ 7534. Ko Wiang, Pathio, Chumphon, 10°50'43.3"N, 99°28'46.5"E: CUMZ 7909. Cholkhiri Bureau of Monks, Sawi, Chumphon, 10°22'23.9"N, 99°03'38.1"E: CUMZ 7531. Limestone outcrop in Sawi, Sawi, Chumphon, 10°15'16.9"N, 99°10'01.2"E: CUMZ 7533. Wat Tham Khao Lan, Sawi, Chumphon, 10°15'47.0"N, 99°10'28.1"E: CUMZ 7535.

###### Type locality.

Limestone outcrop at Khao Ma Rong Cave, Bang Saphan, Prachuap Khiri Khan, Thailand, 11°12'09.2"N, 99°29'48.7"E.

###### Diagnosis.

Shell medium to large, depressed and very pale brown with well-rounded to slightly shouldered body whorl. Animal with grey body and five mantle lobes. Genitalia with a straight epiphallic caecum, very large free oviduct and triangular prism-shaped pilasters on inner penial sculpture. Spermatophore: tail filament near sperm sac with three spines and terminal part more than ca. three-quarters of its length with series of several branching spines.

**Figure 28.**
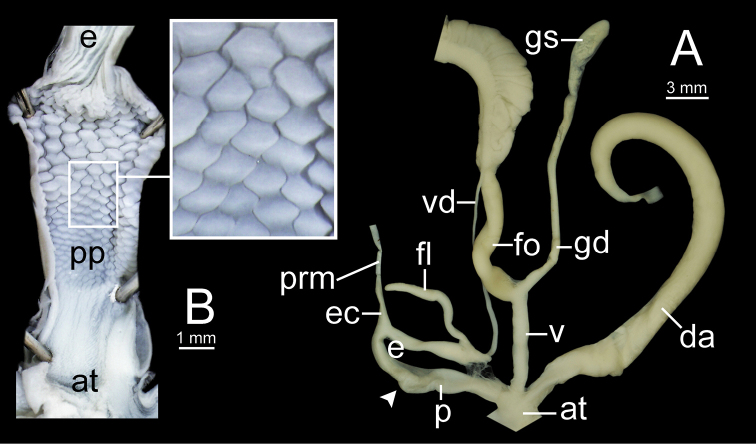
Genitalia. **A, B***Sarika subheptagyra* sp. nov. paratype CUMZ 7507 **A** general view of the genital system and **B** internal structure of the penis. White arrowhead indicate the end of the penis.

###### Description.

***Shell*.** Shell depressed, medium to large size (shell width up to 21.2 mm, shell height up to 11.3 mm), and thin. Surface smooth and glossy; shell colour very pale brown. Whorls 6–6½, increasing regularly; body whorl slightly well rounded to slightly shouldered. Spire moderately to very much elevated; suture impressed. Aperture crescent-shaped and obliquely opened. Peristome simple. Columellar margin simple and slightly reflected near umbilicus. Umbilicus narrowly opened (Fig. 25C, D).

***Genital organs*.** Atrium short. Penis cylindrical with thin penial sheath covering proximal penis. Inner sculpture of penis proximally more than ca. one-third of penial chamber with very finely longitudinal penial pilasters to nearly smooth surface, and then gradually modified from small to large rhomboid pilasters with acute angle on top (triangular prism). Epiphallus cylindrical, slightly longer than penis length. Epiphallic caecum large, straight, diameter slightly larger than epiphallus, and located proximally near middle of epiphallus. Penial retractor muscle thin and attached at tip of epiphallic caecum. Flagellum long slender and slightly longer than epiphallus. Vas deferens thin tube connecting distal epiphallus and free oviduct (Fig. 26C, D).

Vagina cylindrical, enlarged and slightly shorter than penis. Dart apparatus large, long cylindrical, and located on atrium at vagina and penis junction. Gametolytic sac enlarged and bulbous; gametolytic duct long and cylindrical. Free oviduct enlarged cylindrical, extremely long, approximately three times of vagina length (Fig. 26C).

**Figure 29.**
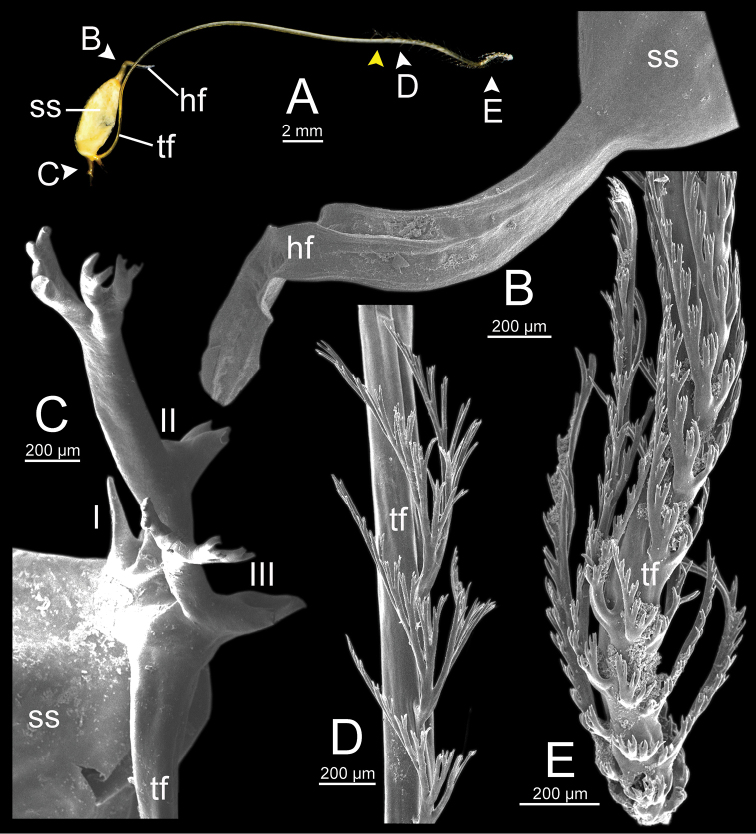
Spermatophore of *Sarika subheptagyra* sp. nov. paratype CUMZ 7507 **A** general view of spermatophore **B** head filament, and **C–E** tail filament showing **C** three spines located close to the sperm sac **D** region with and without branching spines, and **E** branching spines on the tip region. Yellow arrowhead indicates the ends of the spines from the tip.

Spermatophore long and needle-shaped. Sperm sac and head filament were missing. Tail filament long tube; region near sperm sac with three spines. Spine I simple and short. Spine II large, long, and with complicated branching spines into spinules near the tip. Spine III smaller than spine II and with complicated branching spines into spinules. Region furthest away smooth and without spine; terminal part (approximately three-quarters of its length) with series of long complicated branching spines into spinules arranged in a row, and then transformed to very long serrate-like spines arranged in opposite rows near the tail filament tip (Fig. 27).

***Radula*.** Teeth with half row formula: 1–(13–14)–55. Central tooth symmetrical tricuspid; lateral teeth asymmetrical tricuspid; marginal teeth elongate bicuspid. Marginal teeth starting at approximately row number 13 or 14 (Fig. 31D).

**Figure 30.**
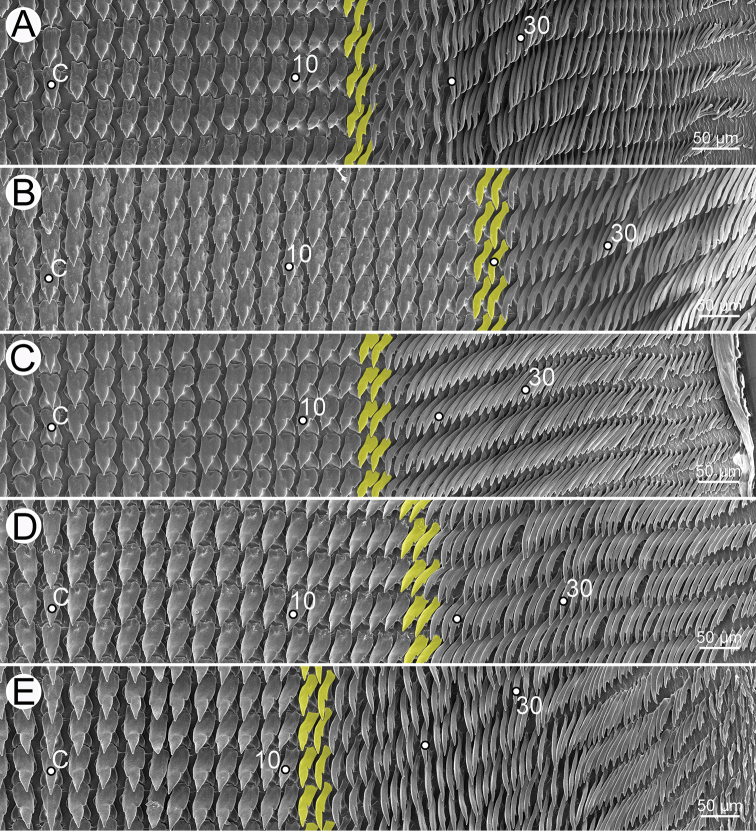
Representative SEM images of the radula. **A***Sarika resplendens* specimen CUMZ 7851 **B***S. dohrniana* specimen CUMZ 7633 **C***S. obesior* specimen CUMZ 7673 **D***S. limbata* specimen CUMZ 7652 **E***S. heptagyra* specimen CUMZ 7279. Central tooth indicated by ‘C’; yellow colour indicates lateral teeth in the transition to marginal teeth.

***External features*.** Animal with reticulated skin and body darker grey above and paler grey near foot sole. Caudal foss and caudal horn present. Five mantle lobes well developed and pale grey in colour (Fig. 10E).

**Figure 31.**
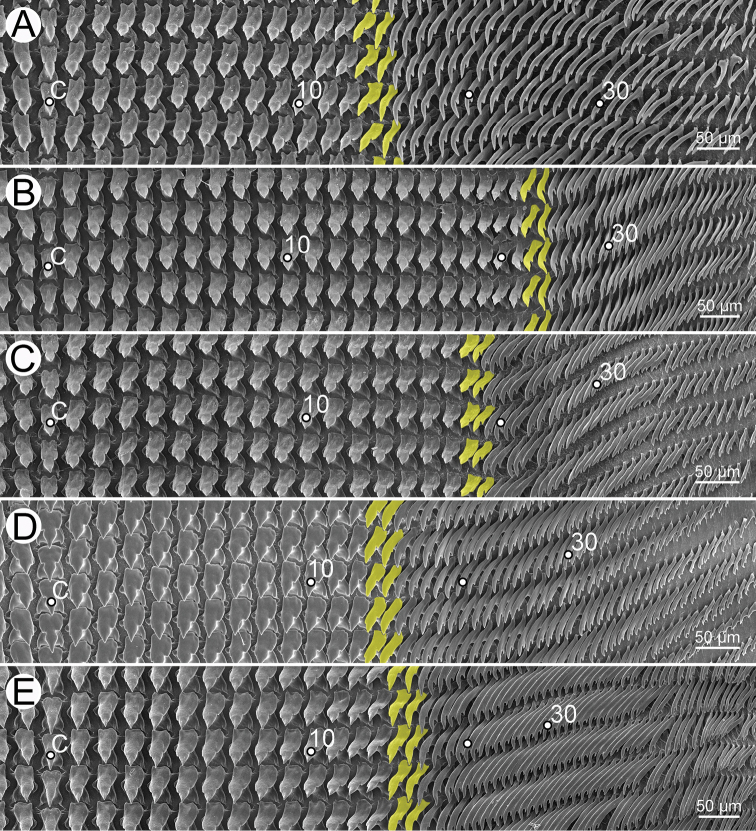
Representative SEM images of the radula. **A***Sarika kawtaoensis* specimen CUMZ**B***S. caligina* sp. nov. paratype CUMZ 7245 **C***S. lactospira* sp. nov. specimen CUMZ 7291 **D***S. megalogyne* sp. nov. specimen CUMZ 7531, and **E***S. subheptagyra* sp. nov. paratype CUMZ 7507. Central tooth indicated by ‘C’; yellow colour indicates lateral teeth in the transition to marginal teeth.

###### Etymology.

The specific name *megalogyne* is derived from the Greek word *megale* meaning large and the Greek *gyne* meaning female. It refers to the female part of genital organs with a very large free oviduct, which characterises this species.

###### Distribution.

*Sarika megalogyne* sp. nov. is common in Prachuap Khiri Khan and Chumphon provinces (Fig. 7). This species is often found under leaves of small trees and shrubs on limestones.

###### COI analysis.

The ML and BI analyses of *S. megalogyne* sp. nov. revealed that all samples (n = 3) formed a clade with very strong support (Fig. 1; BS = 100%, PP = 1). The mean intraspecific genetic distance of *S. megalogyne* sp. nov. was 1.4% (Table 2).

###### Remarks.

*Sarika megalogyne* sp. nov. and *S. caligina* sp. nov. have similar genitalia and penial sculpture. However, *S. megalogyne* sp. nov. has a much longer free oviduct and flagellum than *S. caligina* sp. nov. In addition, the spermatophore of *S. megalogyne* sp. nov. with more than ca. three-quarters of the tail filament contains branching spines, whereas the spermatophore of *S. caligina* sp. nov. with more than ca. only half of the tail filament contains branching spines. Furthermore, the genetic distance between these two species is high (7.4%).

##### Sarika subheptagyra

Taxon classificationAnimaliaStylommatophoraAriophantidae

Pholyotha & Panha sp. nov.

80CB62D8-CB85-5E77-95D2-AC4ED63D9B75

http://zoobank.org/9D3F1C60-3FDC-4950-AE84-AAE1B12863CE

Figs 1 , 6 , 10F , 25E, F , 28 , 29 , 31E

###### Type material.

***Holotype***CUMZ 7506 (Fig. 25E, width 25.5 mm, height 12.5 mm). ***Paratypes***CUMZ 7507 (four shells and six specimens preserved in ethanol; Fig. 25F, width 26.4 mm, height 12.0 mm), CUMZ 7514 (11 specimens preserved in ethanol), NHMUK 20200284 (two shells).

###### Other material examined.

**Thailand-Central.** Tham Khao Wong, Ban Rai, Uthai Thani, 15°01'53.4"N, 99°27'21.1"E: CUMZ 7508. Khao Chong Lom, Ban Rai, Uthai Thani, 15°16'51.6"N, 99°43'08.4"E: CUMZ 7509. Wat Khao Chueak Charoen Tham, Ban Rai, Uthai Thani, 15°16'17.2"N, 99°41'43.6"E: CUMZ 7515. Hup Pa Tat, Lan Sak, Uthai Thani, 15°22'33.8"N, 99°37'49.5"E: CUMZ 7510, 7511, 7512, 7513. Wat Thep Muang Thong, Lan Sak, Uthai Thani, 15°24'59.5"N, 99°35'36.6"E: CUMZ 7516.

###### Type locality.

Tham Namthip Bureau of Monks, Lan Sak, Uthai Thani, Thailand, 15°25'57.5"N, 99°35'19.6"E.

###### Diagnosis.

Shell large, strongly depressed and pale yellowish brown to pale brown with very rounded body whorl. Animal with grey body and five mantle lobes. Genitalia with a straight epiphallic caecum and triangular prism pilasters on inner penial sculpture. Spermatophore: head filament with irregularly smooth longitudinal ridges; tail filament near sperm sac with three spines and terminal part of tail filament more than more than ca. one-fourth of its length with series of branching spines.

###### Description.

***Shell*.** Shell strongly depressed, large size (shell width up to 26.4 mm, shell height up to 12.4 mm), and thin. Surface smooth and glossy; shell colour pale yellowish brown to pale brown. Whorls 6–6½, increasing regularly; body whorl large and well rounded. Spire slightly elevated; suture impressed. Aperture crescent-shaped and opening obliquely. Peristome simple. Columellar margin simple and slightly reflected near umbilicus. Umbilicus narrowly opened (Fig. 25E, F).

**Figure 32.**
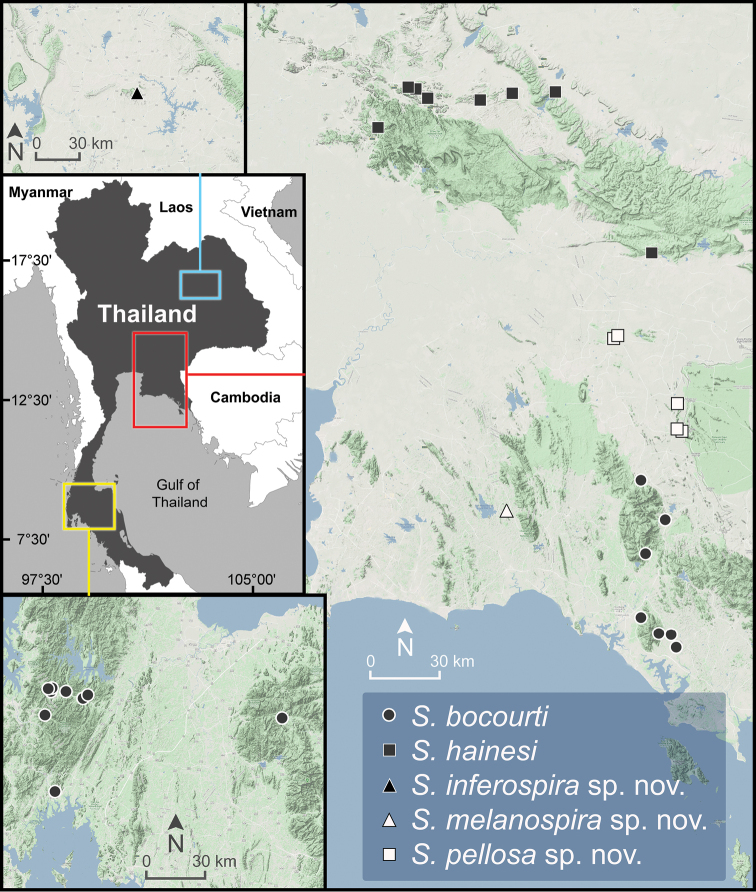
Geographic distribution of *Sarika bocourti*, *S. hainesi*, *S. inferospira* sp. nov., *S. melanospira* sp. nov. and *S. pellosa* sp. nov. based on examined specimens herein.

***Genital organs*.** Atrium short. Penis cylindrical with thin penial sheath covering proximal penis. Inner sculpture of penis proximally more than ca. one-third of penial chamber with very finely longitudinal penial pilasters to nearly smooth surface, and then gradually transformed from small to large rhomboid with acute angle on top (triangular prism). Epiphallus cylindrical, long and approximately one and half times as long as penis. Epiphallic caecum short, straight, and located proximally near middle of epiphallus, penial retractor muscle thin and attached at tip of epiphallic caecum. Flagellum long and slender, approximately as long as penis. Vas deferens thin tube connecting distal epiphallus and free oviduct (Fig. 28).

**Figure 33.**
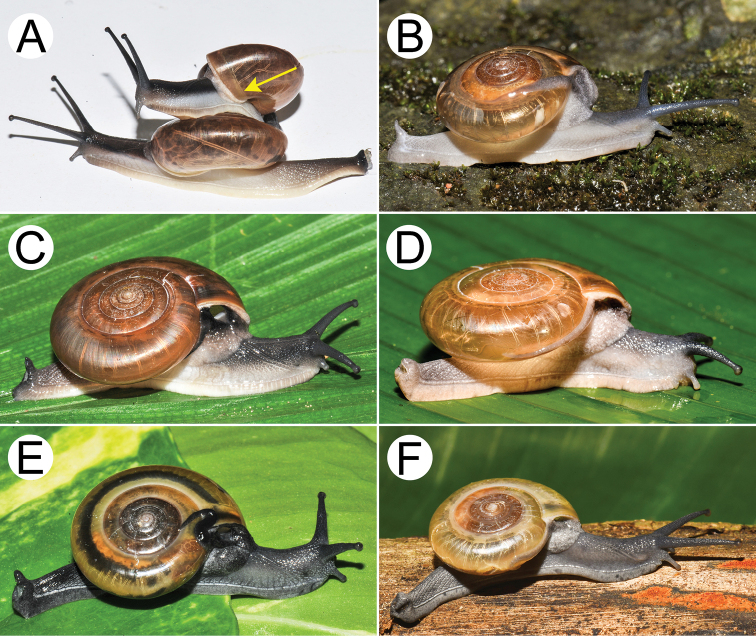
Living snails of group II: *Sarika hainesi* group. **A***Sarika bocourti* specimen CUMZ 7591 showing no left shell lobe (yellow arrow) **B***S. hainesi* specimen CUMZ 7237 **C***S. bocourti* specimen CUMZ 7591 **D***S. inferospira* sp. nov. specimen CUMZ 7257 **E***S. melanospira* sp. nov. paratype CUMZ 7243, and **F***S. pellosa* sp. nov. paratype CUMZ 7517. Not to scale.

Vagina cylindrical and approximately as long as penis. Dart apparatus large, long cylindrical, located on atrium at vagina and penis junction. Gametolytic sac enlarged and bulbous; gametolytic duct long, cylindrical. Free oviduct cylindrical, longer than vagina and proximal end encircled with thick tissue (Fig. 28A).

Spermatophore long and needle-shaped. Sperm sac enlarged and elongate-oval. Head filament gourd shape with irregularly smooth longitudinal ridges. Tail filament very long tube; region near sperm sac with three spines. Spine I simple and rather short. Spine II large and long and branching into very small spinules. Spine III relatively smaller than spine II and branching into very small spinules. Region furthest away smooth and without spine; terminal part (more than ca. one-fourth of its length) with series of long branching spines into spinules arranged in a row, and then transformed to very long serrate-like spines arranged in opposite rows near the tail filament tip (Fig. 29).

**Figure 34.**
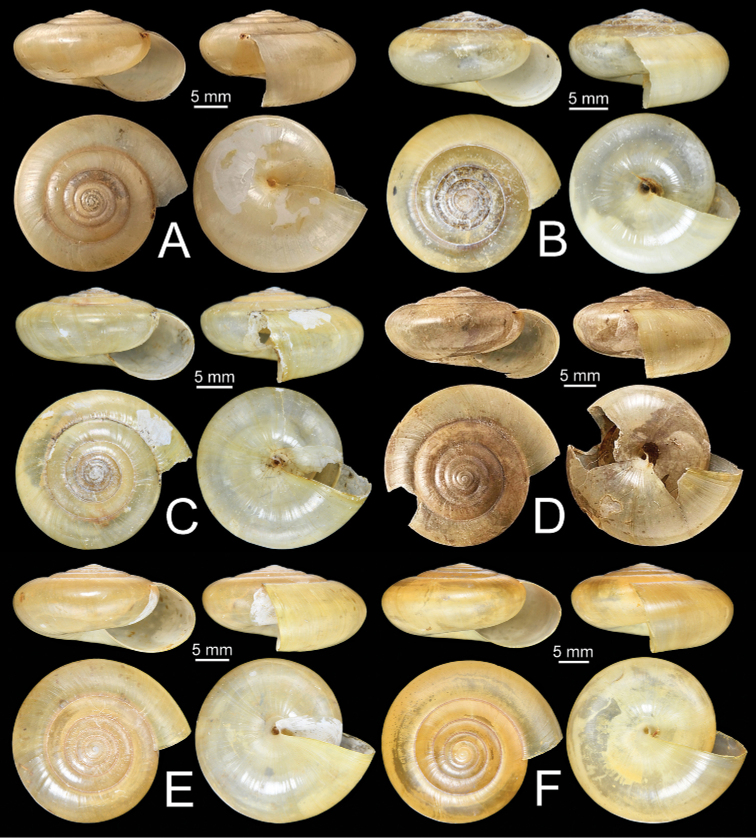
Shells of Group II: *Sarika hainesi* group. **A–C***Sarika hainesi***A** syntype NHMUK 20200290 and **B, C** specimen CUMZ 7237 **D–F***S. bocourti***D** syntype NHMUK 1893.2.4.1076–1077 **E** specimen CUMZ 7590, and **F** specimen CUMZ 7592.

***Radula*.** Teeth with half row formula: 1–(14–15)–68. Central tooth symmetrical tricuspid; lateral teeth asymmetrical tricuspid; marginal teeth elongate bicuspid. Marginal teeth starting at approximately row number 14 or 15 (Fig. 31E).

***External features*.** Animal with reticulated skin and pale to dark grey body. Foot sole and caudal foss present; caudal horn raised. Five mantle lobes well developed and same colour as body (Fig. 10F).

###### Etymology.

The specific name *subheptagyra* is derived from the Latin word *sub* meaning under, from, somewhat, and less than, and the word *heptagyra* referring to shell similar to *S. heptagyra*.

###### Distribution.

This species occurs only in Uthai Thani Province and is restricted to limestone habitats (Fig. 6).

**Figure 35.**
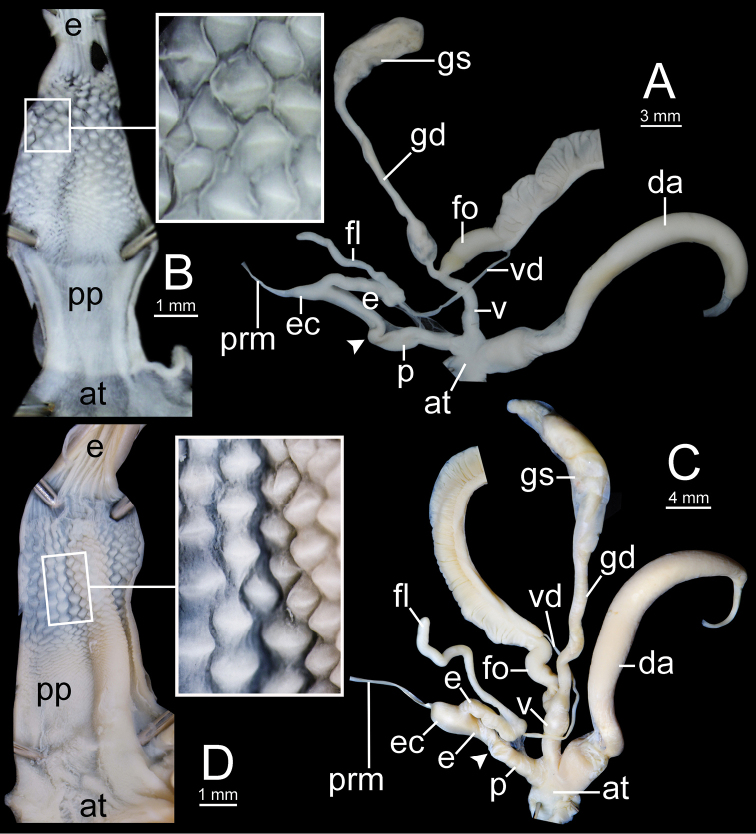
Genitalia. **A, B***Sarika hainesi* specimen CUMZ 7237 **A** general view of the genital system and **B** internal structure of the penis. **C, D***Sarika bocourti* specimen CUMZ 7591 **C** general view of the genital system and **D** internal structure of the penis. White arrowheads indicate the ends of the penes.

###### COI analysis.

The ML and BI analyses indicated that the samples of *S. subheptagyra* sp. nov. (n = 3) formed a monophyletic group with good support (Fig. 1; BS = 90%, PP = 1). The mean intraspecific genetic distance of *S. subheptagyra* sp. nov. was 2.4% (Table 2).

###### Remarks.

*Sarika subheptagyra* sp. nov. differs from *S. heptagyra* in having longer vagina and free oviduct, and triangular prism-shaped penial pilasters, while *S. heptagyra* have cuboidal penial pilasters. Moreover, the COI sequence divergences between them is high (7.9%).

Compared with *S. resplendens*, *S. subheptagyra* sp. nov. has a longer vagina and free oviduct, thin penial retractor muscle, and triangular prism-shaped penial pilasters. *Sarika resplendens* has a shorter vagina and free oviduct, a very large and thickened penial retractor muscle, and cuboidal-shaped penial pilasters. Additionally, the genetic distance between both species is fairly high (6.5%).

*Sarika subheptagyra* sp. nov. differs from *S. caligina* sp. nov. in having a lower spire, longer vagina, and free oviduct, and spine II and spine III on spermatophore start branching near the base, while *S. caligina* sp. nov. has a higher spire, shorter vagina and free oviduct, and spine II and spine III on spermatophore start branching near the tip. In addition, the genetic distance between these two new species is high (8.2%).

**Figure 36.**
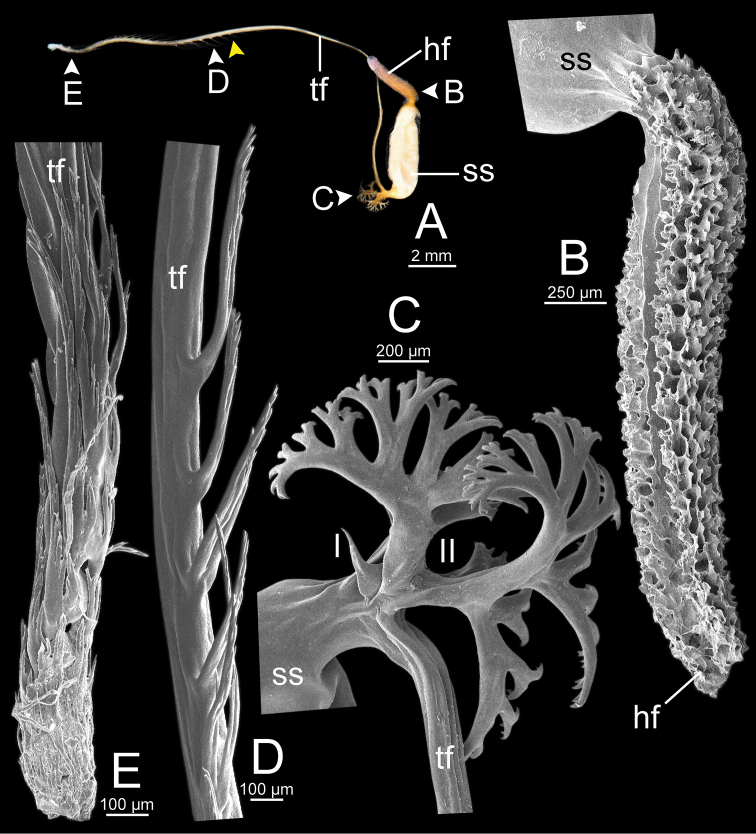
Spermatophore of *Sarika hainesi* specimen CUMZ 7237 **A** general view of the spermatophore **B** head filament, and **C–E** tail filament showing **C** two spines located close to the sperm sac **D** region with and without branching spines, and **E** branching spines on the tip region. Yellow arrowhead indicates the end of the spines from the tip.

#### Group II: *Sarika hainesi* group: species without left shell lobe and without penial verge or pseudo-verge

##### Sarika hainesi

Taxon classificationAnimaliaStylommatophoraAriophantidae

(Pfeiffer, 1856)

CB1888D7-1C5E-5BDC-8570-B450FE8EAF7B

Figs 1 , 32 , 33B , 34A–C , 35A, B , 36 , 43A

Helix hainesi Pfeiffer, 1856a: 32. Type locality: “Siam” [Thailand]; Pfeiffer 1856b: 75, 76, pl. 21, figs 1–3.
Nanina ﻿ (Orobia) hainesi: Martens 1867: 73; Pfeiffer 1868: 122.
Nanina (Macrochlamys) hainesi : Tryon 1886: 96, pl. 32, figs 36–38; Fischer and Dautzenberg 1904: 395.
Ariophanta (Macrochlamys) hainesi : Fischer 1891: 20.
Macrochlamys hainesi : Saurin 1953: 113.
Sarika hainesii [sic]: Hemmen and Hemmen 2001: 45.
Sarika hainesi : Inkhavilay et al. 2019: 82, fig. 38f.

###### Type mater﻿﻿ial.

***Syntype***NHMUK ex. Cuming collection: 20200290 (three shells; Fig. 34A) from Siam [Thailand].

###### Other material examined.

**Thailand-Northeastern.** Wat Tham Khao Wong, Pak Chong, Nakhon Ratchasima, 14°35'15.5"N, 101°20'33.5"E: CUMZ 7273. Wat Tham Thep Nimit, Pak Chong, Nakhon Ratchasima, 14°34'18.7"N, 101°33'38.5"E: CUMZ 7268. Wat Tham Thian Chai Chonprathan, Pak Chong, Nakhon Ratchasima, 14°36'58.5"N, 101°18'35.3"E: CUMZ 7270. Wat Thep Phithak Punnaram, Pak Chong, Nakhon Ratchasima, 14°36'58.5"N, 101°15'55.9"E: CUMZ 7271. Lam Phra Phloeng area, Pak Thong Chai, Nakhon Ratchasima, 14°35'44.0"N, 101°50'07.4"E: CUMZ 7274. Wat Tham Praput, Pak Chong, Nakhon Ratchasima, 14°35'37.5"N, 101°40'17.8"E: CUMZ 7269. **Thailand-Eastern.** Pang Sida Waterfall, Watthana Nakhon, Sa Kaeo, 13°59'36.4"N, 102°12'21.2"E: CUMZ 7237 (Fig. 34B, C), 7267. **Thailand-Central.** Ched Khot Waterfall, Kaeng Khoi, Saraburi, 14°28'22.3"N, 101°10'01.7"E: CUMZ 7272.

###### Diagnosis.

Shell large, depressed, obtusely angulated body whorl. Animal with pale grey body and four mantle lobes. Genitalia with a large and straight epiphallic caecum, and a triangular prism shape of penial pilasters. Spermatophore with irregularly obtuse-serrate longitudinal ridges with numerous pores on the head filament, tail filament with two spines and more than ca. half of its length with series of long branching spines.

**Figure 37.**
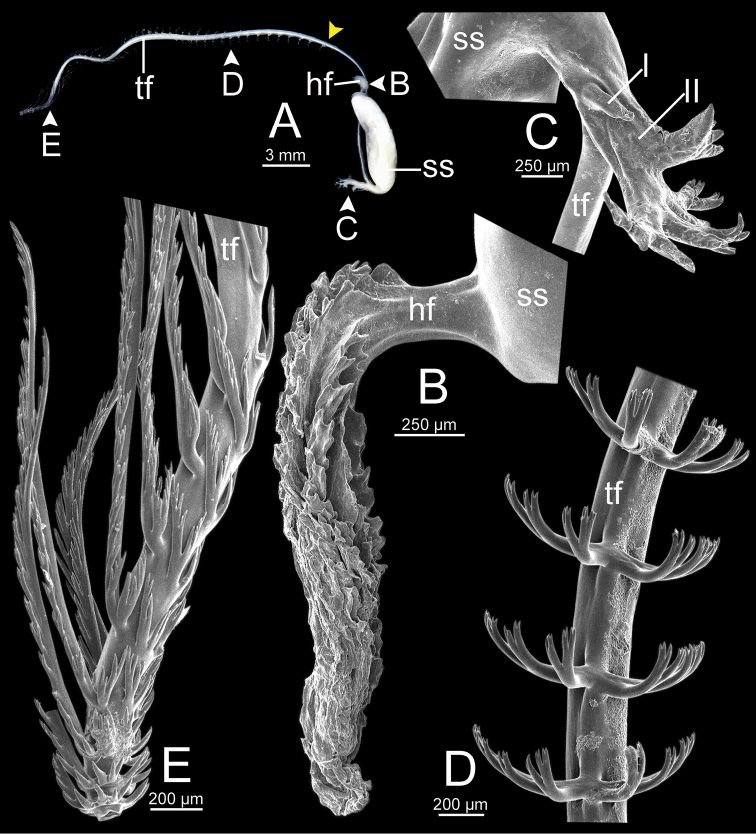
Spermatophore of *Sarika bocourti* specimen CUMZ 7591 **A** general view of the spermatophore **B** head filament, and **C–E** tail filament showing **C** two spines located close to the sperm sac **D** branching spines on middle region **E** branching spines on the tip region. Yellow arrowhead indicates the end of the spines from the tip.

###### Description.

***Shell*.** Shell depressed, large size (shell width up to 28.1 mm, shell height up to 14.9 mm) and rather thin. Shell surface smooth and polished; shell colour pale yellowish brown to pale brown. Whorls 6–7, size increasing regularly; body whorl large and obtusely angled. Spire moderately elevated; suture impressed. Aperture crescent-shaped and obliquely opened. Peristome simple. Columellar margin simple and slightly reflected near umbilicus. Umbilicus narrowly opened (Fig. 34A–C).

***Genital organs*.** Atrium short. Penis cylindrical with thin penial sheath covering proximal penis. Inner sculpture of penis proximally more than ca. half of penial chamber with very finely longitudinal penial pilasters to nearly smooth surface, and then gradually transformed from small to large rhomboid pilasters with acute angle on top (triangular prism). Epiphallus cylindrical, approximately one and half times total penis length, and narrower than penis. Epiphallic caecum short, straight, same diameter as proximal epiphallus, and located near middle of epiphallus. Penial retractor muscle thin and attached at tip of epiphallic caecum. Flagellum long slender and almost as long as penis. Vas deferens thin tube connecting distal epiphallus and free oviduct (Fig. 35A, B).

Vagina cylindrical and slightly shorter than penis. Dart apparatus enlarged, long cylindrical and located on atrium at vagina and penis junction. Gametolytic sac enlarged and bulbous (with spermatophore inside); gametolytic duct long and cylindrical. Free oviduct cylindrical, approximately as long as vagina, and proximal end encircled with thick tissue (Fig. 35A).

Spermatophore long and needle-shaped. Sperm sac enlarged and elongate-oval. Head filament gourd shape and irregularly obtuse-serrate longitudinal ridges with numerous pores (sponge-like). Tail filament very long tube; region near sperm sac with two spines. Spine I located on same base with spine II, short and simple. Spine II large, long and very complicated branching into many spinules. Region furthest away smooth and without spine; terminal part (more than ca. half of its length) with series of long branching spines arranged in an arrow or encircled tail filament tip (Fig. 36).

***Radula*.** Teeth with half row formula: 1–(10–11)–61. Central tooth symmetrically tricuspid; lateral teeth asymmetrically tricuspid; marginal teeth elongated and bicuspid. Marginal teeth starting at approximately row number 10 or 11 (Fig. 43A).

***External features*.** Animal with reticulated skin and pale grey body. Mantle edge well developed, pale grey, with one shell lobe, and three dorsal lobes. Dorsal lobes large and broad; anterior and posterior left dorsal lobes smaller than right dorsal lobe. Right shell lobe large and long and left shell lobe absent (Fig. 33B).

###### Distribution.

This species is known only from the Dong Phaya Yen and Sankamphaeng Ranges in Saraburi and Nakhon Ratchasima provinces (Fig. 32).

###### COI analysis.

The ML and BI analyses revealed that the individuals of *S. hainesi* (n = 3) formed a monophyletic group with high support (Fig. 1; BS = 96%, PP = 1). The mean intraspecific genetic distance of *S. hainesi* was 2.4% (Table 2).

###### Remarks.

The type locality of *S. hainesi* was recorded simply as “Siam”. Later, Pfeiffer (1856b: 76) stated that the described specimens were received from W.A. Haines. Although Haines had never visited Thailand, he described four land snail species from Thailand based on materials sent by the American physician S.R. House (Haines 1858: 157–158). We presumed that the Cuming ex. Haines specimen (syntype) was also received from S.R. House who lived in Bangkok from 1847–1876 (Feltus 1924). Therefore, the type locality of *S. hainesi* from central Thailand is the most likely. In addition, the specimen from central Thailand (*S. hainesi* s. s.) matched the syntypes in almost all the shell characters; however, the genitalia obviously differ from S. aff. hainesii sensu Solem (1966) from northern Thailand (Chiang Mai Province). We consider the northern Thailand populations as misidentified and propose them here as an undescribed species, *S. solemi* sp. nov. Moreover, the COI sequence divergences between *S. hainesi* and *S. solemi* sp. nov. are very high (10.2%).

*Sarika hainesi* was first reported from Thailand (Pfeiffer 1856a) and then from Laos (Saurin 1953; Inkhavilay et al. 2019). However, the specimens from Laos are shells only, and so the distribution of *S. hainesi* s. s. in Laos still needs to be confirmed.

##### Sarika bocourti

Taxon classificationAnimaliaStylommatophoraAriophantidae

(Morelet, 1875)

5DA81666-0D92-5541-9FFE-843019482F33

Figs 1 , 32 , 33A, C , 34D–F , 35C, D , 37 , 43B

Helix bocourti Morelet, 1875: 249. Type locality: “Ľespece provient de Battambang, dans le Cambodje” [Battambang Province, Cambodia]; Breure et al. 2018: 222, 223, figs 135, 136.
Nanina (Macrochlamys) aff. boucourti [sic]: Tryon 1886: 89, pl. 29, figs 43–45.
Ariophanta (Xesta) bocourti : Fischer 1891: 20.
Nanina (Xesta) bocourti : Fischer and Dautzenberg 1904: 394.
Sarika bocourti : Pholyotha et al. 2020a: 7, 8, fig. 2b; Sutcharit et al. 2020: 27.

###### Type material.

***Syntype***NHMUK 1893.2.4.1076–1077 (two shells; Fig. 34D) from Battambang [Battambang Province, Cambodia].

###### Other material examined.

**Cambodia**. Samov Mountain, Phnom Sampov, Banan, Battambang, 13°01'33.6"N, 103°06'03.6"E: CUMZ 7900. **Thailand-Eastern.** Wat Trok Nong Lang, Khlung, Chanthaburi, 12°32'16.3"N, 102°16'33.8"E: CUMZ 7578. Trok Nong Waterfall, Khlung, Chanthaburi, 12°32'39.4"N, 102°14'13.5"E: CUMZ 7580, 7582, 7583, 7585, 7590 (Fig. 34E), 7591, 7596, 7608. Khao Kaeo Priest’s camp site, Khlung, Chanthaburi, 12°29'26.8"N, 102°18'49.6"E: CUMZ 7598. Khlong Narai Waterfall, Mueang, Chanthaburi, 12°34'53.4"N, 102°10'34.2"E: CUMZ 7597, 7601, 7610. Wat Khao Banchob, Makham, Chanthaburi, 12°51'09.1"N, 102°12'12.7"E: CUMZ 7581. Khao Soi Dao, Soi Dao, Chanthaburi, 13°06'31.0"N, 102°12'01.5"E: CUMZ 7579, 7584, 7586, 7609. Mountain area near Khao Soi Dao, Soi Dao, Chanthaburi, 13°07'49.3"N, 102°10'57.2"E: CUMZ 7600. Mountain area near Wat Ban Wang Ka Prae, Pong Nam Ron, Chanthaburi, 12°58'10.9"N, 102°16'20.6"E: CUMZ 7594. **Thailand-Southern.** Mountain area near Khao Sok Nature Resort, Phanom, Surat Thani, 8°54'22.6"N, 98°31'45.1"E: CUMZ 7587. Mountain area near Khao Sok Evergreen House, Phanom, Surat Thani, 8°54'38.1"N, 98°31'47.2"E: CUMZ 7592 (Fig. 34F), 7606. Mountain area near Anurak Community Lodge, Phanom, Surat Thani, 8°53'16.2"N, 98°40'52.9"E: CUMZ 7593, 7603. Wat Tham Wararam, Phanom, Surat Thani, 8°53'03.3"N, 98°40'02.5"E: CUMZ 7604. Wat Tham Phanthurat, Phanom, Surat Thani, 8°54'36.9"N, 98°31'28.3"E: CUMZ 7595. Mountain area near Ban Ya Plong, Phanom, Surat Thani, 8°54'33.0"N, 98°34'51.6"E: CUMZ 7605. Lot cave, Nopphitam, Nakhon Si Thammarat, 8°44'10.0"N, 99°38'06.5"E: CUMZ 7602. Limestone outcrop in Tham Nam Phut, Mueang, Phang-nga, 8°27'49.7"N, 98°32'37.0"E: CUMZ 7607. Mountain area near Ban Pak Khlong, Kapong, Phang-nga, 8°47'54.8"N, 98°30'40.6"E: CUMZ 7588.

###### Diagnosis.

Shell large, depressed, obtusely angulated body whorl and higher shell spire. Animal with pale to dark grey body and four mantle lobes. Genitalia with a large and straight epiphallic caecum, and triangular prism shaped penial pilasters. Spermatophore with irregularly acute-serrate longitudinal ridges on the head filament, tail filament with two spines and more than ca. two-thirds of its length with series of short branching spines.

**Figure 38.**
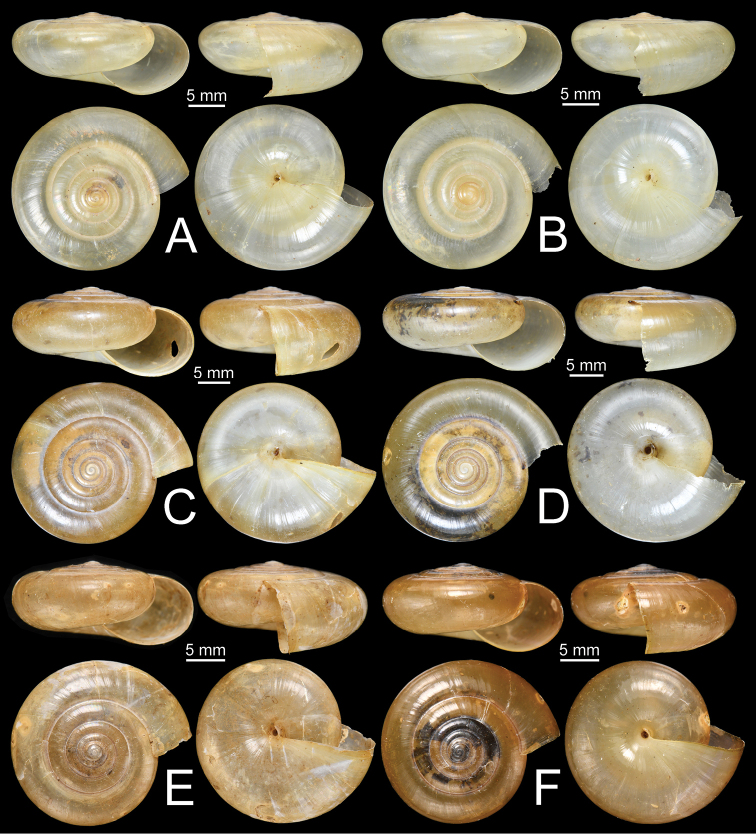
Shells of Group II: *Sarika hainesi* group. **A, B***Sarika inferospira* sp. nov. **A** holotype CUMZ 7254 and **B** paratype CUMZ 7255 **C, D***S. melanospira* sp. nov. **C** holotype CUMZ 7258 and **D** paratype CUMZ 7243 **E, F***S. pellosa* sp. nov. **E** holotype CUMZ 7249 and **F** paratype CUMZ 7517.

###### Description.

***Shell*.** Shell depressed, large to very large size (shell width up to 33.1 mm, shell height up to 16.1 mm) and rather thin. Shell surface smooth and polished; shell colour pale yellowish brown to brown. Whorls 6–7, increasing regularly; body whorl large and obtusely angulated. Spire moderately to very much elevated; suture impressed. Aperture crescent-shaped and obliquely opened. Peristome simple. Columellar margin simple and slightly reflected near umbilicus. Umbilicus narrowly opened (Fig. 34D–F).

**Figure 39.**
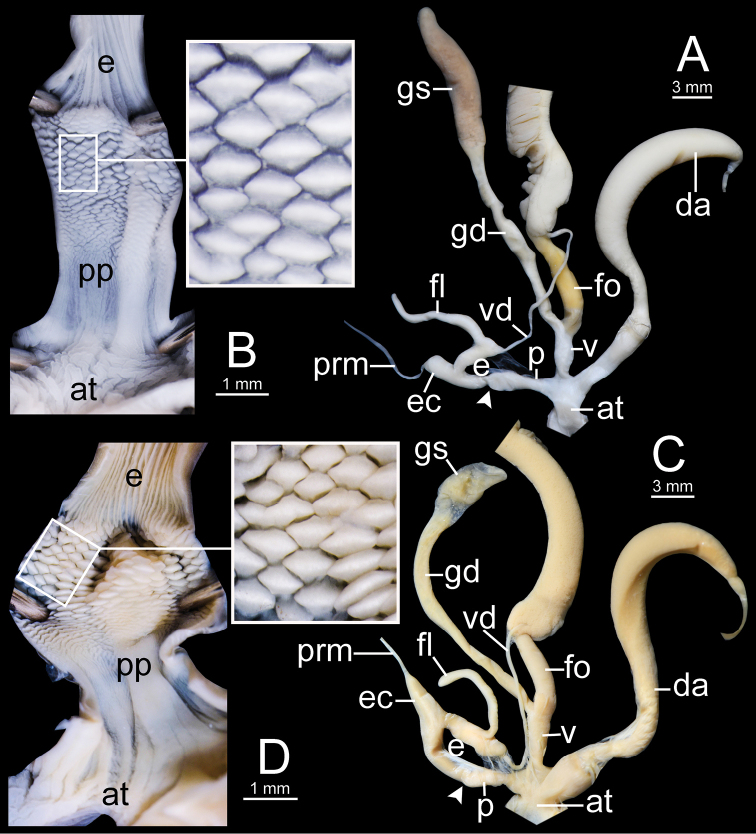
Genitalia. **A, B***Sarika inferospira* sp. nov. specimen CUMZ 7257 **A** general view of the genital system and **B** internal structure of the penis **C, D***S. melanospira* sp. nov. paratype CUMZ 7243 **C** general view of the genital system and **D** internal structure of the penis. White arrowheads indicate the ends of the penes.

***Genital organs*.** Atrium short. Penis cylindrical with thin penial sheath covering proximal penis. Inner sculpture of penis proximally more than ca. half of penial chamber with very finely longitudinal penial pilasters to nearly smooth surface, and then gradually transformed from small to large rhomboid pilasters with acute angle on top (triangular prism). Epiphallus cylindrical, slightly longer than penis, and approximately same diameter as penis. Epiphallic caecum short, straight, approximately similar diameter with penis, and located near middle of epiphallus. Penial retractor muscle thin and attached at tip of epiphallic caecum. Flagellum long slender and slightly longer than epiphallus. Vas deferens thin tube connecting distal epiphallus and free oviduct (Fig. 35C, D).

Vagina cylindrical and approximately as long as penis. Dart apparatus enlarged, long cylindrical, and located on atrium at vagina and penis junction. Gametolytic sac enlarged and bulbous (with spermatophore inside); gametolytic duct cylindrical. Free oviduct cylindrical, nearly two times of vagina length, and proximal end encircled with thick tissue (Fig. 35C).

Spermatophore long and needle-shaped. Sperm sac enlarged and elongate-oval. Head filament gourd shape with irregularly acute-serrate longitudinal ridges. Tail filament very long tube; region near sperm sac with two spines. Spine I simple and rather short. Spine II very large at base and divided in two spines and then each one branching into many spinules near the tip. Region furthest away smooth without spine; terminal part (more than ca. two-thirds of its length) with series of short branching spines arranged in a row and transformed to long serrate-like spines arranged in opposite rows near the tail filament tip (Fig. 37).

***Radula*.** Teeth with half row formula: 1–(12–13)–61. Central tooth symmetrical tricuspid with large mesocone and very small to nearly absent ectocone; lateral teeth asymmetrical tricuspid with large mesocone and very small to nearly absent endocone and ectocone; marginal teeth elongate bicuspid. Marginal teeth starting at approximately row number 12 or 13 (Fig. 43B).

***External features*.** Animal with reticulated skin, pale to dark grey body and darker than foot sole, and dark grey caudal horn. Mantle edge well developed and pale grey colour. Shell lobes and dorsal lobes shape and structure like *S. hainesi* (Fig. 33A, C).

###### Distribution.

This species is known from several localities in Chanthaburi Province, eastern Thailand and Surat Thani, Nakhon Si Thammarat and Phang-nga provinces, southern Thailand (Fig. 32), and Battambang Province, Cambodia (Morelet 1875; Pholyotha et al. 2020a). *Sarika bocourti* is common in both human-influenced habitats, such as plantations or gardens, and natural habitats.

###### COI analysis.

The ML and BI analyses showed that the four specimens of *S. bocourti* represent a single haplotype, sister group to *S. inferospira* sp. nov. + *S. melanospira* sp. nov. with strong support (Fig. 1; BS = 99%, PP = 1).

###### Remarks.

Specimens from Chanthaburi Province, eastern Thailand were identical with the syntype of *S. bocourti* that was described from Battambang Province, Cambodia. Both the shell morphology and genital anatomy of the disjunct populations from southern Thailand agree well with the populations from eastern Thailand. From the COI gene phylogeny, all specimens from southern Thailand are retrieved as monophyletic with *S. bocourti* from eastern Thailand and with no variation in the COI sequences (Table 2). The eastern and southern populations of *S. bocourti* are possibly shaping up by sea level fluctuation in the last glacial periods recorded in tree-dwelling snails and centipedes (Prasankok et al. 2007; Siriwut et al. 2015). Further investigation by adding more samples and genetic markers would help elucidate the phylogeographical history of this species.

Although shell morphology of *S. bocourti* and *S. hainesi* is quite similar, the genitalia and spermatophore are clearly distinct. *Sarika bocourti* has larger epiphallic caecum, and a spermatophore with a head filament with acute-serrate ridges, and the tail filament has fewer branching spines. *Sarika hainesi* has a smaller epiphallic caecum, and a spermatophore with a head filament with a sponge-like appearance and the tail filament has more branching spines. In addition, the genetic distance between these two species is rather high (7.0%).

##### Sarika inferospira

Taxon classificationAnimaliaStylommatophoraAriophantidae

Pholyotha & Panha sp. nov.

081C3931-81B3-5C64-B075-68F6BF10CD2E

http://zoobank.org/CE4EE71E-7FCE-4696-BDEE-A145178CBAE3

**Figure 40.**
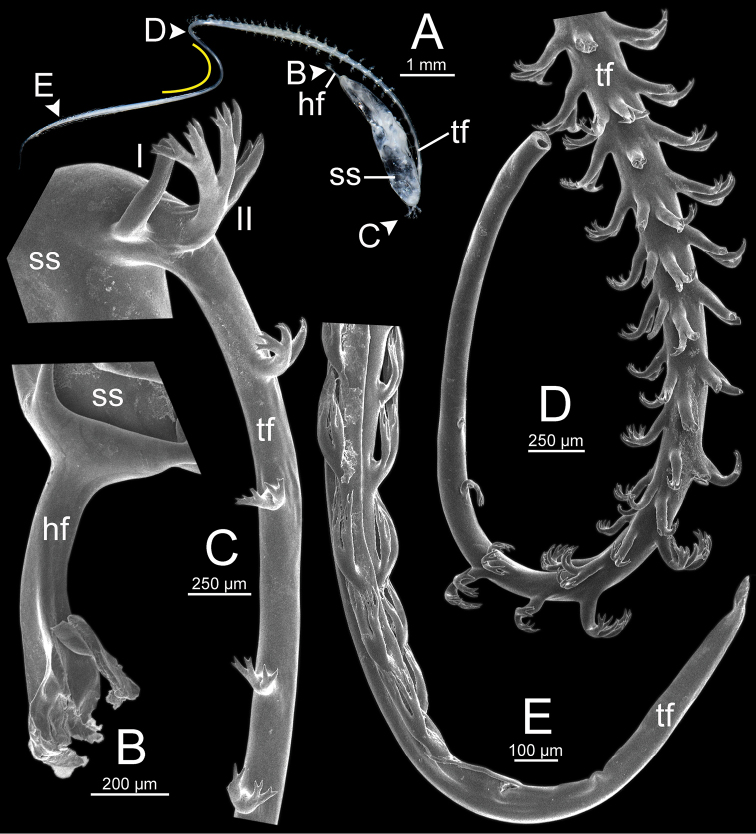
Spermatophore of *Sarika inferospira* sp. nov. specimen CUMZ 7257 **A** general view of spermatophore **B** head filament and **C–E** tail filament showing **C** two spines located close to the sperm sac **D** region with and without branching spines, and **E** branching spines on the tip region. Yellow line indicates the region with no spine.

Figs 1 , 32 , 33D , 38A, B , 39A, B , 40 , 43C

###### Type material.

***Holotype***CUMZ 7254 (Fig. 38A, width 25.3 mm, height 11.5 mm). ***Paratypes***CUMZ 7255 (two shells and 13 specimens preserved in ethanol; Fig. 38B, width 24.8 mm, height 11.0 mm) CUMZ 7256 (four shells), 7257 (four specimens preserved in ethanol), NHMUK 20200285 (two shells).

###### Type locality.

Wat Tham Sai Thong, Nong Kung Si, Kalasin, Thailand, 16°50'11.3”N, 103°14'18.7”E.

###### Diagnosis.

Shell large, strongly depressed, very pale yellowish brown with shouldered body whorl. Animal with grey colour and four mantle lobes. Genitalia with a large straight epiphallic caecum, and triangular prism pilasters on inner penial sculpture. Spermatophore: tail filament near sperm sac with two spines and a series of several branching spines occurring continually to the middle region; middle region becoming smooth, spineless and then terminal part approximately half of its length with a series of branching spines.

###### Description.

***Shell*.** Shell strongly depressed, large size (shell width up to 29.3 mm, shell height up to 13.9 mm) and thin. Surface smooth and polished; shell colour very pale yellowish brown. Whorls 6–6½, increasing regularly; body whorl large and shouldered. Spire slightly elevated; suture impressed. Aperture crescent-shaped and obliquely opened. Peristome simple. Columellar margin simple and slightly reflected near umbilicus. Umbilicus narrowly opened (Fig. 38A, B).

***Genital organs*.** Atrium short. Penis cylindrical with thin penial sheath covering proximal penis. Inner sculpture of penis proximally more than ca. half of penial chamber with very finely longitudinal penial pilasters to nearly smooth surface, and then gradually transformed from small to large rhomboid pilasters with acute angle on top (triangular prism). Epiphallus cylindrical and approximately the as long as penis. Epiphallic caecum short, straight, diameter larger than epiphallus, and located near middle of epiphallus. Penial retractor muscle thin and attached at tip of epiphallic caecum. Flagellum long, slender and nearly one and half times of epiphallus length. Vas deferens thin tube connecting distal epiphallus and free oviduct (Fig. 39A, B).

Vagina cylindrical, short, and approximately two-third of penis length. Dart apparatus large, long cylindrical and located on atrium at vagina and penis junction. Gametolytic sac enlarged and bulbous; gametolytic duct long cylindrical. Free oviduct cylindrical, approximately two and half times of vagina length (Fig. 39A).

Spermatophore long and needle-shaped. Sperm sac enlarged and elongate-oval. Head filament was missing (incomplete spermatophore). Tail filament very long tube and region near sperm sac with two spines. Spine I simple and long. Spine II slightly longer and larger than spine I and branching into many small spinules. Continuously on tail filament with short branching spines arranged in a row, modified to longer branching spines arranged in several rows around middle region, and then become smooth and without spine (Fig. 40A at yellow line). Terminal part of tail filament (more than ca. one-fourth of its length) with series of long branching spines arranged in opposite rows and tail filament tip with no spine (Fig. 40).

***Radula*.** Teeth with half row formula: 1–(13–14)–59. Central tooth symmetrical tricuspid; lateral teeth asymmetrical tricuspid; marginal teeth elongate bicuspid. Teeth shape is similar to that of *S. resplendens*. Marginal teeth starting at approximately row number 13 or 14 (Fig. 43C).

***External features*.** Animal with reticulated skin and body colour with dark grey above and creamy-grey below. Creamy-grey foot sole and dark creamy-grey caudal horn. Four mantle lobes well developed and pale grey colour. Left shell lobe absent (Fig. 33D).

###### Etymology.

The specific epithet *inferospira* is derived from the Latin word *infer* meaning low and the Latin word *spira* meaning coil. It refers to the strongly depressed shell with low spire.

###### Distribution.

*Sarika inferospira* sp. nov. is only known from sandstone habitats with dry dipterocarp forest at the type locality (Fig. 32).

###### COI analysis.

The ML and BI analyses revealed that the individuals of *S. inferospira* sp. nov. (n = 3) formed a monophyletic group with very strong support (Fig. 1; BS = 100%, PP = 1), sister group to *S. melanospira* sp. nov. with only ML support (Fig. 1; BS = 78%). The mean intraspecific genetic distance of *S. inferospira* sp. nov. was 0.1% (Table 2).

###### Remarks.

*Sarika inferospira* sp. nov. is distinguished from *S. hainesi* and *S. bocourti* by having a strongly depressed shape, shouldered body whorl, and spermatophore smooth or without spine on the middle part of tail filament. *Sarika hainesi* and *S. bocourti* have a depressed shell with a higher spire and obtusely angulated body whorl. In addition, the tail filament of spermatophore contains a series of short branching spines more than ca. half of its length in *S. hainesi* and more than ca. two-thirds of its length in *S. bocourti*.

##### Sarika melanospira

Taxon classificationAnimaliaStylommatophoraAriophantidae

Pholyotha & Panha sp. nov.

AC2C1933-B340-5B6F-9813-44163F88475E

http://zoobank.org/EB33505F-3BA4-440C-BBC4-2B4CE631F7AA

Figs 1 , 32 , 33E , 38C, D , 39C, D , 43D

###### Type material.

***Holotype***CUMZ 7258 (Fig. 38C, width 28.3 mm, height 13.3 mm). ***Paratypes***CUMZ 7243 (ten shells and 44 preserved specimens in ethanol; Fig. 38D, width 26.5 mm, height 11.7 mm) CUMZ 7244 (four shells and two preserved specimens in ethanol), NHMUK 20200286 (two shells), SMF (two shells), ZRC.MOL.017029 (two shells).

###### Type locality.

Wat Tham Suwan Phu Pha, Khao Chamao, Rayong, Thailand, 12°59'24.1"N, 101°39'28.8"E.

###### Diagnosis.

Shell large, dextral, depressed and pale brown with rounded to weak shouldered body whorl. Animal with blackish body, four mantle lobes and mantle covered by spiral black band below the suture at the body whorl. Genitalia with a large straight epiphallic caecum and triangular prism pilasters on inner penial sculpture.

###### Description.

***Shell*.** Shell depressed, large size (shell width up to 29.3 mm, shell height up to 13.3 mm) and thin. Surface smooth and polished; shell colour pale brown. Whorls 6–6½, increasing regularly; body whorl large, rounded to weak shouldered. Spire slightly to moderately elevated; suture impressed. Aperture crescent-shaped and obliquely opened. Peristome simple. Columellar margin simple and slightly reflected near umbilicus. Umbilicus narrowly opened (Fig. 38C, D).

***Genital organs*.** Atrium short. Penis cylindrical with thin penial sheath covering proximal penis. Inner sculpture of penis proximally more than ca. half of penial chamber with very finely longitudinal penial pilasters to nearly smooth surface, and then gradually transformed from small to large rhomboid pilasters with acute angle on top (triangular prism). Epiphallus cylindrical and nearly two times penis length. Epiphallic caecum short, straight, slightly larger than epiphallus and located near middle of epiphallus. Penial retractor muscle thin and attached at tip of epiphallic caecum. Flagellum slender, narrower than epiphallus and approximately as long as penis. Vas deferens thin tube connecting distal epiphallus and free oviduct (Fig. 39C, D).

Vagina long cylindrical and approximately as long as penis. Dart apparatus large, long cylindrical, and located on atrium at vagina and penis junction. Gametolytic sac enlarged and bulbous; gametolytic duct long cylindrical. Free oviduct cylindrical, nearly as long as vagina (Fig. 39C).

***Radula*.** Teeth with half row formula: 1–(17–18)–59. Central tooth symmetrical tricuspid; lateral teeth asymmetrical tricuspid; marginal teeth elongate bicuspid. Teeth shape is similar to that of *S. resplendens*. Marginal teeth starting at approximately row number 17 or 18 (Fig. 43D).

***External features*.** Animal with reticulated skin and blackish body. Mantle with conspicuous blackish spiral band at the body whorl below the suture. Creamy-grey foot sole and blackish caudal horn. Four mantle lobes well developed and blackish. Left shell lobe absent (Fig. 33E).

###### Etymology.

The specific epithet *melanospira* is derived from the Greek word *melanos* meaning black or dark, and the Latin word *spira* meaning coil. It refers to the mantle being covered by a spiral black band at the body whorl.

###### Distribution.

*Sarika melanospira* sp. nov. is only known from the limestone habitats at the type locality (Fig. 32).

###### COI analysis.

The ML and BI analyses showed that the individuals of *S. melanospira* sp. nov. (n = 3) formed a monophyletic group with very strong support (Fig. 1; BS = 100%, PP = 1). The mean intraspecific genetic distance of *S. melanospira* sp. nov. was 0.5% (Table 2).

###### Remarks.

Among the *Sarika hainesi* group, this new species differs from *S. hainesi*, *S. bocourti*, and *S. inferospira* sp. nov. in having a rounded to very weak shouldered body whorl. *Sarika hainesi* and *S. bocourti* have an obtusely angulated body whorl and *S. inferospira* sp. nov. has a shouldered body whorl.

##### Sarika pellosa

Taxon classificationAnimaliaStylommatophoraAriophantidae

Pholyotha & Panha sp. nov.

226AB24B-8933-5C64-89AB-15823EC0ABF7

http://zoobank.org/BDA78F68-B700-42D9-8710-30E461EEC205

**Figure 41.**
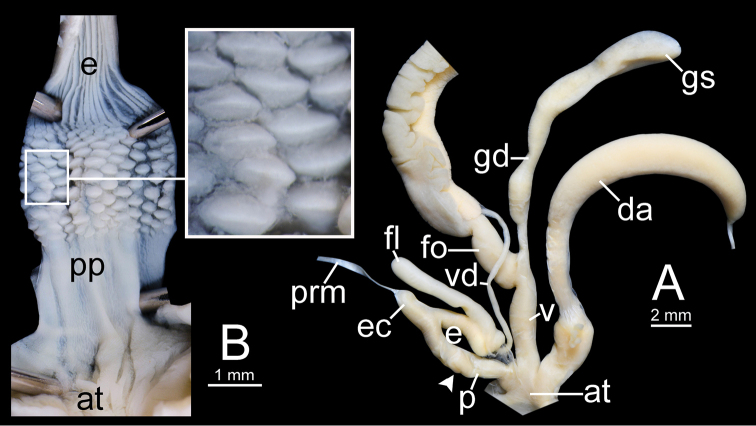
Genitalia. **A, B***Sarika pellosa* sp. nov. paratype CUMZ 7517 **A** general view of the genital system and **B** internal structure of the penis. White arrowhead indicates the end of the penis.

**Figure 42.**
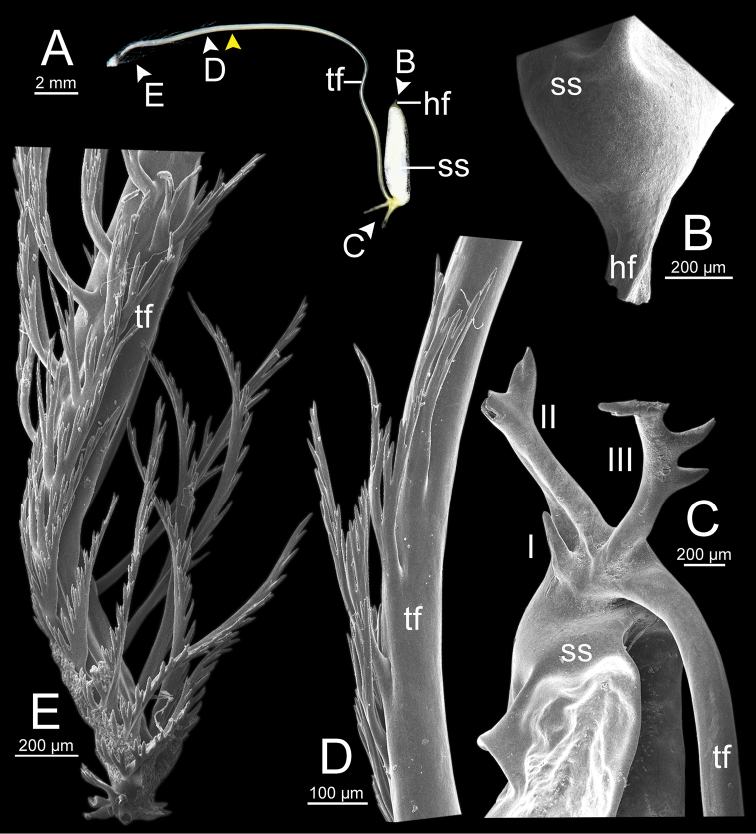
Spermatophore of *Sarika pellosa* sp. nov. paratype CUMZ 7517 **A** general view of the spermatophore **B** head filament, and **C–E** tail filament showing **C** three spines located close to the sperm sac, **D** region with and without branching spines, and **E** branching spines on the tip region. Yellow arrowhead indicates the end of the spines from the tip.

**Figure 43.**
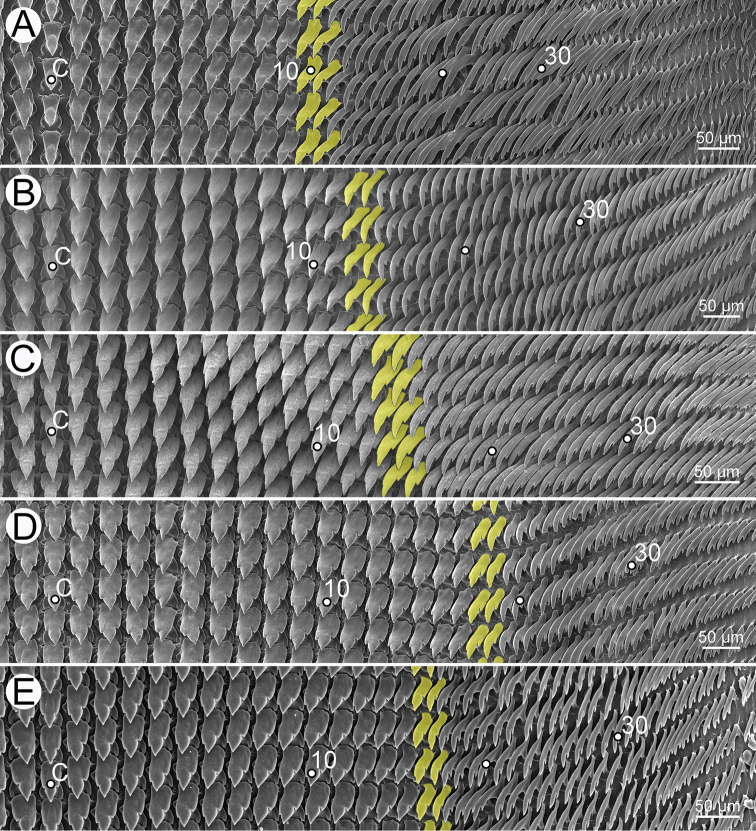
Representative SEM images of the radula. **A***Sarika hainesi* specimen CUMZ 7237 **B***S. bocourti* specimen CUMZ 7597 **C***S. inferospira* sp. nov. paratype CUMZ 7255 **D***S. melanospira* sp. nov. paratype CUMZ 7243, and **E***S. pellosa* sp. nov. specimen CUMZ 7251. Central tooth indicated by ‘C’; Yellow colour indicates lateral teeth with the transition to marginal teeth.

Figs 1 , 32 , 33F , 38E, F , 41 , 42 , 43E

###### Type material.

***Holotype***CUMZ 7249 (Fig. 38E, width 23.6 mm, height 11.0 mm). ***Paratypes***CUMZ 7517 (two shell and four specimens preserved in ethanol; Fig. 38F, width 23.6 mm, height 11.5 mm) CUMZ 7519 (three specimens preserved in ethanol), NHMUK 20200287 (two shells).

###### Other material examined.

**Thailand-Eastern.** Tham Saeng Thian, Khlong Hat, Sa Kaeo, 13°18'57.2"N, 102°19'57.2"E: CUMZ 7250. Tham Nam Khao Siva, Khlong Hat, Sa Kaeo, 13°19'15.1"N, 102°19'40.1"E: CUMZ 7251. Limestone outcrop in Khao Chakan, Khao Chakan, Sa Kaeo, 13°39'49.4"N, 102°05'39.7"E: CUMZ 7518. Wat Tham Khao Chakan, Khao Chakan, Sa Kaeo, 13°39'38.0"N, 102°05'02.8"E: CUMZ 7520.

###### Type locality.

Tham Phet Pho Thong, Khlong Hat, Sa Kaeo, Thailand, 13°25'02.5"N, 102°19'25.6"E.

###### Diagnosis.

Shell large, depressed to strongly depressed, pale brown to dark brown with rounded to weak shouldered body whorl. Animal with blackish body and four mantle lobes. Genitalia with a large straight epiphallic caecum and triangular prism pilasters on inner penial sculpture. Spermatophore: tail filament near sperm sac with three spines and terminal part more than ca. one-third of its length with series of branching spines.

###### Description.

***Shell*.** Shell depressed to strongly depressed, large size (shell width up to 24.7 mm, shell height up to 12.0 mm), and thin. Surface smooth and shiny; shell colour very pale brown to dark brown. Whorls 6–6½, increasing regularly; body whorl large and rounded to weak shouldered. Spire moderately elevated; suture impressed. Aperture crescent-shaped and obliquely opened. Peristome simple. Columellar margin simple and slightly reflected near umbilicus. Umbilicus narrowly opened (Fig. 38E, F).

***Genital organs*.** Atrium short. Penis cylindrical with thin penial sheath covering proximal penis. Inner sculpture of penis proximally more than ca. half of penial chamber with very finely longitudinal penial pilasters to nearly smooth surface, and then gradually transformed from small to large rhomboid pilasters with acute angle on top (triangular prism). Epiphallus enlarged cylindrical and approximately two times penis length. Epiphallic caecum large, straight, similar to epiphallus diameter and located near middle of epiphallus. Penial retractor muscle thin and attached at tip of epiphallic caecum. Flagellum long and enlarged approximately as long as epiphallus. Vas deferens thin tube connecting distal epiphallus and free oviduct (Fig. 41).

Vagina long cylindrical and approximately twice as long as penis. Dart apparatus large, long cylindrical, and located on atrium at vagina and penis junction. Gametolytic sac enlarged and bulbous; gametolytic duct enlarged cylindrical (spermatophore inside). Free oviduct cylindrical, slightly shorter than vagina (Fig. 41A).

Spermatophore long and needle-shaped. Sperm sac enlarged and elongate-oval. Head filament was missing (incomplete spermatophore). Tail filament very long tube; region near sperm sac with three spines. Spine I simple and rather short. Spine II large and long, and most of branching spines probably missing. Spine III smaller than spine II, branching into small spines and spinules. Region furthest away smooth and without spine; terminal part (more than ca. one-third of its length) with series of long branching spines arranged in a row or encircled the tail filament tip (Fig. 42).

***Radula*.** Teeth with half row formula: 1–(15–16)–50. Central tooth symmetrical tricuspid; lateral teeth asymmetrical tricuspid; marginal teeth elongate bicuspid. Marginal teeth starting at approximately row number 15 or 16 (Fig. 43E).

***External features*.** Animal with reticulated skin and blackish body. Foot sole and caudal foss present; caudal horn raised. Four mantle lobes well developed and same colour as body. Left shell lobe absent (Fig. 33F).

###### Etymology.

The specific name *pellosa* is from the Greek word *pellos* meaning dusky and refers to the blackish body that characterises this species.

###### Distribution.

This species is only known from several limestone karsts in Sa Kaeo Province (Fig. 32).

###### COI analysis.

The ML and BI analyses of *S. pellosa* sp. nov. (n = 3) revealed that all specimens formed a well-supported clade (Fig. 1; BS = 96%, PP = 1), sister group to *S. inferospira* sp. nov. + *S. melanospira* sp. nov. + *S. bocourti* with moderate support (Fig. 1; BS = 73%, PP = 0.99). The mean intraspecific genetic distance of *S. pellosa* sp. nov. was 2.1% (Table 2).

###### Remarks.

The shell of *S. pellosa* sp. nov. differs from other species in *Sarika hainesi* group by having a rounded to very weak shouldered body whorl. In contrast, the shells of *S. hainesi* and *S. bocourti* have obtusely angulated body whorls and *S. inferospira* sp. nov. has a shouldered body whorl.

The shell of this new species is generally similar to *S. melanospira* sp. nov. The distinguishing characters of *S. pellosa* sp. nov. are a broader body whorl, larger size of flagellum, vagina and free oviduct, and animal without a dark spiral band, while *S. melanospira* sp. nov. has a broad body whorl, smaller size of flagellum, vagina and free oviduct, and animal with a dark spiral band below the suture at the body whorl. In addition, the average interspecific sequence divergences between *S. pellosa* sp. nov. and *S. melanospira* sp. nov. are fairly high (6.6%). Therefore, we treat them as two separate species.

#### Group III: *Sarika dugasti* group: species with left shell lobe and penial pseudo-verge.

##### Sarika dugasti

Taxon classificationAnimaliaStylommatophoraAriophantidae

(Morlet, 1891)

EDA6D4EC-96C4-5350-896C-93E448FB0A16

**Figure 44.**
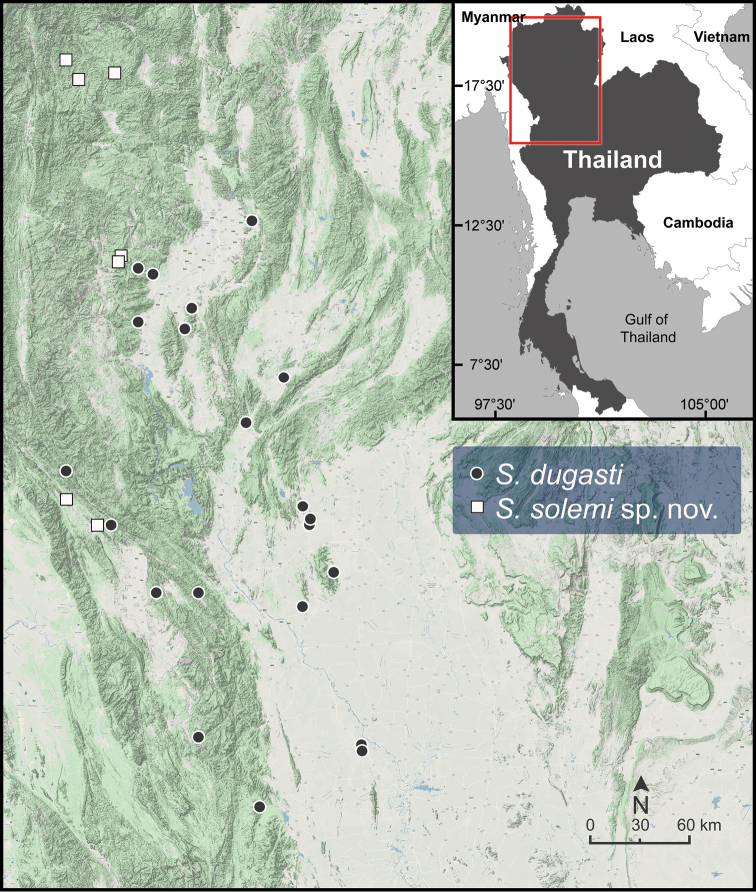
Geographic distribution of *Sarika dugasti* and *S. solemi* sp. nov. based on the specimens examined herein.

**Figure 45.**
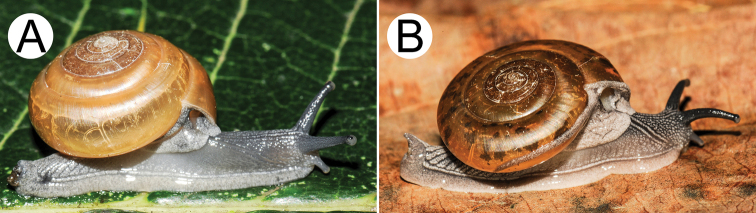
Living snails of Group III: *Sarika dugasti* group. **A***Sarika dugasti* specimen CUMZ 7563 and **B***S. solemi* sp. nov. paratype CUMZ 7298. Not to scale.

**Figure 46.**
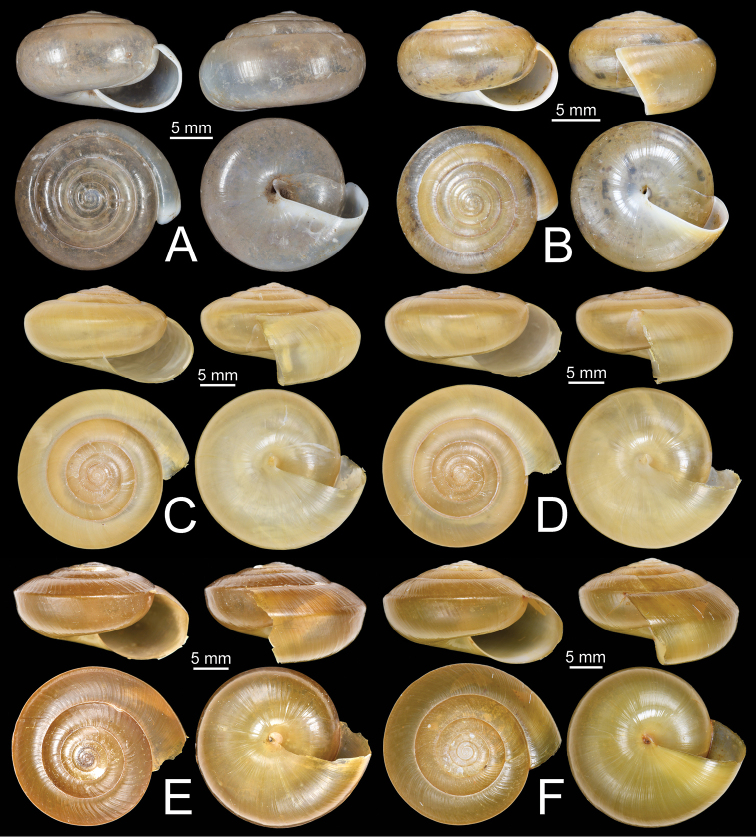
Shells of Group III: *Sarika dugasti* group. **A, B***Sarika dugasti***A** syntype MNHN-IM-2000-27884 and **B** specimen CUMZ 7542, **C–F***S. solemi* sp. nov. **C** holotype CUMZ 7297 **D** paratype CUMZ 7298, and **E, F** specimen CUMZ 7503.

**Figure 47.**
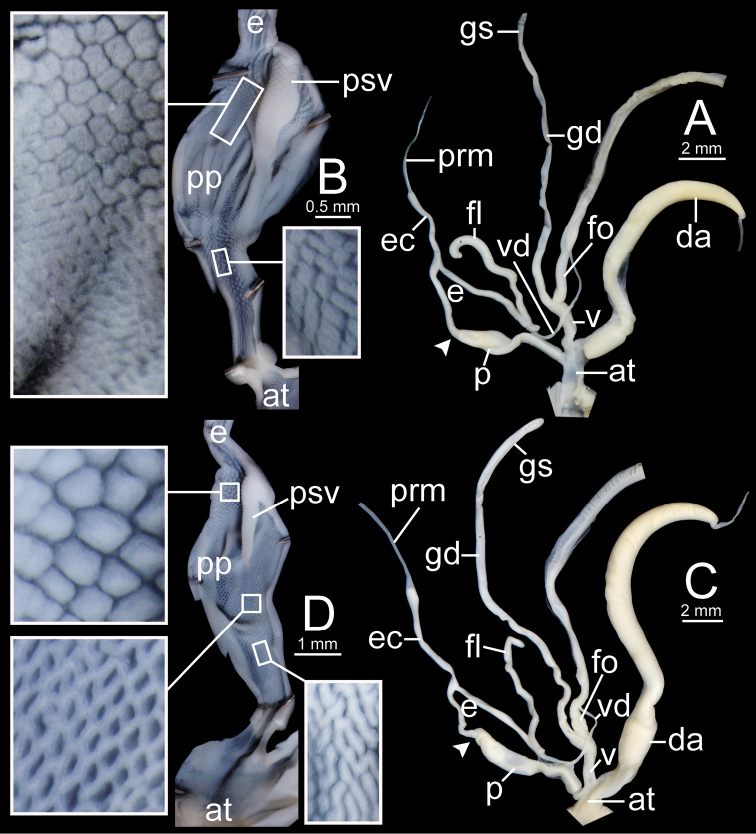
Genitalia. **A, B***Sarika dugasti* specimen CUMZ 7542 **A** general view of the genital system and **B** internal structure of the penis **C, D***S. solemi* sp. nov. paratype CUMZ 7298 **C** general view of the genital system and **D** internal structure of the penis. White arrowheads indicate the ends of the penes.

**Figure 48.**
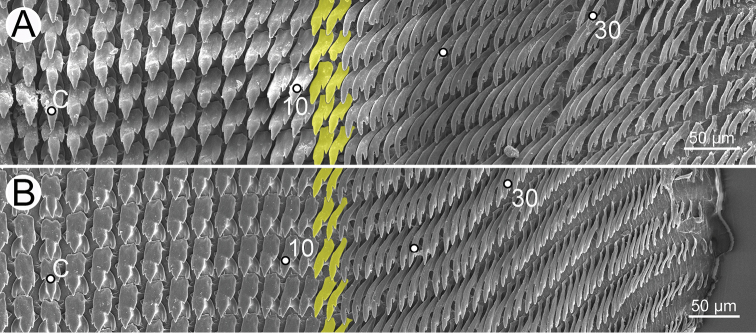
Representative SEM images of the radula. **A***Sarika dugasti* specimen CUMZ 7574 and **B***S. solemi* sp. nov. paratype CUMZ 7298. Central tooth indicated by ‘C’; Yellow colour indicates lateral teeth with the transition to marginal teeth.

Figs 1 , 44 , 45A , 46A, B , 47A, B , 48A

Macrochlamys dugasti Morlet, 1891a: 25, 26. Type locality: “forêts des bords du Ménam-Pinh, Laos occidental” [forest edges of Ping River, Thailand]; Fischer-Piette 1950: 159; Panha 1996: 34; Hemmen and Hemmen 2001: 44.
Ariophanta (Macrochlamys) dugasti : Morlet 1891b: 231, 239, 240, pl. 5, figs 1, 1a; Fischer 1891: 20.
Nanina (Macrochlamys) dugasti : Fischer and Dautzenberg 1904: 395.
Sarika dugasti : Tomlin 1929: 16. Maneevong 2000: 31, fig. 4–3; Sutcharit and Panha 2008: 96; Schileyko 2011: 34. Inkhavilay et al. 2019: 149, fig. 59f; Pholyotha et al. 2020c: 19, fig. 9f.

###### Type material.

***Syntype***MNHN-IM-2000-27884 (one shell; Fig. 46A) from Forêts des bords du Ménam-Pinh, Laos occidental [forest edges of Ping River, Thailand]. Possible syntype NHMUK 1893.12.8.31 (one shell).

###### Other material examined.

**Myanmar.** Phaboo, Salwin Valley, Burma: NHMUK 1891.3.17.559 60 (two shells). Moulmein: NHMUK 1887.06.1.1 (two shells). **Thailand.** Siam: NHMUK 1903.7.1.100, NHMUK 1901.1.06.38 (specimen figured in Blanford and Godwin-Austen 1908: Fig. 48), SMF 90841/2 (two shells). **Thailand-Western.** Wat Tham Inthanin, Mae Sot, Tak, 16°46'01.4"N, 98°40'21.5"E: CUMZ 7567. Limestone outcrop in Mae Sot, Mae Sot, Tak, 16°45'40.8"N, 98°43'08.1"E: CUMZ 7568, 7569. Chao Por Phawo Shrine, Mae Sot, Tak, 16°46'16.9"N, 98°41'09.1"E: CUMZ 7571, 7572, 7573, 7574. Limestone outcrop in Tha Song Yang, Tha Song Yang, Tak, 17°27'11.8"N, 98°10'39.2"E: CUMZ 7570. Ban Nam Ok Hu, Tha Song Yang, Tak, 17°07'54.4"N, 98°23'41.5"E: CUMZ 7575. Limestone outcrop in Umphang, Umphang Tak, 16°00'53.4"N, 98°56'47.7"E: CUMZ 7576. **Thailand-Central.** Pitsunaloke, Siam: NHMUK 1903.2.4.82–84 (four shells). Limestone area near Huai Kha Khaeng, Lan Sak, Uthai Thani, 15°36'31.1"N, 99°19'15.0"E: CUMZ 7543. Limestone outcrop in Phran Kratai, Phran Kratai, Kamphaeng Phet, 16°40'40.1"N, 99°32'27.1"E: CUMZ 7541. Wat Khao Huai Lung, Banphot Phisai, Nakhon Sawan, 15°55'30.2"N, 99°52'28.9"E: CUMZ 7564. Wat Thep Sathaporn, Banphot Phisai, Nakhon Sawan, 15°54'48.7"N, 99°53'04.0"E: CUMZ 7565, 7566. Tham Yok, Ban Dan Lan Hoi, Sukhothai, 17°08'15.9"N, 99°33'00.5"E: CUMZ 7542 (Fig. 46B). Limestone outcrop in Na Choeng Khiri, Khiri Mat, Sukhothai, 16°52'38.7"N, 99°41'35.1"E: CUMZ 7557. Tham Lom-Tham Wang, Si Samrong, Sukhothai, 17°13'30.3"N, 99°31'53.0"E: CUMZ 7558, 7559, 7560. Wat Tham Rakhang, Si Samrong, Sukhothai, 17°09'54.2"N, 99°33'36.2"E: CUMZ 7561, 7562, 7563. **Thailand-Northern.** Lampun, Siam: SMF 298588/2 (two shells). Lampoon, Siam: NHMUK MacAndrew Coll. Acc. No. 1563 (three shells), NHMUK Kennard Coll. Acc. No. 1824 (one shell), NHMUK ex. 392 (five shells). Tham Luang Pha Wiang, Ban Hong, Lamphun, 18°13'18.7"N, 98°51'27.7"E: CUMZ 7551, 7552, 7553, 7554, 7577. Erawan Cave, Pa Sang, Lamphun, 18°19'38.4"N, 98°52'17.9"E: CUMZ 7555, 7556. Wat Tham Pha Ngam, Mae Phrik, Lampang, 17°28'49.6"N, 99°10'05.3"E: CUMZ 7549. Wat Tham Suk Kasem Sawan, Thoen, Lampang, 17°42'40.0"N, 99°12'25.2"E: CUMZ 7550. Wachirathan Waterfall, Chom Thong, Chiang Mai, 18°32'31.2"N, 98°35'53.7"E: CUMZ 7544. Borichinda Cave, Chom Thong, Chiang Mai, 18°30'02.8"N, 98°40'20.4"E: CUMZ 7545, 7546. Wat Tham Tong, Chom Thong, Chiang Mai, 18°15'19.8"N, 98°34'48.0"E: CUMZ 7547. Wat Tham Muang On, Mae On, Chiang Mai, 18°47'46.3"N, 99°14'05.5"E: CUMZ 7548.

###### Diagnosis.

Shell medium, globosely depressed, pale to dark brown with well-rounded body whorl. Animal with greyish body and five mantle lobes. Genitalia with a long straight epiphallic caecum and long pseudo-verge. Inner penial sculpture with small cuboidal pilasters in proximal part, then reticulated pilasters in the middle, and larger cuboidal pilasters in distal end.

###### Description.

***Shell*.** Shell globosely depressed, medium size (shell width up to 17.2 mm, shell height up to 10.5 mm), and rather thin. Shell surface smooth and shining; shell colour very pale to dark brown. Whorls 6½–7½, increasing regularly; body whorl large and well rounded. Spire much elevated; suture impressed. Aperture crescent-shaped and obliquely opened. Peristome simple and slightly thickened. Columellar margin simple and slightly reflected near umbilicus. Umbilicus narrowly opened (Fig. 46A, B).

***Genital organs*.** Atrium short. Penis long cylindrical with thin penial sheath covering penis. Proximal penis rather slender; distal penis enlarged with pseudo-verge inside. Inner sculpture of penis proximally with very finely longitudinal penial pilasters to nearly smooth surface, then transformed to small cuboidal and reticulated pilaster in middle and modified to larger cuboidal pilasters at distal end. Pseudo-verge elongate conic and approximately one-third of penis length. Epiphallus cylindrical, and narrower than distal penis. Epiphallic caecum very long, straight, and same diameter as epiphallus. Penial retractor muscle thin and attached at tip of epiphallic caecum. Flagellum long, slender, and approximately as long as penis. Vas deferens thin tube connecting distal epiphallus and free oviduct (Fig. 47A, B).

Vagina short and approximately one-third of penis length. Dart apparatus large, long cylindrical, and located on atrium at vagina and penis junction. Gametolytic organ (sac and duct) small and long cylindrical tube. Free oviduct cylindrical, approximately as long as vagina, and proximal end encircled with thick tissue (Fig. 47A).

***Radula*.** Teeth with half row formula: 1–(11–12)–47. Central tooth symmetrical tricuspid; lateral teeth asymmetrical tricuspid; marginal teeth elongate bicuspid. Marginal teeth starting at approximately row number 11 or 12 (Fig. 48A).

***External features*.** Animal with reticulated skin, greyish body, slightly pale colour on foot sole and darker colour on caudal horn. Mantle edge well developed with three dorsal lobes and two shell lobes, and similar colour to body (Fig. 45A).

###### Distribution.

*Sarika dugasti* occurs in central, north and western Thailand along the Tenasserim Ranges (Fig. 44) and the Salween Valley, east of the Dawna Ranges in Myanmar (Blanford and Godwin-Austen 1908; Pholyotha et al. 2020c). However, the literature records from Laos, Nepal and Vietnam (Schileyko 2011; Inkahvilay et al. 2019) are still uncertain.

###### COI analysis.

The ML and BI analyses revealed that the individuals of *S. dugasti* (n = 3) formed a monophyletic group with good support (Fig. 1; BS = 91%, PP = 1). The mean intraspecific genetic distance of *S. dugasti* was 3.7% (Table 2).

###### Remarks.

*Sarika dugasti* can be distinguished from all other known *Sarika* species by having a dome-shaped shell with narrow aperture and genitalia with a very long epiphallic caecum and long pseudo-verge. Other *Sarika* species tend to have a flattened to depressed shell with a wide aperture and genitalia without penial verge. Although we surveyed during the wet season, only immature snails were collected, and so the radula and genitalia of sub-adult specimens are illustrated here.

##### Sarika solemi

Taxon classificationAnimaliaStylommatophoraAriophantidae

Pholyotha & Panha sp. nov.

BB61E2A5-AE39-5389-8942-9DDBA77B3151

http://zoobank.org/FC15ADFA-11D7-413F-8AED-21FE6C7C2B27

Figs 1 , 44 , 45B , 46C–F , 47C, D , 48B

Sarika aff. hainesii [sic]: Solem 1966: 38, 39, fig 5a (non Pfeiffer 1856a: 32); Sutcharit and Panha 2008: 96.

###### Type material.

***Holotype***CUMZ 7297 (Fig. 46C, width 24.4 mm, height 13.5 mm). ***Paratypes***CUMZ 7298 (15 shells and 26 specimens preserved in ethanol; Fig. 46D, width 24.8 mm, height 12.7 mm), NHMUK 20200288 (two shells), SMF (two shells), ZRC.MOL.017030 (two shells).

###### Other material examined.

**Thailand-Western.** Ban Nam Ok Hu, Tha Song Yang, Tak, 17°08'01.2"N, 98°22'01.8"E: CUMZ 7504. Mae Usu Cave, Tha Song Yang, Tak, 17°18'14.7"N, 98°09'20.8"E: CUMZ 7505. **Thailand-Northern.** Mountain area in Mae Na Toeng, Pai, Mae Hong Son, 19°34'40.5"N, 98°25'55.9"E: CUMZ 7300. Wat Pang Kham, Pang Mapha, Mae Hong Son, 19°41'03.3"N, 98°12'30.5"E: CUMZ 7501. Huai Nam Dang, Mae Taeng, Chiang Mai, 19°18'48.9"N, 98°36'19.2"E: CUMZ 7299. Kew Mae Pan, Chom Thong, Chiang Mai, 18°33'21.1"N, 98°28'56.0"E: CUMZ 7503 (Fig. 46E, F). Doi Inthanon, Chom Thong, Chiang Mai, 18°35'16.9"N, 98°29'13.4"E: CUMZ 7502. Mountain area in Chom Thong, Chiang Mai, 18°32'05.2"N, 98°31'02.8"E: CUMZ 7911.

###### Type locality.

The limestone karsts with dry forest near Mae La Na Cave, Pang Mapha, Mae Hong Son, Thailand, 19°34’25.5"N, 98°13’01.8"E.

###### Diagnosis.

Shell large, depressed and yellowish brown to brown with obtusely angulated to angulated body whorl. Animal with creamy-grey body and five mantle lobes. Genitalia with a long straight epiphallic caecum and long pseudo-verge. Inner penial sculpture with irregularly short folded pilasters in proximal part, then reticulated pilasters in the middle, and cuboidal pilasters in distal end.

###### Description.

***Shell*.** Shell depressed, large size (shell width up to 26.5 mm, shell height up to 15.0 mm) and rather thin. Surface rather smooth and polished; shell colour yellowish brown to brown. Whorls 6–6½, increasing regularly; body whorl large and obtusely angulated to angulated. Spire moderately to very much elevated; suture impressed. Aperture crescent-shaped and obliquely opened. Peristome simple. Columellar margin simple and slightly reflected near umbilicus. Umbilicus narrowly opened (Fig. 46C–F).

***Genital organs*.** Atrium short. Penis cylindrical with thin penial sheath covering proximal penis. Proximal penis rather slender; distal penis enlarged with pseudo-verge inside. Inner sculpture of penis proximally with very finely longitudinal penial pilasters to nearly smooth surface, then changed to irregularly short folded pilasters, modified to reticulated pilasters in middle, and modified to cuboidal pilasters at distal end. Pseudo-verge long conic, approximately one-third of total penis length. Epiphallus long cylindrical and narrower than distal penis. Epiphallic caecum very long, straight, and almost same diameter as epiphallus. Penial retractor muscle thin and attached at tip of epiphallic caecum. Flagellum long slender, and approximately as long as penis. Vas deferens thin tube connecting distal epiphallus, and free oviduct (Fig. 47C, D).

Vagina cylindrical and approximately one-fourth of penis length. Dart apparatus large, long cylindrical, and located on atrium at vagina and penis junction. Gametolytic organ (sac and duct) small and long duct. Free oviduct cylindrical and proximal end encircled with thick tissue (Fig. 47C).

***Radula*.** Teeth with half row formula: 1–(12–13)–54. Central tooth symmetrical tricuspid; lateral teeth asymmetrical tricuspid; marginal teeth elongate bicuspid. Marginal teeth starting at approximately row number 12 or 13 (Fig. 48B).

***External features*.** Animal with reticulated skin and dark creamy mixing with grey to dark grey body, very pale grey foot sole and pale grey caudal horn. Five mantle lobes well developed and same colour as body (Fig. 45B).

###### Etymology.

The specific name *solemi* is named in honor of Dr. Alan Solem, who first discovered and described the genitalia of this species but under the name Sarika aff. hainesii.

###### Distribution.

*Sarika solemi* sp. nov. seems to be restricted to western and northern Thailand along the Tenasserim Ranges (Fig. 44). This species occurs in forested mountains and is highly abundant in limestone habitats.

###### COI analysis.

The ML and BI analyses showed that the individuals of *S. solemi* sp. nov. (n = 3) formed a monophyletic group with high support (Fig. 1; BS = 95%, PP = 1). The mean intraspecific genetic distance of *S. solemi* sp. nov. was 2.9% (Table 2).

###### Remarks.

Solem (1966) examined and described the genitalia of specimens from northern Thailand and referred them to as “Sarika aff. hainesii”. In this study, we collected and examined several new specimens from northern Thailand and found that the genitalia were identical with those described and illustrated in Solem (1966: 38, 39, fig 5a). We here recognised these populations as new species. *Sarika solemi* sp. nov. has similar shell morphology to *S. hainesi* s.s., but the distinguishing characters are the number of mantle lobes and genitalia. This new species has five mantle lobes and genitalia with very long epiphallic caecum and long pseudo-verge, while *S. hainesi* s. s. has four mantle lobes, and genitalia with shorter epiphallic caecum and without pseudo-verge.

*Sarika solemi* sp. nov. is a variable species in terms of body whorl with obtusely angulated periphery (Fig. 46C, D) to obvious angulated periphery (Fig. 46E, F). All shell morphs are identical in genital characters and form a well-supported clade in COI analysis.

#### Species of doubtful status or uncertain record for Thailand

The following six species have never been examined for their genitalia and no living specimens could be collected in this study. However, we assign them to the genus *Sarika* following current literature and based on their shell characters. They have a relatively large shell diameter (greater than 20 mm), and a smooth and polished shell surface. *Macrochlamys* from Thailand (Pholyotha et al. 2018) tend to have a much smaller shell diameter (less than 20 mm) than the genus *Sarika* (see Table 5). However, living specimens are still necessary for examination of the reproductive organs to ascertain their generic placement.

##### Sarika gratesi

Taxon classificationAnimaliaStylommatophoraAriophantidae

Pholyotha & Panha sp. nov.

B6073C89-28CE-513D-B8B0-8369C717ED51

http://zoobank.org/B61773AB-0762-4B4D-A097-162ED9C43720

Figs 49 , 50A, B

###### Type material.

***Holotype***CUMZ 7275 (Fig. 50A, width 16.5 mm, height 8.8 mm). ***Paratypes***CUMZ 7276 (10 shells; Fig. 50B, width 16.0 mm, height 8.3 mm), 7912 (30 shells), NHMUK 20200289 (two shells), SMF (two shells), ZRC.MOL.017031 (two shells).

###### Other material examined.

**Thailand-Northeastern.** Dry dipterocarp forest at Phu Lan Kha, Nong Bua Daeng, Chaiyaphum, 16°00'00.9"N, 101°52'33.4"E: CUMZ 7277.

###### Type locality.

The limestone outcrop with dry deciduous forest at Tham Phraya Nakarat (Cave), Chum Phae, Khon Kaen, Thailand, 16°48'30.3"N, 101°57'13.7"E.

###### Diagnosis.

Shell medium-sized, depressed to strongly depressed, and pale brown. Aperture irregular with peristome rather simple above then expanded middle with curved inside aperture and thickened below periphery.

**Figure 49.**
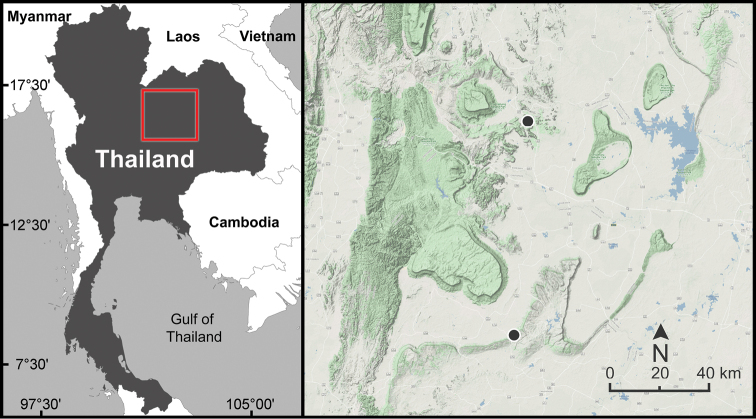
Geographic distribution of *Sarika gratesi* sp. nov. based on the specimens examined herein.

###### Description.

**Figure 50.**
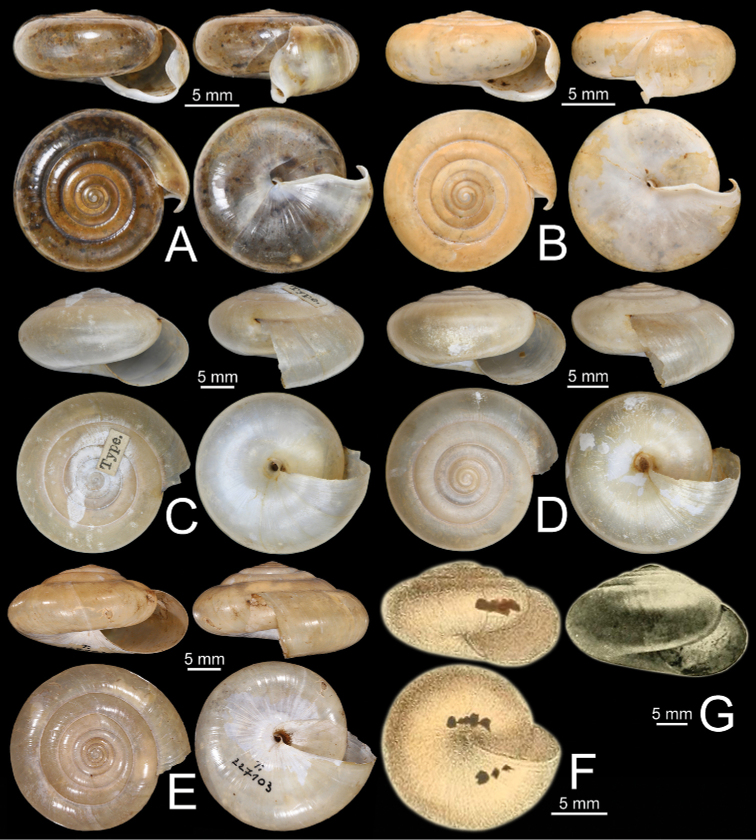
Shells of *Sarika* spp. **A, B***Sarika gratesi* sp. nov. **A** holotype CUMZ 7275 and **B** paratype CUMZ 7276 **C, D***S. pumicata* syntype NHMUK 1893.2.4.980–981 **E***S. ochtogyra* holotype SMF 227103/1 **F***S. benoiti* modified from Crosse and Fischer (1863)**G***S. rex* modified from Preston (1909).

***Shell*.** Shell depressed to strongly depressed or nearly flattened, medium-sized (shell width up to 17.4 mm, shell height up to 8.8 mm), rather thin, and slightly opaque. Shell surface smooth, polished and with thin growth lines; shell colour pale brown. Whorls 6–7, increasing regularly; body whorl large and well rounded. Spire little to moderately elevated; suture impressed. Aperture crescent-shaped and open obliquely; peristome irregular. Apertural lip at upper periphery simple; at periphery with invagination of triangular lip (beak-like); at below periphery rather thickened inside aperture and little expanded. Columellar margin straight, slightly thickened and expanded near umbilicus. Umbilicus narrowly opened (Fig. 50A, B).

###### Etymology.

The specific name *gratesi* is named in honour to Admiral Chorchat Gra-tes of the Royal Thai Navy, who made possible many fieldtrips especially the remote islands areas in Thailand.

###### Distribution.

*Sarika gratesi* sp. nov. is currently known from the restricted area of the dry deciduous and dry dipterocarp forests in Khon Kaen and Chaiyaphum provinces (Fig. 49).

###### Remarks.

This new species is easy to distinguish from all known *Sarika* as well as *Macrochlamys* species by its unique beak-like lip, while all other species in these two genera have simple to little thickened lips. Only, *Macrochlamys aspides* (Benson 1863) from Myanmar tends to have a slightly thickened and expanded lip at below periphery, but *Sarika gratesi* sp. nov. has a projecting and curved triangular shape lip at the periphery. *Sarika gratesi* sp. nov. is identical to *Sarika* because its shells are matched with *Sarika* more than the Siam *Macrochlamys* (Pholyotha et al. 2018).

##### Sarika subcornea

Taxon classificationAnimaliaStylommatophoraAriophantidae

(Pfeiffer, 1861)

C9B5B1BB-DB3F-5CD1-8FBF-0DE40EAAF7E5

Helix subcornea Pfeiffer, 1861: 20. Type locality: “Siam” [Thailand]; Pfeiffer 1868: 102, 103.
Nanina (Orobia) subcornea : Martens 1867: 73.
Nanina (Macrochlamys) subcornea : Tryon 1886: 92; Fischer and Dautzenberg 1904: 395.
Ariophanta (Macrochlamys) subcornea : Fischer 1891: 21.
Nanina subcornea : Fischer and Dautzenberg 1904: 395; Hemmen and Hemmen 2001: 45.
Sarika subcornea : Maassen 2001: 114.

###### Type material.

The type specimen could not be located in the NHM collections.

###### Diagnosis.

Shell depressed, medium size (shell width up to 13.6 mm, shell height up to 6.0 mm) and thin. Shell surface smooth and shining; shell colour whitish horny. Whorls 7½, increasing regularly; body whorl well rounded. Spire elevated; suture impressed. Aperture crescent-shaped and obliquely opened. Peristome simple and slightly thickened. Columellar margin simple. Umbilicus narrowly opened.

###### Remarks.

The original description is not illustrated, type specimen could not be traced, and no new specimens have been reported so far. Hanley and Theobald (1876: 59, pl. 149, Figs 2, 3) illustrated this species based on a specimen from “Phie Than” [Payathonzu, Myanmar]. But, Blanford and Godwin-Austen (1908) stated that this specimen was instead a juvenile shell of *S. resplendens*. Recently, Maassen (2001) also reported *S. subcornea* as a new record from Kedah, Malaysia, but unfortunately without illustration. Therefore, the taxonomic status of this species is still unclear, and we retained this species within the genus *Sarika* as per Maassen (2001). Godwin-Austen (1883) noted that *S. subcornea* is distinguished from other species by the relatively more numerous and more closely wound whorls.

##### Sarika benoiti

Taxon classificationAnimaliaStylommatophoraAriophantidae

(Crosse & Fischer, 1863)

72378200-17DE-57D9-871C-3696B80C3652

Fig. 50F

Zonites benoiti Crosse & Fischer, 1863: 346, pl. 14, fig. 4. Type locality: “in loco Fuyen-Moth dicto, Cochinchine” [Phu Yen Province, Vietnam].
Helix benoiti : Pfeiffer 1868: 99; Morelet 1875: 248.
Nanina (Macrochlamys) benoiti : Tryon 1886: 90, pl. 30, fig. 57, 58.
Ariophanta (Macrochlamys ?) benoiti: Fischer 1891: 20.
Macrochlamys benoiti : Ancey 1898: 128, pl. 9, Fig. b; Saurin 1953: 113; Schileyko 2011: 30, 31.
Sarika benoiti : Inkhavilay et al. 2019: 81, fig. 38c; Pholyotha et al. 2020a: 7.

###### Type material.

No type material could be located in the MNHN collections. The illustration from the original description is reproduced here (Fig. 50F).

###### Diagnosis.

Shell depressed, medium size (shell width up to 16.0 mm, shell height up to 9.0 mm), and thin. Shell surface smooth and shining; shell colour brownish. Whorls 6, increasing regularly; body whorl well rounded. Spire elevated; suture impressed. Aperture crescent-shaped and obliquely opened. Peristome simple. Columellar margin simple. Umbilicus narrowly opened (Fig. 50F).

###### Remarks.

This species was described based on a specimen from south-central Vietnam (Crosse and Fischer 1863). Up to now, *Sarika benoiti* has been recorded throughout the Indochina countries (Ancey 1898, Fischer and Dautzenberg 1904, Saurin 1953, Schileyko 2011, Inkhavilay et al. 2019). However, these records remain equivocal and tentative because of unavailable distinguished genital data. In Thailand, *Sarika benoiti* was recorded from eastern Thailand based on specimens from the Pavie collection (Fischer and Dautzenberg 1904).

In this study, the specimens from the eastern part of Thailand agreed well with the syntype of *S. bocourti* (Fig. 34A) where its type locality is in Battambang, Cambodia. Based on shell morphology, *S. benoiti* (Fig. 50F) differs slightly from *S. bocourti* in having a smaller sized and well-rounded body whorl, whereas *S. bocourti* has a larger sized shell (shell width up to 33 mm) and obtusely angulated body whorl.

##### Sarika pumicata

Taxon classificationAnimaliaStylommatophoraAriophantidae

(Morelet, 1875)

5DCC6A53-5CAE-557E-BE80-6CFF309AF82F

Fig. 50C, D

Helix pumicata Morelet, 1875: 248, pl. 12, fig. 2. Type locality: “Ajuthia, Siam” [Phra Nakhon Si Ayutthaya Province, Thailand]; Breure et al. 2018: 399, figs 901, 902.
Nanina (Macrochlamys) pumicata : Tryon 1886: 89, pl. 29, figs 40–42; Fischer and Dautzenberg 1904: 395.
Ariophanta (Xesta) pumicata : Fischer 1891: 20.
Sarika pumicata : Godwin-Austen 1907: 181, pl. 116, fig. 1; Sutcharit and Panha 2008: 96.
Macrochlamys pumicata : Panha 1996: 34; Hemmen and Hemmen 2001: 44.

###### Type material.

***Syntype***NHMUK 1893.2.4.980–981 (two shells; Fig. 50C, D) from Siam [Thailand].

###### Other material examined.

Siam: NHMUK 1901.1.6.246 (one broken shell, specimen dissected by Godwin-Austen 1907) ex. Daly collection.

###### Diagnosis.

Shell depressed to conoid-depressed, large size (shell width up to 26.0 mm, shell height up to 16.0 mm) and rather thin. Shell surface smooth, slightly shining above and more shining below; shell colour brownish. Whorls 7, increasing regularly; body whorl large and obtusely angulated. Spire high-conical; suture impressed. Aperture crescent-shaped and obliquely opened. Peristome simple. Columellar margin simple. Umbilicus narrowly opened (Fig. 50C, D).

###### Remarks.

Godwin-Austen (1907: 181, pl. 116, Fig. 1) examined the rehydrated specimens from Thailand and placed this species in the genus *Sarika*. We have examined the specimen dissected (only shell remained) by Godwin-Austen (NHMUK 1901.1.6.246) and noticed that the shell morphology slightly differs from the syntypes. The dissected specimen has a rounded body whorl and lower spire, whereas the syntypes have obtusely angulated body whorl and higher spire. Unfortunately, we could not find any specimens identical to the syntypes during this survey. Additional specimens with precise collection locality and anatomical studies are necessary to confirm the taxonomic position of this species.

##### Sarika ochtogyra

Taxon classificationAnimaliaStylommatophoraAriophantidae

(Möllendorff, 1902)

923EB2EE-083A-5E74-B900-F4899BB4DA9C

Fig. 50E

Macrochlamys ochtogyra Möllendorff 1902: 154, 155. Type locality: “Bangkok”; Hemmen and Hemmen 2001: 44.

###### Type material.

***Syntype***SMF 227103/1 (one shell; Fig. 50E) from Siam: Bangkok.

###### Diagnosis.

Shell depressed, large to very large size (shell width up to 31.8 mm, shell height up to 16.0 mm) and thin. Shell surface rather smooth surface with obvious growth lines; shell colour yellowish brown. Whorls 8, increasing regularly; body whorl large and obtusely angulated. Spire high-conical; suture impressed. Aperture crescent-shaped and obliquely opened. Peristome simple. Columellar margin simple. Umbilicus narrowly opened (Fig. 50E).

###### Remarks.

Compared to the species with shell width greater than 30 mm, *S. ochtogyra* can be distinguished from *S. rex* by having eight whorls and obtusely angulated periphery, while *S. rex* has seven whorls and rounded periphery.

*Sarika ochtogyra* is currently known only from the type locality in Thailand. Originally, it was described based on a collection made by the butterfly collector, H. Frühstorfer (Lamas 2005). The type locality “Siam: Bangkok” is probably not the location where the type specimen was collected. After surveys throughout Thailand, we could find neither *Sarika* nor *Macrochlamys* with large shell sizes that match well with the type specimen.

##### Sarika rex

Taxon classificationAnimaliaStylommatophoraAriophantidae

(Preston, 1909)

1E26B15F-44E0-512E-9A02-F10A040F2DF6

Fig. 50G

Macrochlamys rex Preston, 1909: 202, pl. 8, fig. 2. Type locality: “Nan-ko, Siam” [Thailand]; Adam, 1971: 58.

###### Type material.

The unique name bearing type could not be located. The photograph of the type specimen from the original description is reproduced herein (Fig. 50G).

###### Diagnosis.

Shell conoid-depressed, large to very large size (shell width up to 30.0 mm, shell height up to 16.0 mm) and thin. Shell surface rather smooth with fine growth lines, polished below but not polished above; shell colour pale yellowish brown. Whorls 6½, increasing regularly; body whorl large and rounded. Spire very high-conical; suture impressed. Aperture crescent-shaped and obliquely opened. Peristome simple. Columellar margin simple. Umbilicus narrowly opened (Fig. 50G).

###### Remarks.

We assigned this species to the genus *Sarika* due to its very large shell size, and none of any *Macrochlamys* species could reach that diameter (Table 5). Preston (1909) noted that *S. rex* is a large-sized species and is generally similar to *S. pumicata*, but *S. rex* tended to have more tumid and higher spire than *S. pumicata*, as well as a rounded body whorl, whereas *S. pumicata* has an obtusely angulated periphery.

*Sarika rex* is known only from the type locality in Thailand; however, the precise type locality could not be determined. To date, no living specimen that matches this species has been found, and the generic assignment is still provisional. New specimens with precise collection locality and genital anatomy are necessary to verify the taxonomic position of this species.

## Conclusions

This study updates the state of knowledge of malacofaunal diversity in Thailand and the results increase the number of *Sarika* species recognised in the country to 23, nine of which are new species. Our analyses of morphology and molecular phylogeny resolve and support all *Sarika* species anatomically examined herein (see Fig. 1; Tables 2–5).

**Table 3.** Shell measurements of *Sarika* species in Thailand. The superscript indicates spermatophore morphology reference: ^1^ this study, ^2^Pfeiffer (1861), ^3^Crosse and Fischer (1863), ^4^Morelet (1875), ^5^Möllendorff (1902), and ^6^Preston (1909).

*Sarika* species (no. specimens)	Shell width (mm), mean ± SD	Shell height (mm), mean ± SD	Number of whorls
1 *S. resplendens*^1^ (n = 10)	21.0–23.4,	9.9–11.5,	5½–6½
	22.1 ± 1.0	10.6 ± 0.5	
2 *S. dohrniana*^1^ (n = 10)	27.1–33.2,	15.2–18.9,	6–6½
	30.0 ± 2.1	17.1 ± 1.2	
3 *S. obesior*^1^ (n = 10)	19.7–22.1,	10.1–11.3,	5½–6½
	20.8 ± 0.8	10.7 ± 0.4	
4 *S. limbata*^1^ (n = 11)	23.6–27.0,	11.1–13.7,	6–6½
	25.0 ± 1.1	12.2 ± 0.8	
5 *S. heptagyra*^1^ (n = 3)	21.9–27.9,	10.6–13.1,	6–7
	25.8 ± 3.4	12.0 ± 1.3	
6 *S. kawtaoensis*^1^ (n = 11)	22.4–26.6,	12.1–15.2,	6–7
	24 ± 1.5	13.3 ± 0.9	
7 *S. caligina* sp. nov.^1^ (n = 10)	22.7–25.7,	10.2–12.3,	6–6½
	24.6 ± 0.8	11.6 ± 0.6	
8 *S. lactospira* sp. nov.^1^ (n = 10)	21.1–23.6,	10.1–11.8,	6–6½
	22.5 ± 0.9	10. 9 ± 0.6	
9 *S. megalogyne* sp. nov.^1^ (n = 10)	17.8–21.2,	9.3–11.3,	6–6½
	19.2 ± 1.0	10.0 ± 0.6	
10 *S. subheptagyra* sp. nov.^1^ (n = 10)	23.7–26.4,	10.6–12.4,	6–6½
	25 ± 0.9	11.6 ± 0.5	
11 *S. hainesi*^1^ (n = 10)	21.6–28.1,	10.8–14.9,	6–7
	24.0 ± 2.2	11.9 ± 1.3	
12 *S. bocourti*^1^ (n = 10)	29.7–33.1,	13.8–16.1,	6–7
	30.2 ± 1.3	15.1 ± 0.8	
13 *S. inferospira* sp. nov.^1^ (n = 4)	24.8–29.3,	11.0–13.9,	6–6½
	26.8 ± 2.1	12.4 ± 1.4	
14 *S. melanospira* sp. nov.^1^ (n = 12)	24.3–29.3,	11.6–13.3,	6–6½
	26.0 ± 1.5	12.1 ± 0.5	
15 *S. pellosa* sp. nov.^1^ (n = 8)	20.8–24.7,	9.3–12.0,	6–6½
	23.4 ± 1.2	10.7 ± 0.8	
16 *S. dugasti*^1^ (n = 10)	14.6–17.2,	9.1–10.5,	6½–7½
	15.8 ± 0.7	9.7 ± 0.4	
17 *S. solemi* sp. nov.^1^ (n = 12)	21.0–26.5,	11.5–15.0,	6–6½
	23.2 ± 1.6	12.5 ± 1.0	
18 *S. gratesi* sp. nov.^1^ (n = 8)	14.8–17.4,	7.6–8.8,	6–7
	16.2 ± 0.8	7.9 ± 0.4	
19 *S. subcornea*^2^	12.5–13.6	6.0	7½
20 *S. benoiti*^3^	14.0–16.0	9.0	6
21 *S. pumicata*^4^	23.0–26.0	16.0	7
22 *S. ochtogyra*^5^	31.8	16.0	8
23 *S. rex*^6^	30.0	16.0	6½

**Table 4.** Shell morphology, mantle lobes, genitalia and spermatophore of *Sarika* species in Thailand. The visualisation of each character is linked to illustrations in Figures 2–4. Taxa in **bold** are the new species described herein. The superscript indicates spermatophore morphologies: ^1^ Shape of ridges on head filament (hf) of spermatophore. ^2^ Number of spines on tail filament close to sperm sac. ^3^ Length of terminal part of tail filament that contained spines / total length of tail filament.

Species	Shell shape	Body whorl	Left shell lobe	Inner penial sculpture	Penial verge	Ridges on head filament^1^	No. of spines^2^	Tail filament^3^
*S. resplendens*	depressed	well-rounded	present	cuboidal	absent	obtuse-serrate	3	1/3
*S. dohrniana*	depressed to conoid-depressed	rounded to slightly obtusely angulated	present	cuboidal	absent	smooth	2	1/8
*S. obesior*	depressed	well-rounded	present	triangular prism	absent	obtuse-serrate	3	1/3
*S. limbata*	depressed	well-rounded	present	reticulated / cuboidal	absent	plate-like with acute-serrate	3	1/3
*S. heptagyra*	strongly depressed	well-rounded to slightly shouldered	present	cuboidal	absent	n.a.	3	1/4
*S. kawtaoensis*	depressed to globosely depressed	well-rounded	present	reticulated / folded	absent	acute-serrate	3	1/2
***S. caligina***	depressed	well-rounded	present	triangular prism	absent	smooth	3	1/2
***S. lactospira***	depressed	slightly shouldered	present	triangular prism	absent	n.a.	n.a.	n.a.
***S. megalogyne***	depressed	well-rounded to slightly shouldered	present	triangular prism	absent	n.a.	3	3/4
***S. subheptagyra***	strongly depressed	well-rounded	present	triangular prism	absent	smooth	3	1/4
*S. hainesi*	depressed	obtusely angulated	absent	triangular prism	absent	sponge-like with obtuse-serrate	2	1/2
*S. bocourti*	depressed	obtusely angulated	absent	triangular prism	absent	acute-serrate	2	2/3
***S. inferospira***	strongly depressed	shouldered	absent	triangular prism	absent	n.a.	2	*
***S. melanospira***	depressed	rounded to weak shouldered	absent	triangular prism	absent	n.a.	n.a.	n.a.
***S. pellosa***	strongly depressed to depressed	rounded to weak shouldered	absent	triangular prism	absent	n.a.	3	1/3
*S. dugasti*	globosely depressed	well-rounded	present	smaller cuboidal / reticulated / larger cuboidal	pseudo-verge	n.a.	n.a.	n.a.
***S. solemi***	depressed	obtusely angulated to angulated	present	irregularly short folded /reticulated / cuboidal	pseudo-verge	n.a.	n.a.	n.a.
***S. gratesi***	strongly depressed to depressed	well-rounded	n.a.	n.a.	n.a.	n.a.	n.a.	n.a.
*S. subcornea*	depressed	well-rounded	n.a.	n.a.	n.a.	n.a.	n.a.	n.a.
*S. benoiti*	depressed	well-rounded	n.a.	n.a.	n.a.	n.a.	n.a.	n.a.
*S. pumicata*	depressed to conoid-depressed	obtusely angulated	n.a.	n.a.	n.a.	n.a.	n.a.	n.a.
*S. ochtogyra*	depressed	obtusely angulated	n.a.	n.a.	n.a.	n.a.	n.a.	n.a.
*S. rex*	Conoid-depressed	rounded	n.a.	n.a.	n.a.	n.a.	n.a.	n.a.

* *Sarika inferospira* sp. nov. has a tail filament with a series of branching spines along its length, except near the middle part which is spineless.

**Table 5.** Taxa attributed to *Macrochlamys* and *Sarika* from mainland Southeast Asia, as classified by their maximum shell width and maximum whorl numbers. Data are taken from the original descriptions and additional references. Taxa in **bold** are anatomically examined and have confirmed generic placements. The superscript indicates references: ^1^Godwin-Austen (1907) and Blanford and Godwin-Austen (1908), ^2^Laidlaw (1933), ^3^Solem (1966), ^4^Maneevong (2000), ^5^Sutcharit and Panha (2008), ^6^Pholyotha et al. (2018), ^7^Pholyotha et al. (2020a), ^8^Pholyotha et al. (2020c), ^9^ this study.

	Maximum number of whorls			
	whorl < 5	5 ≤ whorl < 6	6 ≤ whorl < 7	whorl ≥ 7
**Small size**	*M. kumahensis*	*M. bartoni*	*M. euspira*	*M. ramburianus*
**(shell width ≤ 10 mm)**	*M. patens*	***M. brachystia*^8^**	*M. jousoufi*	
	*M. pauxillula*	*M. brunnea*		
	*M. perpaula*	*M. callojuncta*		
		*M. cauisa*		
		*M. curvilabris*		
		*M. hatchongi*		
		*M. noxia*		
		***M. petasus*^8^**		
		*M. poongee*		
		*M. rejectella*		
		*M. salwinensis*		
		*M. spreta*		
		*M. subpetasus*		
**Medium size**	–	***M. aurantia*^6^**	***M. aspides*^8^**	*M. notha*
**(10 < shell width < 20)**		*M. chaos*	***M. caverna*^6^**	***S. concavata*^8^**
		***M. coleus*^6^**	*M. excepta*	***S. consepta*^8^**
		*M. declivis*	***M. lemma*^6^**	***S. dugasti*^4, 9^**
		*M. hypoleuca*	*M. nebulosa*	*S. gratesi* sp. nov.
		*M. malaccana*	*M. stenogyra*	*S. subcornea*
		*M. psyche*	*M. stephoides*	
		***M. tanymentula*^6^**	*S. benoiti*	
		*M. zero*		
		***S. nana*^7^**		
		***S. planata*^2^**		
**Large size**	–	*M. douvillei*	***M. kelantanensis*^6,8^**	*M. despecta*
**(20 ≤ shell width < 30)**		*M. glyptorhaphe*	*S. birmana*	***S. hainesi*^9^**
		*M. tenuigranosa*	***S. caligina* sp. nov.^9^**	***S. heptagyra*^9^**
			***S. inferospira* sp. nov.^9^**	***S. kawtaoensis*^9^**
			***S. khmeriana*^7^**	*S. pumicata*
			***S. lactoconcha*^7^**	
			***S. lactospira* sp. nov.^9^**	
			***S. limbata*^9^**	
			***S. lopa*^8^**	
			***S. megalogyne* sp. nov.^9^**	
			***S. melanospira* sp. nov.^9^**	
			***S. obesior*^8,9^**	
			***S. pellosa* sp. nov.^9^**	
			***S. resplendens*^1, 9^**	
			***S. solemi* sp. nov.^3, 9^**	
			***S. subheptagyra* sp. nov.^9^**	
**Very large size**	–	–	***S. dohrniana*^9^**	***S. bocourti*^9^**
**(shell width ≥ 30 mm)**			*S. rex*	*S. ochtogyra*

*Sarika resplendens* is regarded as one of the most common and widespread snail species in Thailand and this species is believed to have been accidentally introduced by human activities. However, most of the *Sarika* species have narrow distributional ranges restricted to individual habitats such as sandstone, granite, limestone or forested mountainous areas, and they show allopatric and sympatric distribution patterns, possibly resulting from limited dispersal abilities and the complex geography of the areas. The low dispersal capacities and a narrow ecological niche of land snails may reduce genetic exchange between populations (Shimizu and Ueshima 2000). A conspicuous feature of Thailand is the many extensive ranges of limestone karsts and outcrops scattered throughout the country (Gupta 2005; Naggs et al. 2006; Ridd et al. 2011). Limestone karsts in the humid tropics contain a high diversity of microhabitats that are conducive for land snail speciation and endemism (Clements et al. 2006; Foon et al. 2017).

The subdivision of *Sarika* into three groups (*resplendens*, *hainesi*, and *dugasti*) has been not yet resolved. Therefore, future studies combined with other genetic markers for molecular phylogenetic analyses will be necessary to clarify these subdivisions and may reveal a hypothesis of the evolution and biogeography of this genus.

## Supplementary Material

XML Treatment for Sarika

XML Treatment for Sarika resplendens

XML Treatment for Sarika dohrniana

XML Treatment for Sarika obesior

XML Treatment for Sarika limbata

XML Treatment for Sarika heptagyra

XML Treatment for Sarika kawtaoensis

XML Treatment for Sarika caligina

XML Treatment for Sarika lactospira

XML Treatment for Sarika megalogyne

XML Treatment for Sarika subheptagyra

XML Treatment for Sarika hainesi

XML Treatment for Sarika bocourti

XML Treatment for Sarika inferospira

XML Treatment for Sarika melanospira

XML Treatment for Sarika pellosa

XML Treatment for Sarika dugasti

XML Treatment for Sarika solemi

XML Treatment for Sarika gratesi

XML Treatment for Sarika subcornea

XML Treatment for Sarika benoiti

XML Treatment for Sarika pumicata

XML Treatment for Sarika ochtogyra

XML Treatment for Sarika rex
